# Methuselah/Methuselah-like G protein-coupled receptors constitute an ancient metazoan gene family

**DOI:** 10.1038/srep21801

**Published:** 2016-02-26

**Authors:** Alexandre de Mendoza, Jeffery W. Jones, Markus Friedrich

**Affiliations:** 1 Plant Energy Biology Australian Research Council Center of Excellence, School of Chemistry and Biochemistry, The University of Western Australia, Perth, WA 6009, Australia; 2 Department of Biological Sciences, Wayne State University, 5047 Gullen Mall, Detroit, MI 48202; 3 Department of Anatomy and Cell Biology, Wayne State University, School of Medicine, 540 East Canfield Avenue, Detroit, MI 48201.

## Abstract

Inconsistent conclusions have been drawn regarding the phylogenetic age of the *Methuselah*/*Methuselah-like (Mth/Mthl*) gene family of G protein-coupled receptors, the founding member of which regulates development and lifespan in *Drosophila*. Here we report the results from a targeted homolog search of 39 holozoan genomes and phylogenetic analysis of the conserved seven transmembrane domain. Our findings reveal that the *Mth/Mthl* gene family is ancient, has experienced numerous extinction and expansion events during metazoan evolution, and acquired the current definition of the Methuselah ectodomain during its exceptional expansion in arthropods. In addition, our findings identify *Mthl1*, *Mthl5*, *Mthl14*, and *Mthl15* as the oldest *Mth/Mthl* gene family paralogs in *Drosophila*. Future studies of these genes have the potential to define ancestral functions of the *Mth/Mthl* gene family.

G protein-coupled receptors (GPCRs) represent one of the largest and most diverse receptor type protein families in animals^1^,^2^,^3^,^4^. Leveraging the access to complete genome sequences from all major branches of the tree of life, previous studies have elucidated many fundamental aspects of GPCR gene family diversification^5^,^6^. Important subfamilies, however, remain insufficiently defined. One such example is the *Methuselah*/*Methuselah-like* (*Mth*/*Mthl*) subfamily of GPCRs, named after the *Drosophila* GPCR gene *methuselah* (*mth*). Discovered due to its effects on lifespan and cellular stress resistance in adult flies^7^, the *mth* gene is also essential for embryonic development^8^,^9^. The molecular mechanisms underlying *mth* function have received major attention, leading to the discovery of candidate ligands^10^,^11^, the identification of Mth-specific small molecule inhibitors that reproduce the *mth* phenotype^12^,^13^, and the recent finding that *Drosophila* Mth signals through the TOR pathway^13^.

In contrast to this significant progress, evolutionary age and functional conservation of the Mth GPCR have remained subject to debate. Indeed, little is known yet about the functions of its 15 paralogs in *Drosophila*: *Methuselah-like (Mthl) 1–15.* Molecular phylogenetic studies revealed that most of these genes originated during two gene duplication surges in the past 60 million years of fruit fly species diversification^9^,^14^. Remarkably, *mth* itself represents one of the youngest descendants of this exceptional gene family expansion, raising the question of whether its roles in development and life history regulation are likewise of recent origin or represent conserved remnants of ancestral functions.

Further support of a more recent origin of *Drosophila mth* function comes from the challenge in detecting *Mth*/*Mthl* homologs outside insects. In line with the canonical organization of GPCRs, *Mth*/*Mthl* receptors are composed of an N-terminal ectodomain, a seven transmembrane domain (7TM), and a short intracellular C-terminal domain. Early studies identified *Drosophila mth* as a member of the Secretin/Adhesion Class B superclade of GPCRs based on the 7tm_2-specific configuration of its 7TM domain^4^,^5^,^15^. The Mth ectodomain, by contrast, was recognized to be novel and only conserved in higher Diptera^5^,^7^,^9^,^16^. Later studies identified GPCRs with detectable Mth ectodomains in other insect orders^17^, and possibly arthropods^14^, but not beyond.

Importantly, the *Drosophila* repertoire of *Mth*/*Mthl* gene family members also includes four paralogs that lack significant similarity to the Mth ectodomain, but were identified based on conserved sequence signatures in the 7TM domain: *Mthl1*, *Mthl5*, *Mthl14*, and *Mthl15*^9^,^16^. The 7TM domain has thus been recognized as a more conserved sequence region of the *Drosophila Mth*/*Mthl* gene family, which includes both Mth ectodomain-positive and -negative members.

The significance of the 7TM domain is further underlined by the fact that some 7TM domain-based studies reported evidence of candidate *Mth*/*Mthl* homologs in metazoan species outside arthropods: the sea squirt *Ciona intestinalis*^18^, the lancelet *Branchiostoma floridae*, and the sea anemone *Nematostella vectensis*^5^. At the same time, no candidate *Mth*/*Mthl* homologs were noted in the first genome-wide survey of GPCRs in the lancelet^19^ or in similar studies of the acorn worm *Saccoglossus kowalevskii*^20^ and flatworms^21^, possibly due to incomplete genome sequence coverage or sampling. Finally, a recent study of GPCR diversity in insects expressed doubt about the monophyly of the *Mth*/*Mthl* subfamily^22^.

These confounding data leave three major questions: How deeply conserved is the *Mth*/*Mthl* gene family in the metazoan tree of life? Is the Mth ectodomain an ancestral or derived component of *Mth*/*Mthl* gene family members? And which are the most ancestral *Mth*/*Mthl* homologs in *Drosophila* that could give insights into the earliest functions of the *Mth*/*Mthl* gene family?

## Results and Discussion

### Early metazoan origin of the *Mth/Mthl* gene family

To improve our understanding of the *Mth*/*Mthl* gene family, we investigated the phylogenetic position of previously and newly identified candidate homologs. To this end, we searched a database of 39 genomes representing holozoan species diversity (Ichthyosporea + Corallochytrium + Filasterea + Choanoflagellatea + Animalia) (Supplementary Table S1) by BlastP with the 7TM domain of *D. melanogaster* Mthl1 as query and collected the 100 best hits as candidate homologs. We then used the best matching human candidate homolog (GPR98) from this pool as query for a second search, from which we collected an additional 100 best matching sequences after removing duplicates found in the first search. In parallel, we identified candidate homologs from species without complete genomes based on reciprocal BLAST evidence using the previously reported Mthl candidate homolog from *N. vectensis* as query^5^. Combined, these efforts resulted in a total of 278 GPCR sequences (Supplementary text file S1), which were used to build a multiple alignment of the 7TM domain (Supplementary text file S2) for molecular phylogenetic gene tree estimation using Bayesian and likelihood approaches.

**Figure 1:**
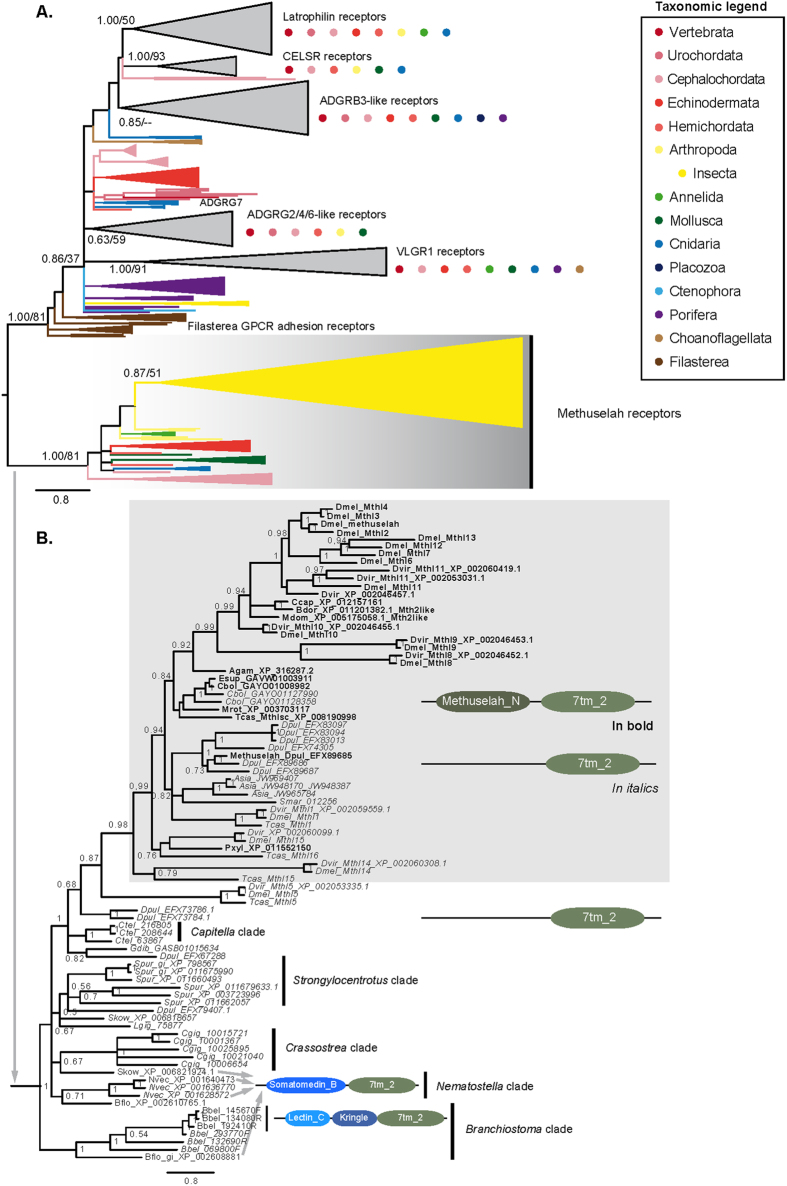
Phylogenetic analysis of the relationships between *Mth/Mthl* and related holozoan GPCRs. Nodal numbers represent Bayesian Posterior Probabilities and Maximum Likelihood bootstrap supports (100 replicates). (**A**) Collapsed Bayesian phylogenetic tree estimate. Clades were collapsed using congruent nodal supports by both tree estimation methodologies, defining stable clusters and conserved PFAM domain architectures^30^ as a secondary evidence for family classification. Orphan GPCR gene families were kept unclassified. The holozoan phyla represented in each GPCR group are indicated by the color described in the taxonomic legend box. (**B**) Uncollapsed view of the *Mth/Mthl* GPCR gene family cluster in panel (**A**). Grey background box denotes the expanded insect cluster of *Mth/Mthl* genes. Detectable Pfam domains in specific genes indicated to the right. Species abbreviations: Agam = *Anopheles gambiae*, Asia = *Argulus siamensis*, Bbel = *Branchiostoma belcheri*, Bdor = *Bactrocera dorsalis*, Bflo = *Branchiostoma floridae*, Cbol = *Cordulegaster boltonii*, Ccap = *Ceratitis capitata*, Cgig = *Crassostrea gigas*, Ctel = *Capitella teleta*, Dpul = *Daphnia pulex*, Dmel = *Drosophila melanogaster*, Dvir = *Drosophila virilis*, Esup = *Epiophlebia superstes*, Gdib = *Glycera dibranchiata*, Lgig = *Lottia gigantea*, Mdom = *Musca domestica*, Mrot = *Megachile rotundata*, Nvec = *Nematostella vectensis*, Pxyl = *Plutella xylostella*, Skow = *Saccoglossus kowalevskii*, Smar = *Strigamia maritima*, Spur = *Strongylocentrotus purpuratus*, Tcas = *Tribolium castaneum*.

Consistent with previous studies, the resulting trees recovered a robustly supported clade of arthropod *Mth*/*Mthl* homologs (Fig. 1A and Supplementary text files S3 and S4). This clade was nested within a likewise robustly supported, more inclusive clade. Altogether, our trees identified 44 *Mth*/*Mthl* candidate homologs from diverse animal species outside insects. These included protostomes (Annelida, Mollusca), invertebrate deuterostomes (Echinodermata, Hemichordata, Cephalochordata), and the sea anemone *N. vectensis*.

To scrutinize the non-insect candidate homologs, we performed reciprocal BLAST searches against the NCBI nr protein sequences of *D. melanogaster* and *B. floridae*. This approach defined 24 high confidence *Mth*/*Mthl* homologs outside insects, which included 10 other arthropod, 4 mollusc, 7 hemichordate, and 3 cnidarian genes^5^ (Supplementary Table S2). Our targeted, double pronged approach thus corroborated the previously suggested presence of *Mth*/*Mthl* homologs in sea squirts^18^, lancelets, and cnidarians^5^ and, for the first time, detected *Mth*/*Mthl* homologs in the acorn worm *S. kowalevskii*^20^.

### *Mth/Mthl* gene family extinctions and expansions

Within chordates, our analyses only detected *Mth/Mthl* homologs in the lancelet species *B. floridae* and *B. belcheri* (Fig. 1). To further explore the apparent absence of the *Mth/Mthl* gene family in other chordate clades, we searched the NCBI nr database by reciprocal BlastP for tunicate and vertebrate homologs using the *B. floridae Mth/Mthl* homologs XP_002610765 and XP_002608881 as queries (Fig. 1). Also this approach failed to detect *Mth/Mthl* homologs in either tunicates and vertebrates, leading to the conclusion that the *Mth/Mthl* gene family became extinct during early chordate evolution and was absent in the last common ancestor of urochordates and vertebrates.

Further losses of the *Mth/Mthl* gene family during metazoan evolution were indicated by absence of detectable *Mth/Mthl* homologs in genomes from Nematoda, Platyhelminthes, and Rotifera. A particularly prominent example in addition to vertebrates was the absence of *Mth/Mthl* homologs in the cnidarians *Acropora digitifera* and *Hydra magnipapillata* in contrast to the presence of three *Mth/Mthl* homologs in *N. vectensis* (Fig. 1 and Supplementary Tables S2 and S3).

Our trees also detected multiple independent expansions of the *Mth/Mthl* gene family in the metazoan tree of life. The most dramatic example of this continues to be the exceptionally enlarged *Mth/Mthl* gene family cluster of insects (Fig. 1)^9^,^14^. Other examples are found in *Daphnia pulex* (Crustacean), *Capitella teleta* (Annelid), *Crassostrea gigas* (Mollusc), *Strongylocentrotus purpuratus* (sea urchin), and *N. vectensis*.

While these repeated extinction and expansion events explain the previous difficulty of recognizing the ancientness of the *Mth/Mthl* gene family, they continue to pose a challenge to reconstructing the ancestral set of *Mth/Mthl* genes in the last common ancestor of Metazoans. At this point, the scattered conservation of *Mth/Mthl* homologs in distantly related lineages is best explained by the presence of a singleton homolog in the last common ancestor of Eumetazoans (Bilateria + Cnidaria).

### Derived acquisition of the Mth ectodomain

The Mth ectodomain is defined by 10 cysteine residues, which form a total of five disulfide bonds^7^,^16^. Most of these residues were noted to be conserved in the *Drosophila* paralogs Mthl2*–*4, and Mthl 7*–*12 but not in Mthl1 and Mthl5^16^. Consistent with this, we were able to confirm the presence of the Mth ectodomain for Mthl2*–*4, and Mthl 7*–*12 in Pfam database searches based on significant matches to the Methuselah_N domain PF06652 with e-values between 8.9E-18 to 3.1E-69 but not for Mthl1 and Mthl5 (Supplementary Table S3). Moreover, Pfam support for the presence of a Mth ectodomain was exceptionally low or non-significant for the more recently described *Drosophila* paralogs Mth15 (Pfam e-value: 0.00012) and Mthl14, respectively.

To address the question whether the presence of the Mth ectodomain is a derived or ancestral feature of *Mth/Mthl* gene family members, we searched all other members of the 7TM domain supported metazoan *Mth*/*Mthl* gene family clade for the presence of the Mth ectodomain. Consistent with the Pfam database listing of the Methuselah_N domain (PF06652) as arthropod-specific (Finn *et al.* 2014), this approach detected additional Mth ectodomains only in *Mth*/*Mthl* homologs from arthropod species (Supplementary Table S3). Almost all of these represented insects except for the previously reported custacean *Mth/Mthl* homolog EFX89685 from *Daphnia* (Pfam e-value: 0.00012)^14^.

Mapping the conservation of the Mth ectodomain onto the 7TM domain-based *Mth*/*Mthl* gene family tree further revealed that all Mth ectodomain-positive homologs are contained in the strongly expanded arthropod subcluster of *Mth*/*Mthl* homologs (Fig. 1B) and that this clade is sister to the Mth ectodomain-negative homolog *Mthl5*. Since *Mth/Mthl* gene family members outside arthropods are all Mth ectodomain-negative, outgroup rooting supports the model that the Mth ectodomain is a derived domain that has been acquired during arthropod evolution.

Interestingly, our Pfam searches detected evidence of additional ectodomain acquisition events in the *Mth*/*Mthl* gene family tree. This included the presence Somatomedin_B domains in the *Mth/Mthl* homologs XP_001628572 of *N. vectensis*, XP_002608881 of *B. floridae*, and XP_006821924 of *S. kowalevskii* (Fig. 1B). The evolution of the *Mth*/*Mthl* gene family may thus have been repeatedly shaped by ectodomain acquisition events.

### Candidate ancestral *Mth/Mthl* receptors in *Drosophila*

51 *Mth/Mthl* gene family members in our trees possess ectodomains that lack significant similarities to any currently known protein domains. This includes *Mthl1*, *Mthl5*, and *Mthl14*, which, according to our trees, represent the oldest *Mth*/*Mthl* gene family members in *Drosophila*, together with Mthl 15, which is characterized by only marginal Mth ectodomain support (0.00012). The phylogenetic evidence thus suggests that defining the ligand binding properties and physiological functions for these *Drosophila* homologs has the potential to elucidate ancestral functions of the *Mth/Mthl* gene family.

Interestingly, expression studies in *Drosophila* point toward a role of *Mthl5* in the development of the visceral and cardiac mesoderm^9^. Moreover, recent gene knockdown studies in the red flour beetle *Tribolium castaneum* produced evidence that *Mthl5* is essential for embryonic and postembryonic survival^23^,^24^, interacting with several signaling pathways^25^. In combination, these first insights and the strong gene tree evidence of an ancestral status of *Mthl5* prioritize this paralog for further study.

### Conclusions

Taken together, our studies lead to four important conclusions: (I) *Mth*/*Mthl* homologs are present in cnidarians, protostomes, and deuterostomes, implying an early metazoan origin of this gene family. (II) The *Mth/Mthl* gene family is characterized by numerous extinction and expansion events, reflected most notably by its absence in vertebrates and abundance in insects. (III) The current definition of the Mth ectodomain most likely originated during the exceptional expansion of the *Mth/Mthl* gene family in arthropods. (IV) Mthl1, Mthl5, Mthl14, and Mthl15 represent the oldest *Mth/Mthl* paralogs in *D. melanogaster*, prioritizing these genes for the experimental characterization of potentially conserved ancestral functions of the *Mth/Mthl* gene family.

## Methods

The multiple sequence alignment was built using MAFFT, applying the L-INS-i method^26^ and manually cleared of gaps and non-homologous regions outside the 7TM domain. The trimmed alignment of 365 amino acid positions was subjected to phylogenetic analysis with PhyloBayes 3.0 until 2 chains converged (maxdiff <0.3)^27^ and to estimate the 20 best RaxML 8.0 trees^28^ using the LG model and 8 categories for gamma distribution and 100 bootstrap replicates. Pfam searches were performed at default gathering threshold^29^. Species lists, sequences, alignments, and trees are available in Supplementary Information.

## Additional Information

**How to cite this article**: de Mendoza, A. *et al.* Methuselah/Methuselah-like G protein-coupled receptors constitute an ancient metazoan gene family. *Sci. Rep.* **6**, 21801; doi: 10.1038/srep21801 (2016).

## Supplementary Material

Supplementary Information
