# Supplementary material for: Methuselah/Methuselah-like G protein-coupled receptors constitute an ancient metazoan gene family
Source: Sci Rep. 2016 Feb 26;6:21801. doi: 10.1038/srep21801 (PMC4768249; doi:10.1038/srep21801)
Supplement: Supplementary Information [file srep21801-s1.pdf]

Supplementary Information

Methuselah/Methuselah-like G protein-coupled receptors constitute an ancient metazoan gene family

Alexandre de Mendoza[1], Jeffery W. Jones[2,3], and Markus Friedrich[2,4]

1: Plant Energy Biology Australian Research Council Center of Excellence, School of Chemistry and Biochemistry, The University of Western Australia, Perth, WA 6009, Australia.

2: Department of Biological Sciences, Wayne State University,
5047 Gullen Mall, Detroit, MI 48202

3: Present address: Advaxis, Inc., 305 College Road East, Princeton, NJ 08540

4: Department of Anatomy and Cell Biology, Wayne State University,
School of Medicine, 540 East Canfield Avenue, Detroit, MI 48201

The Supplementary Information consists of 1 PDF document containing Tables S1-3 and text files S1-4

Text file S1: Sequences included in the analysis in fasta format.

>Dmel_methuselah
MKTLLVLRISTVILVVLVIQKSYADILECDYFDTVDISAAQKLQNGSYLFEGLLVPAILTGEYDFRILPDDSKQKVARHIRGCVCKLKPCVR
FCCPHDHIMDNGVCYDNMSDEELAELDPFLNVTLDDGSVSRRHFKNELIVQWDLPMPCDGMFYLDNREEQDKYTLFENGTFFRHFD
RVTLRKREYCLQHLTFADGNATSIRIAPHNCLIVPSITGQTVVMISSLICMVLTIAVYLFVKKLQNLHGKCFICYMVCLFMGYLFLLLDLWQ
ISISFCKPAGFLGYFFVMAAFFWLSVISLHLWNTFRGSSHKANRFLFEHRFLAYNTYAWGMAVVLTGITVLADNIVENQDWNPRVGHE
GHCWIYTQAWSAMLYFYGPMVFLIAFNITMFILTAKRILGVKKDIQNFAHRQERKQKLNSDKQTYTFFLRLFIIMGLSWSLEIGSYFSQSN
QTWANVFLVADYLNWSQGIIIFILFVLKRSTWRLLQESIRGEGEEVNNSEEEISLENTTTRNVLL
>Dmel_Mthl2
MIASSKMLLSASILIYFLLNLQSSSAEIADCSFYDTVDISEGQRLSNGSYLYEGLLIPAHLTAKYEFKLLANGDKEQVPSHVRGCVCKLRT
CVRFCCPHDHIMDMGECYANMTTEENELLDPMLNVTLDDGSVVQRHYKKELMVQWDLPKPCDDMFYLDNRDIMDEYTLFENGRLLR
HYDQVYLDKSEYCLQHRTFGEGNNNSIRIIPHNCLILPSRTGQTVVMITSLICLVLTIAVYLCVKKLMNLEGKCFICYMMCLFFGYLFLLLD
LWELSLDFCKAAGFLGYFFVMAAFFWLSIISRHYWKCLTNPCASMNIRSERAFLLYSCFAWAMPLALTGVTYLADNVVNNEEWQPRV
GDEGHCWIYTKSWSAMVYFYGPMVLLILFNITMFVLTAKHIIDSKRTLRKIARNEGRIQKLNSDKQNYTQFLLLFTVMGMSWSFEIFSYL
VQREKLWVNIFLVADYFNWSQGVIIFVLFILRRKTLVLFKKQIFPKQRAFSRSATQSTIESISQTKRHFNMT
>Dmel_Mthl3
MRIVIGSFTAFLLLLLQNSNAEIPGCDFFDTVDISKAPRFSNGSYLYEGLLIPAHLTAEYDYKLLADDSKEKVASHVRGCACHLRPCIRFC
CPQYQKMQKSKCYGDMSEDELNKHDPFVNVTLSDGSVVRRHFKEDLIVQSDLAKPGCPRMYFLNHELPGNEFTLFENGSLLRHWDK
VELSKREYCVQHLSFKDDSIRIAPHFCPLSSEHSRTWKTVAIVISLICIILTISVYLYVEKLRNLHGKCFICYLASLFLGYFFLVLNVWKYSS
GFCVTAGFLGYFSVIAAFFWLSVISLTLWNSFSGNSSWLNRFLPQNRFLSYNLYAWGMALLLTAITYIADQVVKNEKLRPRVGVGKNC
WIYTGDMTVMIYFYGPMLLIIVFNITMFVLTAFRIMKVKKEAQNFTQQQKTTNRLNSDKQTYALFLRLFIIMGLSWSLEIISFLLSKNQAWA
KAFMVADYFNWSQGTVIFLLFVLRPSTLKLLKERIKGGRDEAGASDEHISLQNTKIDPSVF
>Dmel_Mthl4
MRILLIAVLFLLMPKSNAEIPGCDFFDTVDISKAPRFSNGSYLYEGLLIPAHLTAEYDYKLLADDSKEKVASHVRGCACHLRPCIRFCCPQ
YQKMQKSKCYGDMSEDELNKHDPFVNVTLSDGSVVRRHFKEDLIVQSDLAKPGCPRMYFLNHELPGNEFTLFENGSLLRHWDKVEL
SKREYCVQHLSFKDDSIRIAPHFCPLSSEHSRTWKTVAIVISLICIILTISVYLYVEKLRNLHGKCFICYLASLFLGYFFLVLNVWKYSSGFC
VTAGFLGYFSVMAAFFWLSVIGIHLRIKFSLASNCLHRLLPENPFRAYNLYAWGIPLIMTAITYTADQVVKNEKLRPRVGVGKNCWIYTG

DMTVMIYFYGPMLLLIAFNIIMFVLSAIYIYNIKKNVKGLVHKQQTNQQINDQQMFAIFLRLFILMGLSWSFEILSFLLTKQQAWARALMVA
DYFNWSQGTIIFVLFILKPSILKLIIAGGRQNLPGSHHNSRSKAARYNSTHTACEGSIADPNAYC
>Dvir_XP_002046457.1
GLVLTLLFLEIVAAQIPGCDYFDTVDLSHSPKLSNGSYQYEGLVIPPEQTGEYDFEILADGEKESVPRHLRGCACRLGSCIRFCCHRNLF
LDRNERTCSGDIAKAVDYDPYVNISLANGTQVRRHV
LKDFIVQQDLPVPCSEHFHLDALNDESHGWTLMEDGRLLRHFDQKSLSKQEYCLQPHPISAGDNKTVITLVPHNCNDPPESQLLYNILR
LLSIICLLSTIIVYLFIPKLRNLHGCCFTCYMASLLIAYALLLVDSWKEDWSKSMCQLNGYVGYFAVMASFLWLTVISFDLWSSFRSNNYN
VQRYTPRYRFLIYSLYAWGVAALLTLIVIIVDYKLDDNDDDELFWMPGVGLYNCWVKTHDWSALLYFHGPMALQILFNIIMFILTAIRILEV
KRDLQNVAAHGEREQRLNSERQTYTLFMRLFVIMGVTWTFEIFAYFAQNQKILEKIFSFFDYINCAQGVIIFIMFVLKSSVLRLISNRIRGIE
SAEDGSDSEEEIALQDRNGAVNKIGPNILN
>Dvir_MthI11_XP_002053031.1
MADISGCDYFDTVDLTNSHKFENGTYLYEDILIPKEKVGLYDYQILFNGDREPVPEHTRGCACQIKSCVRFCCDPQKLLVKGEGICEGNI
NLNYSSILNITMHDGAEVEKDVMEFIVQKHLPVPCNDHLMLNAAGNENHGWTLFENGTLVRHFDGEHLSKRDYCLQPIHRPNSQLLYE
LQPHHCLPPTEKTNAYIQTVSIFCLAIIIVVYLYLPNFKSIHGKCCTCYFTCLTASFLMIVVVSFGWVDKKYSLICFLIGYSGYYAIMATFLW
LLLINYNLWKTFNNIGVGRRSRFMNYNIFVWSVAAIFLMITCLADFLYEVDENEEDPNMFIFKPGVGLYSCWINIYDVSAMIYFYGPILLLI
VCNTTFFIKTAMRIFVQNKNNKRQLKKTECQHNLRNLTNFEMFFRLFIIVGVIWFLEIISYLCTLFNVNSMWVNFADMLTSGQGVILFVVTI
IKKDVLKPIAER
>Dmel_MthI11
MGMFRVEYLLLGILVIGVRSRDIPNCDFFDTVQLRESEKLCNGSYRYEDVVIPAKLTGKYDYEIDYDGDRVSVPKHIRGCVCKLKTCIRF
CCHHKKLMAGNLCSQDVYENLTYEYTLDITQLNGSVIKKHVLNDMVVQQDLPLPCERHYSLDAETSTYDMWSLYENGSLFRHFDQRY
LSKQEFCLQPNPTSTGKNYSLIVAFNCIQKPSMKMAYGRFECVRKSRLSNASIPVKFSSVFFMVITIAAYLWLPKFRSLHGKCCNLYFIC
LAITFLLNVISLFGIFELKTPICYLTGYAGYFTVMATFLWLSVISFDVWRRFAMRKFQVFYKNKRSSFFNYNIIVWSSAGLLTCIIFLVDQFV
ETNLDNPYNPAVGVFSCWIFTNGWSATFYFYAPLAILIILNCASFFLTTRYIYVENKQNQKVLNNSEPQKLSRNHANYRIYFRLFIIMGGS
WFLEIIAFICEMENMWKPLIILNDYINCSQGIIIFVATFCNHEMFRLIRKRIQNRNITSLELTNTSRPVESEKMADVELGK
>Dvir_MthI11_XP_002060419.1
MGSSSWALLVLGVFLVQSTADIPGCNYFDTVKLTQEQQLENGSYRYRNVIIPKEKTGIYDYRISLDGKMQMASNYTRGCVCHVKSCIPF
CCDPHEFLVDALLNVTLFNGTETEINLRKDFLVQPVYEEFCDFIGLNSSLDHQWTLFENGILFKHTNNHNKNKGRYCLVPLAVNDDVYK
LGAHYLAESSSTKSTITLPDALRVISIFCMIITIATYLYFPKFRSVYDKCCICYFFCLAVSFTLLWCESLYRESIPFKWVCNFVGYIGYYTVM
ATFLWLLMINFTLWKKFKNLGIACNSNFKKYNIIVWSVSAGLLGITLLVAYLFEMDENFDVDNKSQWQPRIGYFSCWIETEDYSAMIYYY
GPILLAILINIWLSIASAMIIYREDQNNCRVWSNAESSNKLAHQAKQVIFIDLHVYRFLSYFHLVSNYSSVYSP
>Dmel_MthI6

MLLNILAIILVFVISSQSEAVIPGCDYFDTVDISHIPKLNDSYAYEELIIPAHLTGLYTFRQLADGSQEPVKSHLRACICKLKPCIRFCCPRNK
MMPNSRCSDGLTENLKRINPYLKITLEDGTIGKYYLLTDMIVLRYEFRYCEKVVSVQEDQYKLYENGSFMIKPDVNWTLSKQWYCLHP
RLEDPNSIWILEHVYIPKSMPAVPQVGTISMVGCILTIAVYLYIKKLRNLLGKCFICYVFCKFVQYLIWAGGDLNLWNNICSLAGYTNYFFA
LASHFWLSVMSHQIWKNLRLINRDERSYHFLIYNIYGWGTPAIMTAITYLVDWAWEDRPDKLNWIPGVGLYRCWINTYDWSAMIYLYG
PMLILSLFNVVTFILTVNHIMKIKSSVKSSTQQQRKCIQNNDFLLYLRLSVMMGVTGISEVITYFVKRHKFWRQVLRVPNFFHLGSGIVVF
VLFILKRSTFQMIMERISGPRRQQPAS
>Dmel_Mthl7
MRLPWVIFCTVLLLIFTNNSNADIPGCNYYDTVDISYIERQNDSYLYDDIEIPASLTGYYEFRQFGDGSITPIEKHLRACVCSVRPCIRICC
PAKNFLANGKCDDGLKEELARFKPYIYFTYMDLQARVPLTDMAIIRDEFFDCDEMIYISDFNYFLEEDGKFWVTVDLFMEKQDYCLYRH
NFDSDFPKSMWIIRHRCTSHISPGSLEILIITMICFVLTIAVYLYIKKLRNVTGKCIVCCIVSRFIQCLIMILDHLNLLNGICSPAGYSSHFFRM
ASNLWLSVISYHTWKVLTSLNRVDPNYRFLRYNAFVWSTAAIMTGSIYIVNQIWENDPSKWNWLPLVGFIRCSVKDWHPSVWIYISGPS
LALSTFNVAMFALTAIYIRKVKGGINKFTNEEEGRINCINFDSQTYLQFLRLSIVMGLTWIFNVIPYSARLHIFWEWVGIISEYFHSAFGIVL
FVLLVLKRSTWTLMMDS
>Dmel_Mthl12
MFLWLKCFCTLIIVTIAKNSSAKIPHCKYDETINISHFKRLNDAYIYEHFEIPANLTGEFDYKELMDGSKVPTEFPNLRGCICKVRPCIRICC
ARKNILSNGECSDGVKNEIKLTMLDLTMQDILLTDPTLAELNMIPQYNSTELLILREQFQPCDEIVSLKRDEYTILKDGSILLHTSAEILSND
QYCLYPEIYSDFPETIRIINRRCYRNVMPGIAQLSVISVVGFILTLAVYLSVEKLRNLLGKCLICSLFSMFMEYFIWTMDYFRLLQSICSAAG
YMKYFFSMSSYLWFSVVSFHLWELFTSLNRHEPQYRFLIYNTFVWCTAAIPTVVIFSMNQMWENDPGKSEWLPLVGYFGCSVKDWN
SSSWFYSHIPIVILNSFNVIMFVLTAIYIWKVKKGVKSFAQHDERNTTCLEFNVQTYIQFVRLFLIMGASWLLDQLTRLAEDSHLLLDTIVL
NLTVYLNAAFGILIFVLLILKGSTFKMIMER
>Dmel_Mthl10
MPKKIHQPGGSLYCGVTLLGVLCLVVFRLIPGIPFGTYVMAERDHYHTIDDPNVPCNFYDTVNLTGHRLFPNGSYDYYGTIVPAELVGT
YDYIHSSLTERIEVREHVRGCVCKFKSCLNICCPWRQVFNSEVDGCIIDHSDNRTWPDPPMLNITFRNESTILVNMFTQFAIQSFRPCPK
MFSLQPETNNWDDYLLFENGSMLRVDDKLLIRKNEFCMVPTYVNESDMFYTIHPANCDMQDDHSTVKIINSYAMMFSIPFMMLTIAVYL
LIPELRNQHGKSLVCYLIGLSVGYSSLCYVQLYQVDATGVTCKVFGYTAYFFFMGAYMWLSVISFDLWHNFRGTRGINRFQEKKRFLF
YSLYSWGIALVFLAFTYCAQQLTNLPANLKPGIGDGVYCWLDMSNWAAMIYFYGPILAIVVANTIMFIMTAIKIHGVQREMARIIASENST
KNLRTEKDKFGLFLRLFLIMGITWLTELISYFVGSDKGWSKLFYISDLANAMQGFLIFMLFVMKKKVKHLITNRCSSVRDGSNQRQSQYS
TKTTSSSVANLSLHEKPSVEKPLVISSSVDPQKTTIFR
>Dvir_Mthl10_XP_002046455.1
MQQHHRSGSLYCGVTLVGVTLLVLLRLMPGSPFGVYAMVEGGHYHERNGIHTKCSFFDTVNLTGYPSYPNGSYSYEGVLIPSHYVGT
FDYIYKDLVDRVDVAPHMRGCICKLKPCINICCPWGQIYNNSECQKDTNTKQMWPEPTINITLNNGSVQNVNIYEQFVVQSFRPCTEMF

SLLPEINYYDTYQLYENGTLLREDDMHYIYKNEFCLVPTNINDTDLYYTINPANCDMFNENTTVKIINGFAMLFSIPFMLLTIAVYLLIPELR
NQHGKSLLLGYTAYFFFMSAFFWLNVISFDLWHNFRGTRGINRFQEKKRFLLYSLYSWGLALVFLVFTVLVQEYFDWPEALKPGIGAG
QFCWLDMTNWSAMLYFFGPILIIIVANTVMFMMTAIKIHGVQREMARIIAREDSTKNLRTEKDKFGLFLRLFLIMGVTWSSEIISYFVGND
KKWSKIFYVTDLCNAMQGFLIFMLFVLKKKVKHLITNRTVRVRSLRQTESVTMAGSSYTMRQRFMDASQHIRKLVVIRNAFANTASGVH
SHAV
>Bactrocera_dorsalis_|XP_011201382.1_Mth2like
MFGQISLLLFGVCLLFGNAQSALSEIPNCVFEDTVNLTASTKFGNGSYLHEGLLVPAHLTGRYDYIELYDGERKQVEPHVRGCVCQLKK
CVRFCCHPRADMYRLSKDSPSLCAEELNEELKYSPYVNVTLHNSSRVLMHVLDEFVVQQGIPCTDGYMLMPHMYEEDQWELREDGT
LYRLADAQV
LSRREYCLQSYAVGNTYVLNPMNCPMTYEPPPTIMLNTIIMLISAPFLYITILVYWLIPELWNLHTKCLICYLISLAVGTTLIVMVNMREANY
ENTICGIIGFVAYYFLTAVFFWLNVVCYDVWQNFCRSKGNVQHLSQRKQFMYYSLYGWGLPALLTVLTMGLQYSNLPIYLKSGIGYSH
CWLKTDDWSAMIYFYGPCLLLIIFNIAIFVLTIKKIYKMRSEMQTSVESKDSRRKLRSHKRNVWLFFRLFTVMGIGWLLEIIGYMVGNNSD
YTIFFQIADVYNAAQGLIIFAILVLKKKVLLLIKKRILCTKSSDATDSAESQCSEEEIALRSMTNEVPQILK
>Ceratitis capitata_XP_012157161.1_Mth2like
MMATHIFLLYLGTLWSLFSDSKADIPNCAFEDTVNLTAREKFTNGSYIYEGLLVPPQYIGEYDYIELFEGARQKVEKHTRGCVCKLKQCV
RFCCHPRADMFQSSEHASPQCDNEQLSDEPKYTPFVNVTLRNNSRVSMHLLEEFVVQQGTPCADVYPLMPHVYEEDKWELFENGTL
LRDRDYLSRRDYCFQAYNFSGEFVLNPMNCPSMYSEPPTMMLNTKIMMISAPFLFATILIYWLIPELWNLHTKCLISYLLSLAVGTSMIVV
VNVQNSDFEDLNCSIIGFITYFFLTAVFFWLNVICFDLWHNFRRAKGGAQHLSQRKRFLCYSLYAWGLPTIMTIVTASLQNSKLPHALKS
GIGDTHCWLKVDDWSALLYFYGPCLLLIIFNISIFVLTAKKIYSIRKELQHFSRGEDSRRHLHSHQNNVWLFFRMFIVMGVGWLLEIISYM
FGNYNKLAFFFAATDVYNASQGIIIFILLVMKKKVLLLIKKRLCKTDKKQQFSSRRYAKSSTSAVELSNAEYRWKPRN
>Mdom_XP_005175058.1_Mth2like
MHLKTTLHVILACTFFAFCMGSGDIIPKCAFEDTVDLTKSQKFNNGSYLYEDILITPDQINIYDYEEIYQGIRLPREPHPRACLCQGQRSC
VRFCCHPARELIANYSRRCSGEMLQKELEYDPYINVTLNNGSVVSKHVTKEFTVIQGVPCAGAYALAPQYDEEDSWVIFENGTLLRHY
DMAYLSKRDYCLLPMELDTGEWVLNPMNCPILNEASLSLQINNIAMAVSVPFIILTIIVYVFIPELRNLHGKCLISYLCPLAVGYSILSTISLS
EVVFPNLVCSCLGYVAYFCFMSAFFWLSVISFDLWQNFRLTGSMRFGQRKRFCLYSIYAWGIPACLTLIVIALQNSNMNELYKPGIGDN
YCWLKTNDWSAMMYFFGINLIIVLIDITFFTMTYFKITVMQRDIDKIIRNDTIEGRHQLRTHKNNFGLFLRLFLIMGVSWLLDIVSYIETIVYP
NDINPIYYVSDFMNAILGLLIFVCFVLKPKVLSLIKKRCQQTKGGAQMRPMVKRTTTSQLSLTNIKMTPIPTRCSNMQS
>Dmel_Mthl13
MKLQLLGTFLVLILLQFQAVNTDSNKTEEDSCVKSDDDNSNDEDDLWHCIPICCPRKNMMANGGCSFKEHLFRTKLDLNIRLDDNSTE
ALYFNNQTLLITSFWDIDEMMGLRRDEYTLYKNGTIYIHSDQSIQTKEEYCFYPHQIYSDFPETIWIISHKFSSIESPGAFELASGSIICYIIIF
GIYLFVKELRNDFGKCVMSCVFCLFLDYLIWLMDELRLLDDFCSLTGYIMFFFDFANSVWFSIISYCIWKKITSVVSQENRDQFVFYSTFA

YGISAIPLGIIISINQFWEEDLRKWNWLPLVGFSRCSRSSWVYYFVPYAIMCAINIIMFVLTIKHIMKTKRNLHNLTKRPDRNETCVTVNFL
NFEYLLFLRISGIMGVAWILIIFLLIEVNSTFWDIFGIIIQQIHYGFGIILFVLLIFKRSTHQLLIAK
>Dpul_EFX89685
MTKSKAEIFGELSLRWIDIHRDQLLISICARVWQSIAFESANNAKRPSRDTQSYHGGGVAICIHKLSDRVTTIPKTLSLGAPSKRKSNRRV
KKKKKRKSQHRPRGDPIPPGSSSYSSVPCGTPNWERRLSRASIFQRVETLVLVSCFKKLRHSMMAIKWLVAIVTLLCAAHFNDGQNLL
SKPDPQLEWQIPLTKCCASENEFYSLGFDTCTLNGEMNFNWPPPVYSARTNESVSAASARFSLTFNLSTCTSGYASQSTRDFRLYTD
GSAVISSSGERLPANSFCLNQISSGEADAAEFAVRHCASDPCNQTNCIRKCCPLGMALNTTTQLCQTYNEPFALEFRNVTGHVVTPNP
ASYLIRQGDAPKCQHGMFPLSPNTNPEDEFYVLPDGQIYLPYYPENDRYTRDYCIDDFSSEEGIYRQALLCFPPQPEESADNQLVLKVF
PYFLFLSSLFLIATFVVYAMIPEIRNIHGVTIMCHVASLAVMYIGLCVIQLGPQLPDGV
CVGLAVIVHFTFLSTFTWLNVMSFDIWWTFSPLNCSTNAKAVAVAFWMQTLANQTTSSASSSPSTDDDECAKENSDEDSGPNQSLISS
CDLRPRNSRHGRQHLGRRIIFYSIYAWGVPFIIVLVGQVLDHLKNLPQHIVTPGFGEVKCWFFEKEAFLVYLYGPIAVLILSNIVFFVMTA
VLLYRASVDAAFAVNSAHAKQKFRVIFSLFILMGVSWMMEVISFAVGGSAYIWIPTDILNILTGVFIFVIFVCKPNVWKLLKLKCPCLKRLD
GCCPSYMTRSNTRQGTTTRSTIKEVSHTKSLNNLGGGGGGGGGHQLDADSKKKIRMDAPTQSTQLRDSTQVDSGDEMMIDHESIRM
A
>Dpul_EFX89686
MRQHQLLAYYYAIAALCVTLRITFCAAGSDGETIKRDHQEEEEQQLIPLTKCCDLGQFYNAGFDRCLEWESYLAEVHLLATPFFQDVPP
FYNDNDGRVVHPVAPSAFLLSLANFTFCPSGQIARISPDFQLFKNGSMKTVEGKALKFGEFCVDRIISRELGASTNLTAGLFVTRFCIPD
PCAVNNNRTGCVRKCCPNGMALNGDERICQVNSAPFEIPFHDESGAALNASSHFGTSSSSSLVVSDGVFVDCQHGAYSLRPSVEGD
DEFYILPNGRIHTPALGEYHDDYCIDNFVSEDGVELQALMCFPPQAEESEENSVIQSIYPYFLFVSSLFLIATFVVHALLPELRNTHGVTV
MCHVASMTVMYVGLGTIQLSHHLSDGICVGLAVTVHFAFLATFTWLNVLSFDIWWTFNDRRLPRRTSKLGRRFAYYSLYAWSVPTVIV
VFGQVADNVKGLSKHIYQPEFGILKCWFHTPGSFYTFLYGPMALLILANILFFIMTATALCRTRVGTTRKDEANLFHSKQKFRAIFVLFIL
MGVCWITEVISFAVGGSAYLWIPTDILNILTAVFVFFIFVCKPSVWSLLMKKHPSLQKFDRLCPNCMKYDNGQQENEDRSFLQQTSI
>Tcas_Mthlsc
MLQCVLIIFLAVIFCVSCEDLCPKVLAVDITKGIKIHNSIIVHDGIKYEKSDYFVQNETIYGCVCNIKPCVRKCCGPNEIMVNKTCLESDHN
WSYPIHERTVARNNSSKHHFVYNKYCPRGRILLLPHLEASQRFYVQVNGSLFLPGFKNKMRNPENYCLEVFDIQRFNLKKMVSALVCL
TEEDTVAAESSNLLSVGMIISMPFLLLTFIAYALLPDRNLHMKALMFYVINLMFSYLLLVIIQLSESQFAEATCKSLGYFCLFFFLVSFFWM
NVICIDIWWGFSGLRGFTGSKKTAERKRFLFYCAYAWGMPVLHVLVVFIIDILANQNSVLKPDIGNGQCFLRRGFPELLYFYGPMAILITI
NIVLFAITAIKIRRIKSETAMLKKEDSKRHSYENDRQIFNLYVKLLFAMGVNWSMEIISWAVIWAEEAPEAIWYLTDFCNAIYGVFIFFIFVF
KRSIWKLLKKRFYWSKPKIVTMSTIS
>Agam_XP_316287.2

MHAARIVACLLVLFGVRSSVCSLPCDFIDSVNITDGERLPNDEIRHNGVIYNKTYYRVIDYDYQDFATKRYVPNYVRGCLCAVRICVRLC
CGEHEYLGKRCTHTDDYLPILVNVSATEAVDLRNHSLYGFLYGKPCEQVYELLPEESPADEWSIARNGSLIIADELLIQRNQYCLAPRQ
NNGFASGLVCFTTNEEFKYNLYPVGMLLSVPFLLLTFFVYACIPDLRNMHGKSLMCYVLGLSVGYTVLSMVQLRVFPGLSASCVISGYI
VYFSFMVSFFWLNVMSFDIYWTFKGVTGVRSSETKKFLLYSLYAWGCPILLVATAIIADYTDILPIYLRPQFGTTRCLFVENKLIEFLYLYM
PLLILVFMNVVFFVITALRIYKIQCETSVVRRGDSKRHTKLDNDRDRFGLYLRLFIVMGVTWSLEIISWAVDNNAWIFYVSDVCNCIQGFLI
FALFVLKQKIKRLIYKNTYQP
>Megachile_rotundata_XP_003703117.2Mthlike 3 isoform X2
MLTRIIWLVMIGWSSGIEGKRIDTIEESFCRPFPSKVLEGHKISRLDNGSLVHDRLVYPANSYRVVGNETYGCVCNIRLCLRKCCRRDEI
LGTSIRPNCTRLTNGQLAPDLKLEQRQLTTEIQGISGLSDLFVLVEDMQCPEGTGKFLLQPELYNDDAFVLLANGTLQMTTNKFAAWTY
CFDWKESFEKIVAIVCLSNFPPSKDEIRQKSYNIGVIVSIPFFFITFLVYAIIPELRNLYGKTLMCYVASLVVAYTFFILTTISSNLPFTICCTIA
FIIHFSFLASFFWLNVMCFDIWWTFGGFRSLQGSMIQRERKKFCIYSIYAWGCALLLTGVCILMDFLPNIPEHFVKPEFGVQSCWFNTNK
AKAIYFYGPMGVTVVCNICLFISTALKIVRHKKDTAHHLKGTDSRRHDDNKQWFNLYLKLFIVMGINWSMEIVSWLCNNSPAYI
WYLTDLTNTLQGVIIFLIFVWKDKIRRLLLKRLGCHGNNILSRNSTRSAYHSSTSRTTCTSAAPLQQKIIPYSSDPTSRNKTPFVDNDDSV
>Epiophlebia_superstes_GAVW01003911
SFAAPPPIEAHTTKPEDVATPCEIVMSVEIPSAVVYGNGSVWDPDGGTMYPPGTYWKVYGEGRVIKPVYRGCPCKLDRPCLRKCCKI
GQSMDKGGDCVPVKHPLNLNVTDNGGGDITQVDGQKHFFLLFGDPCSHGRYRLDPDEYEDDEFVLSSDGLLIQTMENMTDRGPKDF
CIEYFMDKETYLPLACFPPFNSTDSNTDLIYTMYPVGMLVSIPFLMCTFLVYALIPELRNLHGKSLMCHVSSLLTAYTFLAVVQLGGTQLK
DGICITSAFIIEFSFLATFFWLNIMCFDICLTFSGLRPLRGSLRERERKKFLIYSLYAWGCPLIILIVCIVMDYAPGIPDNLLKPRFGEMKCW
FKNKMDALAYFFGPIGVLVICNVVLFLITAIKIIQLKRETAMLKKGESRRHDDNEDRNRFNLYLKLLIVMGVNWVMELISWAVGGPEYLW
YLTDIGNTLQGFFIFLIFVWKDKI
>Cordulegaster_boltonii_GAYO01008982
EGEGEDKKSVYRGCPCNLDGRPCIRKCCPIGESMSNDGECVPKAYPFNLNVTDDGEYFFRVNGTKHFVLLFGNSCSYGRYLLDPFEY
ESDRFYIKASDGFLVQPYENYTEQGPKGFCIDYFPEKDAYLPLVCFPPVEDKEGNTDELIYTMYPVGMLVSIPFLLVTFLVYALIPELRNL
HGKSLMCHVSSLLTAYTFLAVVQLGGSKLSDGICVSSAFIIEFSFLATFFWLNIMCFDICLTFSGLRPLRGSLRERERKKFLIYSLYAWGC
PLIILIVCIVMDYAPGIPDDYLKPRFGERKCWFRHKMDAFAYFFGPIAVLVVCNVILFIITAVKIISLKRETSMLKKGESRRSDDNEDRHRF
NLYLKLLIVMGVNWVMELISWAIGGPGYLWYVTDIGNTLQGFFIFLIFVWKDKI
>Cordulegaster_boltonii_GAYO01127990
ITFCGMRPLPGSLKERENKKFIFYSLYAWGCPSIILIISLIMDFTPGIPDHYIKPRFGRTRCWFDRKMDAFAFFFGPVAMLITSNIIMFIITAV
KIVQLNRQTDMVKKAGGSCDDNENRNRFNLYLKLLIVMGVNWIMELISWAVGGPDYLWYVTDIGNTLQGVFIFVIFVWKDKI
>Cordulegaster_boltonii_GAYO01128358

LATFFVYALIPDLRNTHGKSLMCNVSCLIMGYVFVSLVQLSGKKLATNNCIASAFIIQFSFLAAFFWMNVMCIDIYLKFKDFKGHSTFTKE
LEKRRFIFYLAYAWGSPFLILLVTLIMDFAPGVPEYFIKPEFGKDRCWFKSKIATLTYFYGPVAVLLVCNIVLFIITAIRITRIKKETSILMDAE
NRRHNNLDDSNKQRFNLYLKLFIVMGINWMMEVISWAVGGPEYLWLLSDIGNTLQGFFIFIILVWKDRI
>Argulus_siamensis_JW948170_JW948387
PQFYDSLTGEPLKMAREDYQIIRYNDSLCESHQEYVIDSSEFYLIQTGELYAPATRRAFPLNRYCVERILSPDGRSIESDIAIVCMEKKTR
EWAYKDFCKDNICVQKCCPIGQAIYRMEACRDFPDPFIPNFFNNQGQPVAPPENYNITVHPPICAEGYPPLSFDPSREDDKFYVLPNG
NIYSLDQGITLAPPNYCIDMFFNESITAFSGSFCSELSMSDDVPPWVYILYPICMIISAMLLAITFLVYLFVPELHNLHGRTLMCYVASLFLA
YIILIHSQLQPDSLTHLTCKLTATIMHFAFMSSFFWLNVMCFDIWLTFSSMMTQTGSKAEREKRRFKRYCVYAWGCALILSTIVAVIDNLK
NLPPNIIRPRIGYQRCWIYGDMAIFVYFYGPVAVLLASNLVFFALTARQLIKATMETTMVHTTKKNKQRFLLYIKLFLVMGVVWTTEVISW
QVGPLEAWYVTDIANTLQGLIIFIIFVCKKKILRKVLNTAKKYYQDCRCCKSDTYRPATDNTPLRKVSN
>Argulus_siamensis_JW969407
PELEMVDNVPSWLYTLYPICMIISSMLLVLTFLVYLFVPELRNLHGRTLMCYVASLLLAYIALIHSQLKADKLTNITCKVIATIMHFAFLSTFF
WLNVMCFDIWLTFSSMMTQTGSKVEREKRRFKRYCLYAWGCPLIMSSIMAVMDNAKNIPKAIVRPRIGYQRCWIHGDMAIFVYFYGPI
AFLLVLNMVFFGLTAYQLVKVTMETTMIQTTKKNKQRFLLYIKLFLVMGIVWTTEVISWQAGPLEAWYVTDIA
NTLQGLIIFIIFVCKKKILKKVLKTAKTYYHECPCCKSKANQCATDQKPLRNLNETSSMQ
>Argulus_siamensis_JW965784
CRDGGPPIPFNLDNLQEELFVLPDGRIYSVEQGAYFSSPEYCIDTLFNQTHSSLFGLFCATYSHKDVDPVVATLHTLGMITSAIFLVLTLI
VYLIVPDLRNLHGRTLMCFVGTMLLAFILLIQEKLYKARRSNLTCKGLAYLLHFSLLSSFFWMNVMNFDICSMMTKGVKNERKNKRFKR
YCLYAWGVPLLMGAIVTALDNVKNLPYYIVRPRIGYELCWLHNGLAVFVYFYGPIAILLFFNILFFGVTVYQIFKANRDTTVVQNSAQTKK
RLFVYLKLFFLMGTVWIMEIVSWALQPTDYVWLVTDMVNILQGLFIFIIFVCKKK
>Dpul_EFX89687
MAVDVNDKACKSSDEPFNETVHRAFDLNPEMKIVDGYGFGMNCYDEASNLLELTEFHILSSGQMYAPDYPYDERATYQYCVDNFVSG
IQSPDQNQKSPMKGLRCFPQYFNLNEANQLAHSIYPYLLFISSGFLFLTFVVYAFLASEIFNAHGLIVMCYVASMAVLYACLGIFQLQLVV
PNLPVDPGYTCIGLAVVTHFFFLATFSWLHVMCFDIWRTFRKSTPKPFNRRVGASFIPYSIVGWGFPFLIVLTGQVLDRIVETRGNSSVI
QPLFGEGQTCWFNEWKSLFLYLYGPVAVIVLVNLSFFVMTAIQLCKTRKDSALALRNQQSQNHITRQRIRVIFSLFILMGVSWTMEVISF
AVGGSYLVWYIFDMLNIFTGVFVFIIFVCKKKVWKMLKKKFKYLRVNGLHILCPCCPSQQNNPALRASRTSKQSFCRHQSRSSVSTPSQ
LYFLKTSNDLKTKLSRKSVDEEDTCI
>Plutella_xylostella_XP_011552150.1_Mth2like isoform X3
MYIKLTLFLAVTSIAKCWTLSVSDGKVLCDRDTSVDISQGVKVDNAILYKGVRYGKGDYYFDSETQTHRGCICKKKTCVQKCCPFGFGY
DPGLKNCTPYTEVFDPPIWDEYHVLEGVHALQTFHLLFDVIECMKFNRSDMIRLRVSKLTENFHIRKDGKLHIESPEDVPPWTLRTPDK
YCVDTFVLEEEDGVSSRLDALVCFSTDAEDPHNYLLSAACMLISCVFILATLAVYAWLPELRNLYGKVLMAYLSCLLLAFLLLGSMQVLV

YVDNINTNMCLVLTFFIYFSMLSAFFWLNVMCFDIYWTFSGKRGLSLEKLSNRGRFLAYSTYAFGVPSVLTALLIGLEFSGMPVHPLMP
ALRHHGCFIHGNRKLLYLYGPILLLSLANIAFFTLTTIRIAGINRQTSVLQNKESATHDHQRKDKQRLLLYVKLFSVMGVSWILEVFSALYP
EAQLFWQFTDSYNALIGVSIFVVFVCKRKIFRPIKKRYNHLRGVPMARNQSSMGTCSSRDDVRMTVSLNHAT
>Dpul_EFX74305
MQYMAELDVCRDWKGSVTHSKMSAAPPVYSVERSAEPTPVRVESNSFQMTHRLKRCPKGYVGMSSTETKFRFYEDGSIFIQDEIVTN
YQNGDFCIQESFPSGQLMARYCVRDPCNHTDTCIRKCCPTFKAFDISTTRCINYSSQLNVTLYNRTGYSLNLKSEEMFVRDGVAPQCK
DGYKHRLLKQHDYHLRSNGLLFVFPKKRCSSQSQTDVDLITDEYCVDYFMDVDGTSAQRAMVCVAADDTNPIYSIFLFISSFFLVATFA
VYALIPEIRNIHGVVIMCYVVSRAAAYLIFGTLQLVGTTRSTVACRIMAILIHFSYISTYTWLNVVCFDIWWTLKSMQPCSSVNTAIRQNQL
GKPFFAYSLYSWGLAAVIVTVGQVLDYYKTSESVIRPEFGEDTCWFSSYGAMFAYVYGPVCVLIFVDIIFFVMTSILLRRAGIGSGNRHS
REKYRLRVIFGLFVLKIVSWLAEFVAYAIGCLNEAAFVIDVIETSSSVLTFAIFVCKPNVWRLFKQKCPCLERLERARPYSANYANRSSNQ
LTASTNISSREILHNDLIKTLSQRRESDKPV
>Dmel_Mthl1
MDRSRSSRAASSNQFIRPCGLLTTVILLQTLVSMSLAIEEMSPPPAAPPRPSPPPTVKLNKCCHSGEYLNDGTCIAGSEALWLPMVYLV
QQQRFFEPHGASPRFLKFLPNTRPTCRKDQTTEIFRSRGANVMLFPNGTLYVRERALMVQPSDYCVDWEVAVVCLNDSQPINALEDP
DYAANPLVQQEPPKASLRLSKCCGKWGSYNTQLQNCDLQPNHQAAVDGLLRLSPQLPEGSYQTSYGLPDCGQPGGYSIAGDWQDA
KLDRNTAMLQLPHKNLSAGQYCLEHTQREGEVKIIACQHLFSSAA
GAGIHDGSIGGTIEQANGQNLQKAVLTGGILVSIVFLSATLVAGFLLPAVHHALHWRCQICYVTCLLFGKILLAIEELSSSLQPGSAACHTL
AITMQFFFLAAFFWLNTMCFNIWWTFRDFRPSSLERNQEALRRYLYSLYAWGGPLLITFVAACVDQLPETTLLRPGFGQLYCWFDNRN
LSIFAYFYGPIGLLLCANIALFVSTTHQLTCGLWKRDDVKSSSEKSALGRVCLKLVVVMGVTWIADILSWLVGGPHGVWFFTDLINALQG
VFIFIVVGCQPQVWTACRRIFCPRLRHDITNTTNGVQHSSSSQGLPSMAGGTEITQNTTTTTTTTNTTATHMPSNPAEDEVPEKAPIAP
VAPIVKMETIC
>Dvir_Mthl1_XP_002059559.1
MNNYWLLLLLICAANGQQEEELVTGVAATPTATTTTTAPASASGRTPPPRVLLNKCCHQGEYLNGTTLECIAGSSEMWVPLVYLKQQQ
RFFEPKGAKPRFIKFQPNVRPNCQAQRQQQELFSSRQANVVLFPNGTMSVRERGLLVEPASYCVDQDVALVCLDSEPAPAAMDRER
APGSAPAGDTLQPAATTLAPLRLRKCCGKWGSYDSATKGCTLQTAAERADSGQLQLHLAPQLLPGSYQTSYGLPECLGNGSGYAIAG
DWHEATLNRSTGDLRLTHTNLSAVQYCLEHTERAGEVKIIACTQHFAGGHHNVPYGGVEGSLQKAVLDVGILISIVFLAATLVAGYMLP
AVHHALHWRCQIYYVFCLLLGKILLAIEELSTALVPGTVSCLLLAIGMQFFFLSAFFWLNTMCFNIWWTFRDFRPSSLERNQESLRLRLY
SAYAWGLPLLICAIAACVDQLPETETALLRPGFGQLYCWFDNRSLAIFAYFYGPIGLLLCANIVLFISTTHQLTCGLWKRDDVKSTTEKSA
LGRVCLKLVVVMGVTWIWDIISWLVGGPHDVWLLTDLINALQGVFIFIVVGCQPQVWTACKRICCPRLRHDITNTTNGVQHSSSSQGLP
SLANGTEFTQNTSMNNTTTTTTATAAVMSPASPVAEDAPQPAASPSGQVAAKIPMETIC
>Tcas_Mthl1

MPRRVLLLLLGAFSIVHSARNVTISKCCPFTATLNNENKCIPVLTPKWTLKVFSPRNRSYLPDNQVPPNWHLKPASKPPCAMPALLTPN
LGNYIPFQNGSLYVLEYDEVVHPDNFCIDYAAVLVCLKAQQPESMTAVRVKKCCGENAIFSETNKTCIHFKDAGYKIDVGSDKKLTAGF
PTCDHQEVVITEMLREAEMHKNGSLWLPKMKVLLPAANYCLENILEDAGHSPKIMICQEHVTVEKAESHDIRFIIYPISLALSAVFLAATL
AAGAILPASHHVLHWRCQTNHVACLLVGNVLLCITQLSGRMDATLCFSIAVSMHFLFLAAFFWLNTMCFNIWWTFRDLRPQSVEKSQE
WYRLRLYELYAWGVPVVIAGTAAILDQVADSSNFLRPKFGDNTCWFHGNLEKLTFFYGPIGTLLLINLALFALTARELTCGLWKRELVKS
TSERAALGRVCIKLVVVMGVSWIAEIISWMVGGPQELWYLTDLINCFQGVFIFIVVGCQPQVLSAVKRLWCLRKHRANGTAGTTNHHS
SSSQGMPSMGDTVTNHSVTNNTTKSVPLETSC
>Smar_012256
MPSLMIVLLLATTIAQDYDYYTDCENNNPYDTLEYCMHECQSIHLNKETFYTETTEPDYKIRYKVVHLTEFKFNSTGHLMSDLPKEISNN
SYVLDTDKQNVYVCYPLDEDFVNCSKWRLKNYEFHLHSNSRQLYNKPHKTFYPVGKFYLEELANNRMEAAVCYPMSEKQYKTNAIGT
MFLVAYAISALSLVLTFILSIVISELNMRNNYILLCHLVVMTLAYVLLFVRNVLPESTTSTGCLVLASLQQFSLLASFFWINVQAIDLFIRFIHL
RRNSDLRIRMRRFVGYCLYAWGCPLVICLVTLGMEKTDVQSFRPNFRLNCWFNQKDRLSMLVYLYGPIAVILFVNFVLFVLTVLKLYEI
GKTAGNVNKKMHNQLLQLYLKLVIVMGLFWVMEVIAWAVGGNRQNFYFTDVINALQGLFILLIYVCKPSYIRRIMYQNSFFPNRPKWWP
WQMNSQCKSEREVHECSEMHETLSATTSLTVNEPVPNNTTNL
>Dpul_EFX83097
MAEYTLVVILAFFVWFSNATVTSENQTVLEIRGNSSTRQEILFDEKIRCCKSGTTYLTGLDQCVPNYVSTLYNESFPNYSFEDATDGLTD
CPDEYVSHSTEHFKLDNDTLLVGGKRLESNEFCINRVEEENYSTSPGRFVARYCVPDPCGESGCIRKCCPPGMAMVMKYYHDLSPISI
CEPHPVPFNLSLLRNSKNESIDANSFSIRGGLGIKCGKLPRDQLLNFSMRPDGQIVLHLENQTDEKTDQYCIDNLVDDGNINVMAIVCFP
EQGEDAKGAYLFGCLQLFATVFLVATLLVYAILPKLRNLHGVVVMC
YLASMTATNVFHAILMLVRGCDMPPGLCSTLAISLHYAYIATFSWLNVLCFRVWRMFSSFKSPTNTSVLGKQFFYSSVYGWGIPFIVVV
VGQILQHSGGNTRLPPLLQHSDVPDDIIKPGLASKLYCWFNHKDRGPLIAYLYAPMMVMIVANVFMFVWAAFSVCQQRSAESSQTTSK
IQKFKVILSLFLLMGVPWTTEIITFMADASFESALVTDIFNIISPLFIFIILVCRPSVWKMSKLKIPCLNSFITACEKIVSRIIPIRKINRHQSASA
KERLCNSNTIGINNSYKTSQQTLTSESTDPKQIMELPSFFDVVVK
>Dpul_EFX83094
MAKHLLGVILAFFVWFSNATTTSENGTVLEIRGNSSTRQEILFDDEKIRCCKCWTTYLPGLDQCVPNYVSTLYNESFPNYSFEDATDGL
TDCPDGYVSHSTEDFQLDNDTLLIGDKRLESNEFCINRVEEENYSTSPGKFVARYCVPDPCSESGCIRKCCPPGMAMVTKYYHDLSPI
SVCEPHPVPFNLSLLRNSKNESIDANSFSIRGGLGIKCGKLPRDTIFDFSIRHDGQIVLQLEDQTDEKTDQYCIENNVVDEQGEDSKGAY
LFGCLQLFATVFLVATLLVYAILPKMRNLHGVIVMCYLASMTVTNVFHAIILMIVRVSDMPPGPCSTLAISLHFAYIASFSWLNVLCFGVW
RMFSSIRPPNNTSVLGKQFFYSSVYGWGIPFIVVVVGQILQHSDVPDDIIKPGLPSKLYCWFNHKHRGPLIAYLYAPMMVMIVANVFMF
VWAAFHFYQRSSESSQVTNVLHKGQRFKVILSLFLLMGIPWTAEIITFMTNASFESALVTDIFNIILPIFIFIIFVCKPSVWKMFKLKFPRLSP
FTSACEKIGSRIVPSKKRNRHQPASAEESTNNKNSQQTLMSESTDPKQIFELPLTRLP

>Dpul_EFX83013
MLSLTGTKFSAKQRQSGTTLVTDCRIKKETFKKQILFDEKIWCCKSGTTYLSGLDQCVPNYVSTLYNESFPNYSFEDATDGLTDCPDGY
VSHSTGDFKLNNGTILVNGKRFKQNEFCINRIEEENYSTSPGKFVARYCVPDPCGESGCIRKCCPPGMAMVTKYYHDLSPISICEPHPV
PLNLSLLRNPKNESIDANSFSIRGGLGIKCGKLPRDTIFDFSIRPDGQILMQLDNLPDEKTDQYCIDNQVDGGNINLVAMVCFPEQGEDA
KGAYLFGCLNLFATVFLVATLLVYAILPKLRNLHGVVVMCYLASMTVTNVFHAILMLVKGIDMPPGLCSTLGISLHFAYIASFSWLNVLCY
GVWRMFISIRSPTNTSVLGKQFFYSSLYGWGIPLVIVVVGQILQHSDVPDDIIKPGLPSKLYCWFNHKHRGPLIAYLYGPMIVMIVVNVF
MFVWAAFSFYQRSVESSQVTKVSRKKQRFKVILSLFLLMGVPWTTEIITFMANASFESALFTDIFNIILPIFIFIIFVCKPNVWKMMKLKFP
RTSPFISACEKLVSRILPNKKSQRQPIATADESQSFNSNNTTGSSSNKTSYQTEMTTMK
>Dpul_EFX83013.1
MLSLTGTKFSAKQRQSGTTLVTDCRIKKETFKKQILFDEKIWCCKSGTTYLSGLDQCVPNYVSTLYNESFPNYSFEDATDGLTDCPDGY
VSHSTGDFKLNNGTILVNGKRFKQNEFCINRIEEENYSTSPGKFVARYCVPDPCGESGCIRKCCPPGMAMVTKYYHDLSPISICEPHPV
PLNLSLLRNPKNESIDANSFSIRGGLGIKCGKLPRDTIFDFSIRPDGQILMQLDNLPDEKTDQYCIDNQVDGGNINLVAMVCFPEQGEDA
KGAYLFGCLNLFATVFLVATLLVYAILPKLRNLHGVVVMCYLASMTVTNVFHAILMLVKGIDMPPGLCSTLGISLHFAYIASFSWLNVLCY
GVWRMFISIRSPTNTSVLGKQFFYSSLYGWGIPLVIVVVGQILQHSDVPDDIIKPGLPSKLYCWFNHKHRGPLIAYLYGPMIVMIVVNVF
MFVWAAFSFYQRSVESSQVTKVSRKKQRFKVILSLFLLMGVPWTTEIITFMANASFESALFTDIFNIILPIFIFIIFVCKPNVWKMMKLKFP
RTSPFISACEKLVSRILPNKKSQRQPIATADESQSFNSNNTTGSSSNKTSYQTEMTTMK
>Ctel_216805
MRIYLAILMLVASGEAFRPSPFGRNSFQPDILPPELQEEQCGERESCFQNGRLNWSNTSELSGRNLFLRNCFCDPLCWAYGDCCSDV
TDKVTNRENPWNSSFPSKLDQDTFVCMFGSTKRLSVSDKLFVVVRCPVNWADETVRVNCEDPAPSDLFLNIPVIDHDGVVYQNKFCA
LCHEAGDTTFWQVSVECNMTLTNISSEELESILAEQLDAGSCFTQFQPPQLPIEFRPCKKLVNSCPDAWSDN
VTKELCENGEHRYVYEPSRMAMRPFKNEACAQCHNVSNAVCTEHRSFVLRSHHGIPMPYPLSFILDINTRSGDSTGYGISGRMSTDD
FNGTVGNTVTCKDNHVYDVFSQKCRSLGCPEGYTLIDEQCTPGANSTELACPMILLKSSEFAIIRGSDLYVNSSGEIHAFGTYLLDGKG
SAYICTNFSKDYLQEAIDLKFSSAQTVVSVIGQVISIVCLSVHLLVYSMFRKLRNLAGLNIMSLSGCLLIAQLLFLFAGSASASYSWCFSIG
VLVHYFFLASFLWMNVMAFDICRTFVKFSKPSARDDFLQKFLRYCVYCFLVALLIVLSAIVANFTLGDSLYRPAYGEDICWIKNRKALIVF
FAAPVAVIVTSNLVYFFITLWSIQKSMEASKMATNNKKGDRDRFWLYVKLSTIMGLTWVFGFVAAIFDVDVLWYLFIIFNSLQGAMICFAF
VMTKQVLSLLRGKFSKEPRSQTEMVTRSTAVSNSNTASGSASSHLLHKHR
>Ctel_208644
MSLRMYLAFLVLVACGEAFRTSHAGRKHNSSEPNIPQSERQEEECEERDSCFRNGRLNWSNTSELSSRNLFLRNCFCDSLCWAYGD
CCSDVIHGVNSRENPWSSPLPSKLDQDTFECASGATKNLTILDNLYLVVKCPVNWANETVRVNCEDSAPSDLLLNIPVTDDNGVVYQN
KFCALCHEAGDTTYWHVGVECDMALTNVSSEVLESVLTKQLDTGPCSTHFQPPLLPIKFRPCKKLVNFCPDTWSDPDACEHGEHRYV
YERRMTTMRPFKNPACAQCHNVSHPICTDYDSYVMRSEDGMPYPLSFMLDINTQSGESTGYRISGRMHTDDFDLTVGNTESCEENH

VYRVLSQKCRSLVCPEGYSLIDEQCTPGTNSPKLACPMIRLNSSEFTILHGSDLYVNDTGEIHAPGTYQLEGKGSAYICTKVLTSRLREA
IDPKFSSTQTIVSVIGQVISIVCLSLHLLVYSMFRKLRNLPGLNIMSLSACLLIAQLLFLFAGSVSVSYSWCFTIGVLVHYFFLASFLWMNV
MAFDICRTFVKFSKPSLRDDYSSKFIRYCAYCILVALVIVLSAIVANFTLGDSLYRPGYGEGICWIKNRKALLAFFAAPVAVIVTSNLVYFFI
TLWSIQKSMEASKMAAGNKKGERDRFWLYVKLSTIMGLTWVFGFVAAIFDVEVLWYLFIIFNSLQGAMICFAFVMTKQVLSLLRGKFSK
EPRSQTEMASSGVRSSSKELPSPEELSVFSGSSDERGFEVKGLRFIGFLAIVHIYSKSNQLILLMLNNLKLLGDQTLSDQDEVDADILYR
HLSQKAMLGKAIEYPS
>Ctel_63867
KFSVIQTAVSVIGQILSIIGLVIQLTVYSILPKLRNLAGLNLMSLSASLLVAQLLFLLGSSASSVYELCATMAAVTHFAFLASFSWMHVMAF
DIWKTFAKSSQPNSLDNSTKKYFRYSLYGWGVPLLITTTAITVNFSVAEDAAYRPAYGEMICWIRSRIGLAIFFATPVAAVLVFNIVFYTLT
VVSIYKITKMARGVTKPKSDQRQLWLYVKLTCIMGLTWIFGFVAAAFDLEVLWYLFIVVNSLQGAFICVAFVCTRNVVKLLREKFS
>Glycera_dibranchiata_GASB01015634
FPYCPQTKLDEHQYDLLPNGSLNVLVWNIIIDNSEYLMDNVSFQASVCLPVMPSPPALVEALRFDAAQGYITVVGNTVSVICLLVHLLVY
CSYSTLRNRPGKCIMCLSASLMFSQFLFMTGIGVVRPHELCYIIALCLQYGFLAYFFWMNVMAYDVWQTFMRVQSIQAGSEKSAQSCR
FLAYNLYAWGVPLVIVLVSLALDLSSSSIRPRYAESLCWFSDKEGLLYLFALPLSLILAVDLTFFLWTVISIYRASVVATFARNDGNQRKN
LALYVKLACIMGLTWITAFIAALTELNAIWYLFVILNSFQGVWILLAFVCTKKV
>Dpul_EFX73784.1
MLHPEDYEPIEVGQNVSICSIQPDSYFKYASTGQHYMSDICLAISVVCLAFHIIVHMILPKLRNLPGKNLLSLSCALFMAQLLFLTGIGLRD
TVGYRLCATLGVLTHWFYLAAFFWMNVMGFDICRTFTGSLIRHRGMQGRGQRCTFIFYSLYAWGFPTLVVSLGIMLDLTHLMDDYAPE
YGTRLCWISNKTGLGFLFMVPVAVLLLENMILFSLTVFSILKQRRAARFAVDKKQSYRKAGESKSIRFILYIKLGLIMGMGWIFGFIAALAK
IPALWYPFIFFNALQGAFIFVAFCCKRKIYFMLYQWATNRPHPSDSSSSSRTTASSNKPSTSTQRASVAVELPSNLAVEFESHPTNRFV
PIVQDSTQPVRQQSLSKHR
QY
>Dpul_EFX73786.1
SYAQRCLSNVCLTISVICLAFHIAIHIALPKLRNVPAKNLLALSCTLFLGQLLFLTGVGARNSIGYGPCALIAVLIHWSLMAAFFWMNIMGF
DMCRTFAGSQIKPRLQHASVTRRRTTFRSYSLYGWSCPSLIVVVALIVDFCGVTDSGWAPHYGTHQCWICNKNGLGLFFVLPMAVLLT
VNLVFFILTAWSIVKHLKMSDMIYQRNRKSYIVRLRTRFFLYVKLGTIMGLGWVSGFIAALADVPGLWYPFILFNTLQGTFIFLAFDCKRK
V
>Skow_XP_006818657
MSKACQSYSATVYHDLTGNMYKNPHCATCLGVRNDIHCGGEFDGARISGVGISFSMLLDFNSHTGLRLTSGDDIVVESHVTCDEHQV
YDPVANLCRQLSCSQGFELTDGACIQIMSAPVRLSDEQNRSEGFLQLVAFLESHIAKSDDFNIEHHMASIFTNILNIPMEYVTSPSLEVY
SNTEKHFLSTSSYSYVYSFFIPVSHINIASLAVSLEQNLRYENWYSDKMIAITYVSIGETNKDGNYTCTDSDHRIMTHTIQEIKETDKGTM

FYSNDTRIWYQDQNTHWQMYSTYRNVSWEHIVIVGVCESSHHQSQLIECTLVNLGSNYIEFVGEYNHSMYYSPTETIFGPGEFQIRNG
TLYVCNTFNQNGTMTSQRQVVFFQYSISQTILTAVGSCLSILFLLLSLATYCVFHSLRNIPGKTIMNLIIALLVGQSFLLFTSSHSDEFCIIIAI
TLHYSWLAAFFWMNVMAFDICQTFRRNVRLHGTNDRNALLYKYMMYAWGTPALIVLICIILNFSTSTLIGYGTGYTCWLADPVSLIVSFM
VPIAIIILFNLVYFLRTVFAIWSTSRVAQQATKKTKASNIRGQLLLYIKLSTIMGFTWIFGFIGAFTALPGFWYLFIILNSTQGVFIFFAFTCNK
RVVRMWQRKVRAKKRRVKQKRGQTGVTPSTSTSKTKSTEISMRVTSV
>Skow_XP_006821924.1
MKFKPVTSTVSCYTGRFFTYLIIFVASTDAGKLTQTSPNVETDTYTVSYPPVNGFGAIDSNVIELESRGSTQCSPFDSCGGRCGFRPKP
LSIWNHTEHTVINDAHLNCFCDTVCLVYGDCCYDYELMCNDEREELTTDDYIGGQLAGTTPTGITETEFPNNNDVINGFQCVVIREYSIN
PLWVYTRCAPNWQDSYIEQLCLTVQTTDSLSILPVQGSDGNTYRNVFCAQCNYINKVTFWVAMSTCSSPAPTSIATDPIVLSLYIRYRC
TTFLLGPSDSVPRICIRAIGHCDLKYSNTDISSKCSSKYNSIVIVAKETVLAVYKNQYCALCNGVLPSNIVCPVWKERDTPIGPLGTIEADD
GVVMLGENEYDHAKLPGSGTKTQAVSYAVLITLNFDGQESIRYDSETGSLVPVYSTCNPGKVYDPIMGQCREVYCANGYQLVGSECV
LVGTIPSVVIHTHPYNASMGFDSVSFTLTLECSLNFNVSCVTDVNQLRQEFHQFILNIFNITIGELVNVEVGEVEGHVEDIDTSNVNKTYK
ENGMLDECIISDLVFGGNVVFTVTITFYDVYWNLSDATFLQIFQKMSLVLMNEVSFEFQYLGNTVEVTIVNIDQNSTYQDPLLTMSYFSP
GCTNGNFTTYSYPDATFVRWNGTVALYVNTTESVYEWDEFLITSYTSSSETGHGDVEYTVMVCEKWPYIKDRDCPRVRLNSSQYEIF
PNRSIKYNDQIYDTDEYSIVNWEKGIIEICTPGYYDISPSFYDYPQFYTFLTVFLTLISLACLIGTFVTYAKFEELRTVPGLCIMSLTVSLFLA
QLLFLIGADKTEIYALCAIIAITLHYLNLSNFSWMNVLAYDAFKTFSVKSSKFKSYRSSKSQIVYYSIYGWGSPLLIVIPCVILNFCGCVDFL
FGYGYVVCWIINPYSLLFAFIIPIALILIVNTCFYISAIYHIRQTKQAVRRNSHRKRSDSDTAIYVKMSTVMGFAWIFGLIASFTDSGVFWVIF
IIMISLQGVFIFVAFVCNKRVRALYRKRHPRCCRCFTHRICCLRAERERKRRSTASDLPLAVIASPDADRGYQSNNDNFNGIVVDVDDD
VFV
>Spur_XP_011662057
MGSFVVNVKYAEPLTCMEDGEEQPFPDQTCPMRHYPYSCSIDQPACKCDDDCALFDDCCPDLREVYSNSTLSDAVTDKKAREENYL
PYMTCDDASAASSCAVNEAFDPFTRGCQVVYCLSGFIYNGTTETCERLPIDEVGGVCLSFYSQSSSQTEVNLTSSDVEYLAGAMNSLL
IGSPYDLLASTIVLELPQVSCNVSQLEFHDGERCYEQNETMECQACFDIELNFTALMASNLSLVSNTSFWFD
DTACYFTPSFGNVSVMKIDIDVPADWTCASYSSFFSGQTNDTLLPPFRVQVTRETGAYKMTALTGEYICRDHLILTCKGYLRADPGQFE
FTHDFEDAIRVGTGNTVIGQGKFQRMPNGSVQYCLEENSQSDRNLPKGLDIMTIVGTVLSIISVLAVITTYTVFPKLRNMAGKSVLALSV
ALLMVFLLMCLGVVSSKSTGLCKAVSAASHFFWLSIFFWMNALAIDLNRIFGSRAKIRIGSKSQIFYVWYSLYAWGTPALIVGACLTIDLC
ECTNLSFEYGSEGLCWLSSGDATLYGFGVPLAALLFLNTILFSDTVVGIRLTKKASEKALKERPALTRAKEELSLYIKLSCVMGFTWIFCF
VCEYSNIPELWYIFTAVNSLQGVFVLLAFGLNKRVTALWKEKLGIKRKEATSSQGDTKSTNTQNSTVNYNNTM
>Lgig_75877

SGKLNASLCLWLLLAQLLLMIHGFEDLVCRVIATICHFLWLAVFSQMSAIAVNMALTFYRITPIVSLDAAARFKRLTIVALTPPMVIVAVCT
GLDVGLTNGMLKVGYGTSLCSWISNDMETIVIFFYTPVCLSLVINFVCFVITLCGIEKSTKASSMVSHDTTRSERRKCVIYTKLSSIMGFT
WITGIIAAILEYPALWYIYIILNGLQGFFIFLSFICNRR
>Spur_XP_011660493
MALQKDHFVPWFHSVYFSLFFSFILPGGSPFRGAELYENYTVHGWTVPVCPRSLICNSCSTSYNSDGGCHCDPYCFYFNDCCIDYLN
ECETNLVDEFFHEATEGIGPSQFSCVQPAGNPRYDDKYWMVSQCDVRWSNEEIKAKCEGQAVDEDPLTLMPIQYLTSITFKNVYCAV
CNFKNISGITPWGIDATCLSEKYSNLSRSEFSAQQIGDIMGNCLIWNFESPFSPTAARQCQNPVIATCDVDPKNETMVLLAQALGYDFIN
GTCQRLLANPHCSFKEAIPILIEVECMYNDGDMSNCHPDVRLHDYVISLINGNYISSRNLTISRFQAEIQGNDSFTKCTQNLDIWGYLGIN
IDVSQENLDSVFLSTNQQSSAVNDICSNMTFRIYIGCSSINESCPNRVLSTYEFMKSHDNVTGQVFFVGETKYSLLESLYVIEYDITSSG
MNLKRESLEGCQVPSETCALVSQNISLFVDLGNGSYLYSPLNRTLSPNDYLDVGNDSIFVCSYVLVNEQSAAGFLRFTSAQSILSTIGIS
LSIIALIVTFVTYLQFNLLRKATSNTLIMSLCITLIFAQSLILFGGLATMNSNACLSFAILGHFMWIAVFSHTTVLAFDLHRRFGIATKFGLTDE
GAAVLSRFLLFSWGCPIFVVVPSLGIYFSGTKLPHFNLTYKTVTCWIGDGMANLYVFGIPIASLLVVNLILFVMVVAGLHRMRMSPVNKH
KSTDSNMSSDLLIYIKMSTLLGFTWVFGFVAAFANVTALWYIFILLNSLQGVYIFIAFICNKRVFKLWRDSCTCLDRVSSTSSSKSRATSS
DVVTMKKSDWKTTTSESML
>Spur_gi_XP_011675990
MNVSSGNSEIGTCLQVVILSFDLSRNFVPFKQPSENVLWASSGIKNAKNLTCRISEVDISVGCVKREDRLCLNNSIRFHEFKVAGDSSR
MTFFVSETNTSYAIEDSLYTQTFRWNTDNSSNLDADLLVCRRSEYPSQCALITLDISDFVIWSNGSVLYEPYNMVFDTDDYILVNNDSIQ
VCSFLDGSGTLIREYYFIQFSKGQSITSFVGCLLSIAGLVFTFVTYAKFGSLRKSICSQVVMSICVSLTLAQLTLLFSGMAKSNSISCTCFA
VLGHYLWLVVFTHSVALALDLYRRFGITQKVKKTSEGPKLLSMFLIFSWGSPLLIVIPCLVVHLCKLPHVALTYGTAKSCWIGNGFMNLY
AFGVPIALTLVVNGTLFVLVVAGLRKRTNQTTKRKTSKMVTEGVVYLKMSTLLGFTWIFGFIAAFVDVEALWYIFIILNSLQGVYIFIAFICN
KRVFKLWRDSCSCKPCVSLTVSCPCLRSSSDESSSSIKTASSTWTSSFLSSTKKGASSTSISMTEASSSYSVPEKNQNQIETDSSALK
GPIPVLPSSSPSECTDRPVNVQVSSQLMSELKMKFDRKVNIELFVHKA
>Spur_gi_XP_798567
MTFFVSETNTSYAIEDSLYTQRFRWNTDNSSNLDADLLVCRRSEYPSQCALITLDISDFVIWSNGSVLYEPYNMVFDTDDYVLVNNDSI
QVCSFLDGNGNRTREYYFIQFSKGQSITSFVGCLLSIAGLVFTFVTYAKFGSLRKSICSQLVMSICASLILAQFTLLFSGMAKSNSFSCTC
FAVLGHYLWLVVFTHSVALALDLYRRFGLTQKVKKTSEGPKLLSMFLVFAWGSPLLIVIPCLVVHLCKLPHVAL
TYGIVKSCWIGNGFMNLYAFGVPIALTLVVNGTLFLLVVAGLRKRTNQTTKRKTSKTATEGVVYLKMSTLLGFTWIFGFIAAFVDVTALW
YIFIILNSLQGVYIFIAFICNKRVFKLWRDSCSCKPCVSLTVSCPCLRSSVGY
>Spur_XP_003723996
MKLRAISKKCLLMTMTMATMMDIVIGYTTFRPMTFLPPTPPLTFLPPTPPPTQFFTPGPFTAPPRPPTTTELRRPTQGVAAPFDLNPYRT
TTTPPYSPRTKLVIECPHGFEHILTPCRLPENDPPWPECHVTTLQVMHRFSMDGYYSCLNEVFTRTTRDYLGPSVNGNETTALKYPVA

TVEWWAMINVTNARLLQNDIENVLQDELNNLWSSQCADFIEFGVTCSLEGDRNDSISTPLSSRECDEELFFGQPDAFTPVWQNGSFLL
FFNGSYVLPTWWNDRTTYDVVESYLYDSSRQDKTREFRVCGARKAMINCAVVLPEEKYSLKDVGNSTVVVYNETEYEDELFEYVSNT
SIRICVTHNNFSTVLEQEGEVEEVPTTKTVNVQLIYSHVLLAISTLCLILLIVTYLMFPELRNFQGCAILSFALSLLISQICLQYVAPYARRTT
ALCQGVAVLAHFTLLCAFAWMTLLAFDLCRSFRVTQTRSHRDPTSRLKRYARHSLVGWGVPLLLVIVALGLHFTENDVLPLEYGSGRN
CWVYPVLANIVFFIGPVVLSLVANVILFTITVVNIWRTRKMTRGALQQDRSHKHVISELLIYFKISCLMGFSWLFGLIAALGTTSALWYLFIT
VNGLQGISVFLSFGVNARVRKLWSKKMTSAGSSTSSGSSNKTKKTSSV
>Nvec_NVE24038_XP_001640473_SB_36244
MHTDAMKIQLLLLSSAILSLQASASKIKQTWSKGKEIRNGGKPIEKVLENLNLKTLVKQNYPGKYEWKYEILIKRVQITKNETGASGRSSS
REKRSGQRVSETSSKIADPRKSDCHRDLSCKGKCKSSPSHLLKKEFVCYCDEVCERFVDCCADYEHFCKLNATGNFVLTPSSDNLTT
VGEYKRDEKSWGEWRCIQWKITKLGGYPSVWVIDKCVGNHVDSLALSKCSTTPEISEVTSVQELLDARPVYGDDGKVYKNRYCAMC
NGLDERKVKPYGISLICDVKPPDTMSTEQKLDYLVTYCREGFAWEIPSESPRRYCLKLESCSDPCGKKCLNAQVGVVASGDVRNLRN
YRNMYCNKEQFMICGPVSTGLPWEMPPTKSFSVIFDFKTNNGLPTINSKPDCKSDEVFDPYMETCSKRSVFLQTPPKVSSADKYRIVL
WLSFCELDYQHADFANALGKSFGDEWDFTNIEATQSGSRATSDSKQWTLTFEAELMTQVDRSRRQSTGTADFDRVLHFTTPINITINH
TECVVFKASKRILSCSDLRQYNKSEYTQLVTPTGAVYVNISDEVIHARDYYTNGTDASVGPIYVCYSSRVDLLNCSGVFFPVKHFVIFQN
RSIFVNKSGGTYNASEYFVDNGTTHVCTEVRPRGEKIPDDTALAWLTFFCMLLSIATLVLFLVTYSLFSELRTIPGVNLMNLAVSILLAQV
TWLTGVNQTDRTLACTVVAAMLQYFYLVSFMWTSIIAVDTWRAFSRKSHRGIPRSRREKCVRIGWLSALGWLPLMIFVLICVSLDQSGT
VAIGYGSSTSCWMSNGNATLYVFAIPIALALVVNAIFYILTVNAIKAATQQARMAAENSQTRRHFGIYVRLAAVMGFTWVFGFGAVLLWS
PLWYVFVSLNCLQGVYLAAAFALSDQARKHYRELLGIHKSQKTSQGRGGSYAQSVSVESRLERAGTSSESCM
>Nvec_NVE15181_XP_001636770_SB_45587
MQPSIRRRRSIQERTQAREDSYRTWEYWSCERTADKTFVWMIIGCPSAFTDITIKSRCVSGFSLTKVESAQALLNARPVYTTDGKVFK
NRYCAECHGLSVPSLKVYGVSFECNFTAPESMSNEEKLDFLVSRCGEKISWVIPRGSPRRTCFKVASCSNNTDDCDARCTNGTVGIV
SSANGNYRNSFCYSESSLSCQPKIQTENGGPPLLPPFSIIFNFGTNDRSTPLTSIIECTKGHVYNPYTEVCASDFHSSPPESSTNDKYRV
TLWIRINDVDDSTCDINIVLALYKTFPGPWKFTNIETKEEHSYLNVYFDVEATYKNRRNRRDAMVTISLDRVLHFTSPFNMTICGTEYEVF
KATKRILSCSNLRKYEKSEYATFVSPTASVYINESGEVLRARDYYTNGTNVTIGPIYVCHAGRHEVSNCSGVFLKVEHFIIFPNRSIYVNV
SGRMLNQSEYFVHNGTTYVCTDLPPRREKTWADDPALSWLTLMCMLLSIAGLVFFLVTYLLFSELRTIPGVNLMNLALSTLLAQVTWLT
GVNQTDTPITCTVVAALLQYFYLVSFTWTSIIAYDTWRAFSSKSYRAALGSSSEKLRKSLKYIALGWLPLSIFVITCVSLDQSEAVTIVYGS
QQ
FCWITNGLATVYVFAIPIALALITDVIFYALTIRAIKTTASQAQMAAENSKRRGHFGIYVRLASVMGFTWVFGFGAALLWSPLWYVFVSLN
CLQGVYLAAAFALSDRARKLYCELLGLQATHVKTGTTKTREECENKITTSAT
>Nvec_NVE5678_XP_001628572_SB_39597

MNPRSLSLAFFVVFAIRLAYVRSQQEPKDGGVEGLGVLNGKRSSEIRKRMKYDQESREIYRGKGTENNQLEDLYDVKEKSLKPVARD
HYNKIPNQRDFDTAKDPKSTKRFSNSLKTWESKAFGNIRESELEMFGPGVKHVKLIKQSRKHVVQHQRLDIPSRRQKRSEMSILNESS
TTPHPDEPPPSPTNNNEDAPQKLFCTKDATSCKNRCNNTRMFSTEAQLMCSCDLQCVYFYDCCADYHEYCSSYITYQGDISSPIPDF
GGVLPDLGDRPVEEEMPPPWGNWTCILPGMNPLAVAKGAAGVWMVGHCPRSADQDTSSKCAVYATDGFPVTRANVKDVLPVFTPN
GIVYRNPYCALCNGVSTADTKSYTLSFNCENQPPTHLGAEKRMDFLFEYCRSAMSWNPGVGQPRRYCFRVQQACGFYAPQAAKDK
CKNSPPAIVYYNFRNYKNIDCVNCGRHNTSIGCGPGTKDKPGAYVPLVAPFNLVFNFGGKEGPMDVTTQVTCPLRSYYDPYLEICKEG
LSPPPRASGNDEYRVLLWLRSLGFLNTSPDNLERELPLTLSQAFGMKMGDVTNVGFTDMQENQYKLQFDASVERKSARNKRSFYQL
FCDQILNFDAPFNLSINGTTFNTFKATYRILGCFNLHSYQRQEYTTLNDTAVYLIDAGAVIQAKDYYHNGSSGEILVCMDGRTRNCTGTY
VRLSENEYTMISNRSVYVNRTKEIFSHMEYVTRDGSLWICRNYSNAYTIRSEKGSEVLRMVTFVCLLISVIALGFVLVTYSLFSELRTTPG
KNLMNLSTAILLSQIFWLSGSGQVHDRTACTVVAILLHYFFLASFIWTAIIAWDTWRAFSHRSHRSAKTMKRENLIGTLRCMAVGWLPAL
LFVAITTAIDLSGAVAIGYGSDVACWITNGTPLLVVFGLPVVVILVFNSIFYALTIIAIRATAKQARFASSDSRQKAHFWIYLRIAAIMGFTWV
FGFIAAVGITALWYVFVILNCLQGVYIALAFALTERARNLYKAVVITWRMKLRDDKSVKTSTQLTMDSPMAQHNHTIDTRL
>Dpul_EFX67288
FILYSMYAWLVPGLIVAAAVAVEYVDTDLFSWMPAEYRPFFGRNVCWFGQRKAILIYFAAPLTIILSVNLLLFISSAHMIRSTTSKSPASSN
QAGTRKQLGLYVRLALIMGLSWLAGLIAGAADFMPLWYVFVALCSLQGVFILLAYTCNPKVIRSIRNKICGCRKPKGRQSTYRAGPAGG
LKSEDLRRQGKANVCDSQDSNTSHLSRTSLSTKTTSTTSP
>Dpul_EFX79407.1
MSKKRKILSSISVFSFVFLVLLPDDCTSSLATKLDLIHRELASVNPAWTRVLNYILDSNQFNTNKDLGQQTTTSQDNQECCLTDHSGWW
CRRRRQRHHHEGHLNPNHVDNNNSNSNLPSDQLGHRSTRPADLYEATDWHCPPAAAVVPTNRLMAEVVYSGEVDSSSRHHVELPA
IFSVLINFGNSEYYQFLISMLDYLEEDHGCSSGYVWDPFASACRRIYCSPGHVHGFQEDFCDYIDANETDTDWRMTYVMELEVIQLTLY
VDAIYDSRNISDDDLLAIIQDSFAPVFAAFVGIDPGRITNLDVAFVNRTPKENKPAAPMMDQQRSLSIDFWLSQAEDGSDEPTIDSVVAL
MGSLIVQDRLIAVIDGVVCQLVGLHEQPVPSETDTFANWCRGGITAIYHNDEFVIDVTSNNTETNQSELIVYIKATDQWYKSGEYMADLF
FYGSSNSSGLTSLSGSVAVCENRSVIEDTSCNVIHLNQTEFVFFDNNTVLYNGSIPFIDPTLQPGEYESLPDGGLAVCLYISQKWPVALI
VESYLTLCLMSVSILAMMATIVTYLIFPELRNLAGLAVMNLCLMTSGFQLCIMIGMSLSVHQKSELCVASAILVHYEGLASIFWTNVMAIDL
FLTLGRWSAAPRKPSKILPRYSLYAYGLPLAIVSVAVAINFCDCTGEFEVEYGKLFCWISNPTANMVFFGLPLALALVANIVLFVRTVAIIY
SSSACQGKQCSRRQRALCQLKLYARMSTVMGFAWIFAFIAACFDPSSTAGMIFTFVYVILNSMGGLFIFVAFTCNRRVYLLYRGWWAR
RRNQLLRRSSTTTADSLSSTKTKAAPSVSHQSRQAKTISVETLVSNSDAGDVSQNRETSLHM
>Dmel_Mthl15
MNIGLSLVCLILSGISKVLAVQLCCAANSLLFKYRRTVDNAEIYQCASAASNISELPDLAQVIGK
LIPPMGLGLEKAINDSGHSQFALSKCQNFSSLSISDLGERTLNLRNNSCIGLLNRRLIVLSCEFEKNVPSQSVGFVNKCCPQGFIYVSEN
NTCKPGESDFFVYTSIISSPIIFFDNTLNCSDHRALVEYTLSSGKVHFHNNSLIWKDGTNSLPSSKFCIEAIHDSDDADLDRGALPEQKFL

VRACQEMKICQKIPCIRRCCAEGEMYAKGNFSTYCKIDGTDFKFEGFQNLNINANFSKPSDFGIVHGLQCPKFRLDPDNFPDDSHTINP
SNGSLIIHNTFKTYTNTQYCVERVRPNQKLYTFLCFDNKVVTGDRIRFKMYPIGLLISCCFYALTLIVYISIAKLRNLPGKILICLVSSLFAAY
LGIALGQLRPTSNDDICFLSGFFVYFCLMAAFSWMNITSFDIWKTFGSTKLKSCEKSDLRRQFIWYSCYGWGLPTLLTGITIAFTKSDILP
DAVRPNFGHGRCWFTYDSFGSASLLFFSGPVGILFIINLVLFVLTMKYCNKVKNEIYKMQSLNSDKPVLKRRFFQDKTRFVMNTKLCFV
MGITWLLEIVSILFYDHKKTFFWTISDSFNVLLGIFVFIIFVFKRRIYNEIMFKFGLQSNPSTASTRTRAGVTKSYAPTSTAMTTLRHSPGS
CEFKRQTSTRPENEVML
>Dvir_XP_002060099.1
MCVKLVLMYLIFSYLVTNNNSIQICCTPQSILYKSQGSNNSTYLKCIHAMDGLNETVNPTSMDMLGKTESPQGYGLEILKEISRHMKLLQ
CAHTSILNISLFTEISVNVSPESCLLIVNNSLIVVTCKSSIGDELQSIGFVNKCCPHGYTYVSELNKCYLREPDFNVYSSIFDKPMIFVDNSF
VCPKNNVLVEYVVEAKTIHLKKRQILLKDYGRSFKRNEFCIEAIEVLIENRNPQLYKNNEQFLVRTCQQLQICDSVPCIRKCCADGEIYYK
GNMTTSCIGDENNSKFQSLATMNLSGVFTKPSGMXYPVGLLISCCFYAITLLVYTSIEKLRNLPGKILICLVSSLLFAYLGIALGQLFPTKN
DDMCFLSGXNIVFFKGFLVYFFLMAAFSWMNITCFDIWKTFGXYRSTKSTNLQISEQRTLFLWYSFYGWGMPTVLTIITASLSKSDVLNE
TIRPMFGHGRCWFREIFRMQSSNTEKHTLRSRFFMDKTRFIMNTKLCFVMGITWLLEIASILFYDHKKNFFWSISDSFNVLLGVFVFIIFV
FKKRVWKEIMVKLG
>Cgig_10021040
MDQKEFRTLANNPDIRSLPFHYRQDDEIYRICVADILPLFNTGGIAQGPRILLWSITLGVNVMSIVSLLLTISIYIKVKTLQTLAGKNNMILFV
SLTITLSIHQLSQVGTFSDLACAVFGVSLHYFWLLSFFCMTICSYHMNKVFHQNNLVTMRQKSAIFLKYLSVVIIGPAIIVSANIIGTFAIQM
DGTLGYGQIRCFIDNRHSLLASFVVPVAFLCSANTIFFIRTLISIKKTPDVPENTKKRNEFTIFVRLFTVTGLTWILQIVDAFLQFSAFSFAA
EVINGLQGVAIFMAFITNNRVIHLLKREFEERAIPDRLYAVKK
>Cgig_10015721
MYEDPVGVCLVDQSLINIDPVLCEQGDLELEKYPFKNFYCEVCGTYPGSINPLPTYPPIQDPTYRQLFSVLDTPTSNFYFNTENCQENE
VLDVYKGKCRKLYCTKGKFVKNSTCVHFIPFVKSVNYRMAFKVSLVTNGTDAYSALQNLQMVFQHDFQDLNVYLEFIEFYAFVDQPCS
VLQIEPQNKEEATVYLKLMMRQSSLLNKTAIEEKLLRFKRLTVDYNGVSYTFQESVNAWYFPVLYLSDELNEGCLIKETLYYGTELFFDV
NDLLLCTQIELEPDEFEFSEDMTRVYIPSVDIYFNINEFHITHNGTIRICSESYKRIAKPKPASVMRTILIVVTYISTILSLLGLTLTFLTFCILP
SLRTLPGKNMMCYTLSLFAAQLLFLVRSHSELLDSSACAIMGGLTHYFWLTVFTCTNIVSFHMFRLFVFSSLIHDDKNNVCQFLKNVFIAI
FTPTVPVVINIILSYTVNGTQFGYGGSACFLPSSIALLWFVILPITLQILFNLLMFSITFHYIRKTPKVESSKDRNEFSIFLKLFLLTGVSWILL
VVDGFFEISLFTFLATIINGSQGVFLFISYVCNKKVLRALKQKFVGKSEGTTFTGNTSLISRSGGASFNSQQHGSDTTRTSKI
>Cgig_10001367
MWVTYLSTILSMFALFVTFVIYCILPALRTIPGKNLMCFAFSLFFAQLFFIIRSHVTDDTACAWIGGFTHYFWISVFTCTNVCCFHMFKVFV
RNSLVHANTSADRKLFTTYSFISLILPMIPVAINLTLFEIGYVSFGYGGSKCFLVSHLALLATFILPIVLQIIFNIVMFSITFHFIKNTPKVQGS
QERNELSIFLKLFLLTGVSWLFMIVDGFFKVSPFTFLATIISGSLGVFIFISYVCHKKVFKLLKRKLCCKVVYVSSS

TNICSATNSPSQITMKSKHTENEDTLI
>Cgig_10006654
MESVQNSWFGVVLIGFLQHRLCWADLSDPRDFYQQYVSVCGFGYLCNKGLENSASFEDVPTKIGPCPPCACDDECFTRNDCCPDVL
YEDKVWTQCWSTDIFTFPGLESNMYPLIKQCPTEAVKEDHLLCSESTDFSFPPIYRYRYVPVSSNRTRFSYKNIYCALCNGENARDLEK
WEISTSKEVCSKTSYLRQISFPYDMLADYKAPKLCPAWHVPKWPTKVPRCESSERVRFTRCNVTGTWQKKAYSVDIERACLSGYGPV
FRLYSNIFCFICNPPYTDALLLPLCRRDIRETSAGAQIEHLCKNYESSAVTYPYKNVFCYLCNLINLNGVFRKSFTVSEHHEFLYQGSVLY
TYENVALSHTQDLYQQIIRHSRITDAEISDYDPDSLILNGRGFNLSSVLLTKILSTPTKICNKNLLPKSVQNIVSQDCSCDPSCFFRNKCSC
CVDVALSYPLGCFDNSFVVTNGCYGNKTGNLPYFSTIKSLCEDFPQLDKVPVHSDGIDYQNLFCFLCNSNYDIVNNTLMMFSKYVMRG
FMFRCRDVIPISYSVSVANLFKHAHDSNCIITFDGKTPQSRACATQYFSTCDGFPTSNPDLSEICHLTSYDNFPSYAYYKNEFCYLCSRE
QFIENPKENCSGNNASLELEEACRDLPLSYSPNVHPYKNSFCKMCDSKCLPDCDFGFENRDLDECFGKVTAIGIIDPPSLRGIFGMSTR
ESDDSKPIQNHQVNETLFFDEANNIYRPISCYSGRYLNNGSCVLPSKKNKAGEYAIFAESTGKVSGDLGNGWNILNALKKIFIKELSVFE
RREFYTLRVLKMFANISCVDDLPNDVWTGFRISTKIVVSPGDVIFTDISRLENKLFQIVDIIQSKSRIIFQNFYIENMTTNEINIFKYMKSNID
FSMVSKNHCEYSLENTRLSIHSPVTVLANILKCKHIQFGPEEYIMDSSGIATVLSPAVKLLPGEYVTLASNSISFCIDVYLASYNPGTFGNL
SKKDEILRWVTLSTTLLSLICLFMTFIVYCTISSLQTLPGKNLMVLVLNLFLAQFFFIMGNNMTENNLVCVVLGILQHFLWLSMLCSFTVYS
FHTFKVFHTLNSRPTFTAQLFRKYLAITYLIPTIVVAITITVSFIYFGNSGYGKEVCFLSNLNFRIGVFLVPIILSVIVNLSLLLWTMGKIVSTK
QIKSSNHERSNFTIFLKLFALSGGLWGLQIVDGFFNISVFSYLISILNCLQGVFIFVSFVLNKRTIQLIVSFKKQEGLGEESTTNNYTGDER
QQNTDSTRTTRI
>Cgig_10025895
MVVPGLRTLPGLNTMSTTFSLLCMQLTYMFANAVEKSSLFCTVIGILLHYFWLSLCCCLFTCSLHMCRSFSDRRGYSHIHTNIKFIFGRY
VIYCYITPIFIITTNIILTYYIQNKIGYGTTLCFVEDFVQNLATFIAPLLITCVVNIVMFTITVTNINSVKHIEKSKKDKSELVIFLKLFSLTGIVWIF
QIVDGVLNISAFSFVATLLTSSQGTIIFLSFASSSRILNYLRSKRENKGKKEINYSKQTTRKGSGTSPLDLTKSTQAQGSSITI
>Spur_XP_011679633.1
MSAEDVKNTLDIGTEFGTYIEDLTIVSPNYPDNYPNNVNTQWLVSGPADYQIMAVFSTFNLEDSYDSLRIGSGLDPDDTASLLATLSGSI
LPDIIVSNSNEMWLVFTSDVSVTELGFRVNIEVYTPGNYADYTDFSLSSDFLDMSSIDSKDFMTDSSSSPDFPDISIFYSSDSVINASAEN
MDLNQYADNTCFEESCLDGQETLCRCDDMCQLFGDCCPDYPIPISSGTLSNEILSNETLSNETLSNETLSNETLSNETLSNETLSNETL
SNETVLDIDVWECIVDSFLPLFYPFLGHGYRMIARCPSRWSLEEDVKRNCEEVSPNATIDVLYIYQDTYFRNLDCATCHGYTESSLTAV
VISADQKCWWGYNNTLACMTPMPTLQSNVSMNISRSDIMPSVQTIQKPRLCLVNTTGSCPNGTNEEIARECQFYYKPCISEGIVSKHP
ACSYCNGGSMGISSPSGSDDSLHYDYYDSSMLDGADGLVDIFQMLNAPIAAPIQTFCLEGFELDGNGYCAPLSSDWPVCQSRHFGVK
QSKGTVECLEEMVCFREVTVDNITTSAKVPPPCLVRSSTDEINDIITFNLLYRDDMNPSLYTIQSDIINRIKNPSRDGCNDIVISASQFCHV
EDNPGNITYRCPEEMFVGYCLDFTPFLTNNESLIFFNGTYIRPKWWSNITNMQFSSDNVSSEYQFVMCGTEIALQTCSFFTVSNDSIEW

DYQGNQSVILYQGQMYDTEDILIHPDGSISICYSDQVTPIDSRTLMIIGHVLFAISSVCLFATLITYVAFSTLRNRQGVSIMNFIVALFLGQIF
LQYMRLSVSQFRI
PCIVVAALSHFFFLASFVWTTILAWDLSRSFAANKIKSFSSSSFGRKMVAFLVIGWGTPLVFVSITLVLQFGRFQNGPFLEYDSNTCWLT
KLSSIVMFFVPMSVCLLSNLVFFIITVHGVHSTKKATSVLKSGQDSHFKQLAVELRIYVKISALLGFAWLFGFLAMVTNVPFMEYLFIIVISL
QGVFIFISFGTNIRVKKLWRTFVENSSLLSMSKFSRGSTGAHDGPKSEKH
>Bbel_293770F
MNNYNYRRVELTVTREVKDLKQRLDQQRKEEERALAKRCKDRNELARLKRETQQKHIQVAVQHRQQLDEIHSNRTEELKKNHKEIEE
AISKEEEKANAEIMTEYEKKSKDLPETWRALQQERDSASGSINGPSTPTGTGTSLPILNFTSDEFQILPNGSVRLLSSNVSCPAEQVVIL
NTSASICGDCLLEYFSKDARQATWDANKSWLTLGLVTVSVVAVTGFVFHTYRSGQWKKVPEKLKVQMMTCMAASEVLFVARGFIPVG
PVCTAFAVLLHYSFLTAFSSMNVLAVDLFLTFRQESERAEPYKYVLYTWLVPVPIVLATVIVEFGSSVRVGYGEHCWIGNPTASLVAFGI
PVLCALMINGFFTIFALLAIRKSFQIASTALERSEISKIWVYMRISFLTGFTWIFGFIFPFANSEVLEYIFIVLNASQGLVMTLMLTMTGEVVG
KCKSAIMARLGLAAAEQDNGRTATASHLQTTGGKTEVTSAGGTGSAIEMTIMADVEENRARHRQDKGRNATASNKPTTAGPGETDTA
GNLQTLAGGVGETASGTEVATDEIPMKTFVHVEENKARDKRSACASDRDENETSFNSYHLSCP
>Bbel_192410R
MGNLSTTGTSLVLLWSLCYCLLGAFAISPDINVAVGKTAFQTSTHNGTDGVASLAIDGSTNTYMTGGNLTCTLTQEETDPSWWVDLGR
SYVIDRVVIINRMDCCSERLNPFNIHIGDSDQVSENPKCGGDHQIDMTTPAISIPCRWMAGRYVGVRLTGPSRILTLCEVQVITVGECSN
GYWHLPHTRRCYRAYDTESEYDQALATCREEGNTLAMPGDSATNDFLVALKNAADTTKVFYFGMTRSDDGTWKYARGSQPMEWEN
WAEGEPTGGKQRCAAYFPDNSSDVPYDKRNKWNDVGCKTDAAFICEFEYCQVGDGASYRGTVSVTNTGKTCQRWDRLFPHVHDKT
PNNYPSSGLEENYCRNPDGESGVWCYTTNALQRWELCDVPVCAPKKWREDLRCGEDYPAENHYPAECDPGSNYPCCSSENRCGS
TAAECDCMYCVNYRDTLSGICANGNVIDKTQICDDRNDCGDNTDEHCWICANGDITHKTQVCDGRGDCGDNSDEQHCWICANGNIIH
KDQECDGKDLGNGTDDCGDNSDEQLCCERRDRKSCANGQCIEKDGFCDRFTRCADGSDELYCPPSACDNGGLFHPFFRCDRRDD
CGDNSDEKNCDCYYLRDNGASYRGSENQQHSCQFWTSQYPHAHNHTPEAYPSAGLEQNYCRNPDGKDRPWCYTNNPLVRWSYC
DDVFVCDAAPTSCFFTYDKGRSYAGNINRAGDRVCQRWDSQSPHPHPHTPQAHPDAGLEENFCRNPDNKERPWCYTTDESLTWDY
CNVAECDDGMRTLRLRRQDCILYVDNTMLSYVELVLTLCSKRCKTPHVFEFFLFILQGKMREVCNFTEAVRTPSDRDQEKWKCLSGY
NNARSYWLVADCPDDWTDDVTTDQCLKEADPYNPGDLVYRMPVYDTSTKISYKNIFCAFCNNNCSKPILNFTSEEFQILPNGSVHLLS
SNMTCPAEQVAILNTSASICGDCLLEYFSNDTLQTAWDATKSWLTLGLVSVSDIAVAGFVFYTYKSGQWKKVPEKLKVQVMTCMAVAV
TQFVARAFIPPGPVCTVYAILLHYFMLTAFTSMNVLAVDLFLTFREDSERAELYKYILYTWLVPVPIVLATVIVEFGSSVRVGYGEHCWIG
NPTASLVAFGVPVFCALLINGVFTILVLQALQKSFQIANAALPRSEISKIWVYIRVSFLAGFTWILGFIFPFVKSEVLEYIFIVLNASQGLLMT
MFMTLTGEVLEKWKSAIMERFGLAEAHQDNGQTTTTHIRQTTGRTTEATAGGTGSAVEIPMTIMTDVEEDRARHRPRKPRQNKGRNA
LPSNKPSTAGGTEAKLDETDTAGNVQTLAGEVGTTASGTDVATEIIPMKTFVNAEGDEDENSAQDRDEDETSF
>Bbel_145670F

MGNPSTTGTSLALVWGLCCCLSGAFATIDFNVALGKTAFQTSTLNGMHGPGVASLAVDGITNIYMNYGTATCTHTQEETDPSWWVDL
GRSYMIDRVVIFNRMDCCSERLNPFNIHIGDSDQVSENPRCGGDHQILVTKPTVSIQCRWMAGRYVGFRLTGPSRILTLCEVQVITVGE
CSNGYWHLPH
TRRCYRAYDTESEYDQALATCREEGSTLAMPGDSATNDFLVALKNAADTTKVFYFGMTRSDDETWTYARGSQPMEWENWADGQPS
GGKERCATYLPGNSGDLSSNNRNKWNDVGCKIDAGFICEFEPCQVGDGASYRGTVSVTRTGKTCQRWDRLFPHVHSKLFEHPSSEL
EENYCRNPDGEPGVWCFTEDPSVRFELCEVPVCAPKKWREDLRCGRGYKTEDIEISECDPGSNFPCCSPDNWCGNTAAHCDCLAC
VDYRNTGICANGTIIDLTQVCDGRDDCGGNTDEQSCCERKGHRSCSNGRCLEWSIQNAKDPVCDGVRSCADGSDELSCPLSVCDNG
ALFHPFGRCDGRDDCGDNSDEKNCDCSYLHDKGASYRGRGNQQHFCQYWTSQYPHAHNHTPEAYPSAGLERKYCRNPDGKDRP
WCYTNNATIRWMYCDDVFACDELPTSCFFTNDKGRTYTGHINRAGDRLCQRWDSQSPHSHPHTPHAHPDAGLEENFCRNPDNKDR
PWCYTTDGTETWGYCDVTECPDPTSWDYVPRSCNGSRISCKGSCGKSYKQFYSCQCDQDCIFFGDCCSDIGEVCSLNTTAQKPSDR
DHEKWKCLSGYDKGRSYWLLTWSIGCPCKIVQQVSPIETYSVLFVTIHNSSNKTSHLQNCSRPILNFTSEEFEILPNGSVRLLSSNMTC
PAEQVAILNTEASVCGDCLLEYFSNDTLQTSDPVQHWLTLGLVIVSDVAVSGFVVHTYRSGQWKKIPEKLKVQMMTCMAVAVTQFVG
RVFLSPGPGCTAYAILLHYFILTAFTSMNVLAVDIFLTFREDSERAELYKYILYTWLVPVPIVLVTVIVEFGSSVRVGYGEQCWIGNPTASL
VAFGAPVLCALMVNLVFITLVLLDIRKSFQIASTALPRSEISKIWVYMRISFLSGFTWILGFIVPFVNVAVLDYIFIVLNASQGLLMTLFLTMT
GEALEKWKYAIMVRLGLIETDEDNGQDTTTSNRRTGRTEVTTAGGTGTAIEMTIMADVEENRARHRVGKSRQDKGRNATASNQPTTA
GETEADETGTAGNLQTLAGGVGATASGTDVATEIPMETFVDAEENQAEDRRSACAEDETSF
>Bbel_134080R
MATAGPYDDGTSDEWDSDAPDNPRDHEYGPVEDHWRSWGRKAQAAGVDPWTRNRGKVREVYARYRLEKAYAYLRHRKGELRGL
NRVRGLNRVIARLDAEINDFNVALRKTAFQTSTLNGTHGPGVASLAVDGITNTYMNYGIATCTLTQQEADPSWWVDLGRSYMIDRVVIF
NRMDCCSERLNPFNIHIGDSDQVSENPRCGGDHQILVTKPTVSIQCRWMAGRYVGVHLTGPSRILTLCEVQVITVGECSNGYWHLPH
TRRCYRAYDTESEYDQALATCREEGSTLAMPGDSATNDFLVALKNAADTTKVFYFGMTRSDDGTWTYARGSQPMEWENWADGQPS
GGKERCAVYFPDNSGDLSFNNRNKWNDVGCKIDAGFICEFEPCQVGDGASYRGTVSVTRTGKTCQRWDRLFPHVHSKLFEHPSSGL
EENYCRNPDGEPGVWCFTENPSVRFELCEVPACAPKKWREDLRCGRGYKAEDNTPAAECDPSSNFPCCSPDNWCGNTAAHCDCL
ACVDYRNTALSACDNGVLFHPFARCDGRDDCGDNSDEKNCDCYYLHDKGASYRGRGNQQHFCQYWTSQYPHTHNHTPEAYPSAG
LERNYCRNPDGKDRPWCYTNNATIRWMYCDDVFACDELPTSCFFTNDKGRTYTGHINRAGDRLCQRWDSQSPHTHPHTPQAHPDA
GLEENFCRNPDNKDRPWCYTRNGTETWGYCDVTECPDPTSSDYVPRKCNSSCKGECGQSYKQFYSCQCDQDCSFFSDCCSDIGV
RLLSSNLSCPAEQVAILNTEASVCGDCLLEYFSNDTLQTSDPVQHWLTLGLVIVSDFAVSGFVAHTYRSGQWKKLPEKLKVQMMTCM
AVAVTQFVGRVFLSPGPGCTVYAILLHYFILTAFTSMNVLAVDLFLTFREDSERAELYKYILYTWLVPVPIVLVTVIVEFGSSVRVGYGEQ
CWIGNPTASLVAFGVPVLCALMVNFVFTTLVLLAIRKSFQIASTALQRSEISKIWVYMRISFLAGFTWILGFIVPFVNVAVLDYIFIVLNASQ
GLLLTLLLTMTGKVLEEWKYSIMVRLGLIEANQDIGGTTTTSNRRTRRTEVTTAGGTGTAMEMTIMADVEENRARHRVGKPRQDKGRN
ATASNQPTTAGETEADETGTAGNLQTLAGGFGPTASGTDVATEIPMKTFVDAEENQAEDRRSACAEDDTSF

>Bbel_132690R
MHPDEPSDWCSNDSVPYNISQVSPQPSGDVVLLGVSDNLTCNATEYLIVENQAYVCKSCLRRYDSGTGIDAKDLYSQTLLTTTMLSLSI
ASGIGFIAHGVYYRKFSSTPDRLKLQLVVMLTLAEC
LFISRSFLADLERFCTTAAILLHYCLLATFLSMSSLAFDLFRKLCCAQFSSYGVPYRRYAVFSFGLPLVLVSVCAFLDFSPWTEAFRVGY
GGQRCWIDNDWASLLAFGVVVGLVVLSNAYFLVWIVVSLYRSGRETAAARTGAEIALMRVCFRITSLMGLTWALGLVAPFVDNAVLWL
VFTGMNSAQGLLIVIALTVKNCKACGSKSELSETGKGSLGSSEKGRLYHVTVRQEGCPNISNNITKL
>Bbel_069800F
MGTVRTTASLSATAIALMFLLAEVAAQNDGPRPGELGPPPIGPPPVPVATTPPPLLYVPTPPACHPNDTCSDRCGTWSPGTMCQCDP
RCRLFDDCCKDVDTFCGGSLTTDDLETASRPVVMADSCTWRCGPDVLSGAAETFCDCAESCVGNGTCCSDHCSAADGVPSGDPPR
AVPNDTAPYECLWNGGTDSYVWMVASCPPAWTDGVTREGCLRIDVDYTDVNDVIFYNPVSDVTSDVAYRNVYCAACNGAHNVTFW
TLGISGNLELVGSEEARLNATIAAIQSGQGRVYFRPLVKTRECVPRKTNYRGDQGGHCFAELCAGHAAYVSGDTGIYRNVYCAQCEG
VHPQELEVLSCGMTSCRSCGGASQLHLLSLSILFNFNPLNSHSASKCPPGSAYDLLSRQCREIMTSPTQAPPGEICVEGQTQVLSGGG
ISVVGISIDEQPCQEFSSSNSTVDNCTDCDKPTPETYPTTEAHHTEDNVSGILNIALTALSAVGGIVYLALCVVTREYHSIPGRLKMQLVIT
LTAAQSVFLGRVVAQVSTAFCYFVAVLGHYLFLAAFLTMSALAFDLHSTFSRGISEGRFNSYRLYILYTWLLPAVLVGVTAFVDFCPCIT
TVTVGYGGQACWIGEPYALLAVFGGPVALVLVTNAIFMVLTVVSLQKATKAVSKIRRDKSRSLLWVYVRITVLLGFTWGLGFAVSFVRS
LALDYIFTVLSATQAFLVALAFVLNRSMLRRCEAAFRRNTSSKKNTHATHLGDGSRLSRRESPWRVASGSANSGFTTSSTSDCQPHP
WPMSSDSDQTSFVGASEMAVFKSHEKNFM
>Bflo_gi_XP_002608881
MATTADNFSQSLDSVPDEPSIQDLEHSIEDALNVGHLDDEIVTAIEESFQDDYEKYNSTSGDLDTFVEDELHVPDCGTGFVPFDKEEWK
ILHPNGTLRLLLSGLVVDEEHYVRFGNFVFICNDFLKFSSNFTVSLSSALPEGYTPQEILTLALSSLSFVSMLVFIAAAVVKKMYRKIPDIL
KLNLTCSLLVAQTIFVFSDEIPLGKFCVFLAILTHYMFLVAFLTMNVLAFDLFRTLGRVSGAMVFRSVKGYMCYIWLAPCLIVTISTIVEFSL
ESKFLGYGTDVCWIKNPVALVVTFVAPIGLVLASNMYFFIHTVLNITVSSIRTARARHGATYSQLQAFLKISTLMGLGWIVGFIAAGTQQD
ALWYIFIILNSSQGFLISISFMTSPRDKAADNKRKGRASIVRGKNRLPSPGGMQQSGFQMCRANLSCSTSNVTNVSTASMASIESKDDS
KNTAIVTADVIAVVADD
>Tcas_Mthl16
MIKILLQIALLIPTCFSSNPCCRNNNAISERRCIDGSPLQGLSCPQKYLIEKDSDIEVDSDDHLNIASSRISPNKYCVSSKNDTVVYIVCFNE
ESTGNTIYIVHAVLELISVFFIVITIFVYLQLRDDLLDLQGKSILHSIAGLALSFLTLAVNQLLPGGLERPYCIVLAYIIYFSMLYSFFWLNVLSF
HIWRVIVKPRFLQILTPYWHYIYCGFGCGGPTFLTIILSIAQSMNESDSHPGFGETKCWFKSTKTQFLYFYTPMAVLLCLNLLYYGSTIAT
LWRSLKTVDEKKDKVLKYRLLLCVKLFFIMGISWIFELLSAAFQESNPLQVIWHVTDVLNTLQGILIFLILVVFRKRVVRGLADRSFCGIRL
PGRWRGAADDECGEIEEELNLSETHVQKN
>Bflo_XP_002610765.1

MYLVAVVLPLVLAFPAVKGPRYREEGGLFAKGIVDAQDRTIWIRQPTSEYTRLNDGEQRSCKTAKRSIPEVEDEISNLSYCLPHGTCEG
HCGEPDPSNLYYQSYFNCRCDADCELFQDCCPDYSTVCAYHHPEEGSVFCGEGFSYDFWNKMCVSNQNSSHVLQTIHITLTIQIQTY
SSLVNEELEHQVIGAVTGLFNISQAGLQSINVNIDSPWQQVNTSASSGERIPPDSPVQNGPVGESFFSMTFSLEIPITDNFMNGNYSYFL
DNLYANLEDFDFYIAGVPVNAIDSREDVDVDSFQCRNGTIARVYDFMDFVFMTDQENNTILHVNSTCKSFSEDEFTVYSVSMHASKFG
ELTTQIAQVLVCVDETSCPIV
PLNQSEYQLLPNNSLLYISCAYGDHLVPPSDYCIINGQPHVVFCENSTADSGVDYQPVEAPTAITADRVVTFVAMVVSLTTLAATIVTYLL
FSSLRTLPGLTILNLSTSLFLSQVVFLSSVNRENISSDLLCKVRSDHMRKALRYMVYAWGCPLIIVSICAVLEFVDVGPLKDVGFGYGGT
VTCWISQLYPLIFAFFVPLAIIIVANAILFIITVISINRTRSVQGKNARQNVKVYVRLSTVMGFTWIFGFLAVVVNARIITEILWFLFIIFNCLQG
VFLFIAFVCNTRVLNLYKKRLGLKRKGGQLVRGMSTPPCSPIKKSPTETTLVPSTPPVSINTISEKCMKKSPSQTFLIPPCSFEFEPTPLY
PPPQENEEDIVTRL

Text file S2: Multiple sequence alignment of 7TM domain used in gene tree reconstruction.

```
 278 365
Dmel_methuselah                               CD-GMFYLDKTLFE----N-GTFFRH-F----DTLRKREY--CLDGIA-PHNCLI-----
Dmel_Mthl2                                    CD-DMFYLDETLFE----N-GRLLRH-Y----DYLDKSEY--CLEGII-PHNCLI-----
Dmel_Mthl3                                    CP-RMYFLNETLFE----N-GSLLRH-W----DELSKREY--CVD-IA-PHFCPL-----
Dmel_Mthl4                                    CP-RMYFLNETLFE----N-GSLLRH-W----DELSKREY--CVD-IA-PHFCPL-----
Dvir_XP_002046457.1                           CS-EHFHLHGTLME----D-GRLLRH-F----DSLSKQEY--CLAGLV-PHNC-------
Dvir_Mthl11_XP_002053031.1                    CN-DHLMLHGTLFE----N-GTLVRH------DHLSKRDY--CL--LQ-PHHC-------
Dmel_Mthl11                                   CE-RHYSLDMSLYE----N-GSLFRH-F----DYLSKQEF--CLTGIV-AFNCIQ-----
Dvir_Mthl11_XP_002060419.1                    C--DFIGLHQTLFE----N-GILFKH-T----NNKNKGRY--CLD-LG-AHYLAE-----
Dmel_Mthl6                                    CE-KVVSVDQKLYE----N-GSFMIKPD----VTLSKQWY--CL--IL-EHVYIP-----
Dmel_Mthl7                                    CD-EMIYIFNFLEE----D-GKFWV--T----VFMEKQDY--CLSDII-RHRCTS-----
Dmel_Mthl12                                   CD-EIVSLDETILK----D-GSILLH-T----SILSNDQY--CLSDII-NRRCYR-----
Dmel_Mthl10                                   CP-KMFSLDDLLFE----N-GSMLRV-D----DLIRKNEF--CME-IH-PANCDM-----
Dvir_Mthl10_XP_002046455.1                    CT-EMFSLDTQLYE----N-GTLLRE-D----DYIYKNEF--CLD-IN-PANCDM-----
Bactrocera_dorsalis_|XP_011201382.1_Mth2like  CT-DGYMLDQELRE----D-GTLYRL-A----DVLSRREY--CLG-LN-PMNCPM-----
Ceratitis                                     CA-DVYPLDKELFE----N-GTLLR-------DYLSRRDY--CFS-LN-PMNCPS-----
Mdom_XP_005175058.1_Mth2like                  CA-GAYALDSVIFE----N-GTLLRH-Y----DYLSKRDY--CLT-LN-PMNCPI-----
Dmel_Mthl13                                   ID-EMMGLDETLYK----N-GTIYIH-S----DIQTKEEY--CFSDII-SHKFSS-----
Methuselah_Dpul_EFX89685                      CQ--MFPLDEYVLP----D-GQIYLPYY----PDRYTRDY--CIEGRQ-ALLC---FPP-
Dpul_EFX89686                                 CQ--AYSLDEYILP----N-GRIHTPAL----G--YHDDY--CIDGLQ-ALMC---FPP-
Tcas_Mthlsc_XP_008190998                      CP--RILLQRYVQV----N-GSLFLPGF----KMRNPENY--CLKM---ALVCLT-----
Agam_XP_316287.2                              -P--VYELDESIAR----N-GSLIIADE----LQR--NQY--CLGF---GLVCFT-----
Mrot_XP_003703117                             CP-GKFLLDAVLLA----N-GTLQMTTN----K--AAWTY--CFEK---AIVCLSNFPP-
Esup_GAVW01003911                             -P-GRYRLDEVLSS----D-GLLIQTME----NDRGPKDF--CIET---PLAC---FPP-
Cbol_GAYO01008982                             -S-GRYLLDRYIKS----D-GFLVQPYE----NEQGPKGF--CIDA---PLVC---FPP-
Cbol_GAYO01127990                             ------------------------------------------------------------
Cbol_GAYO01128358                             ------------------------------------------------------------
Asia_JW948170_JW948387                        CA-PPLSFDKYVLP----N-GNIYSLDQ----GTLAPPNY--CISIFS-GSFC-------
Asia_JW969407                                 ------------------------------------------------------------
Asia_JW965784                                 CR-PPIPFEEFVLP----D-GRIYSVEQ----GF-SSPEY--CITHLF-GLFCAT-----
Dpul_EFX89687                                 C----YDETEHILS----S-GQMYAPDY----PERATYQY--CVQKMK-GLRC---FPQ-
Pxyl_XP_011552150                             CMSDMIRLENHIRK----D-GKLHIESP----PLRTPDKY--CVDGLD-ALVC---FST-
Dpul_EFX74305                                 CK------HDHLRS----N-GLLFVQSQ----TDLITDEY--CVDGQR-AMVC---VAA-
Dmel_Mthl1                                    C--GGYSI--KLDR----NTAMLQLPHK------LSAGQY--CLEG-K-IIACQHLFSSA
Dvir_Mthl1_XP_002059559.1                     CL-SGYAI--TLNR----STGDLRLTHT------LSAVQY--CLAG-K-IIACTQHFA--
Tcas_Mthl1                                    C--QEVVI--EMHK----N-GSLWLPKM-----LLPAANY--CLAGPK-IMICQEHVT--
Smar_012256                                   CYEDFVNCYEHLHS----NSRQLYN-------HFYPVGKF--YL--ME-AAVCYP-----
Dpul_EFX83097                                 CG------LNSMRP----D-GQIVLHLE----NDEKTDQY--CIGNVM-AIVCFP-----
```

```
Dpul_EFX83094                   CG------FDSIRH----D-GQIVLQLE----DDEKTDQY--CI----------------
Dpul_EFX83013                   CG------FDSIRP----D-GQILMQLD----NDEKTDQY--CIGNLV-AMVCFP-----
Ctel_216805                     CP--MILL-EAIIR----G-SDLYVNSS----G-HAFGTY--LL---S-AYICTNFSK--
Ctel_208644                     CP--MIRL-ETILH----G-SDLYVNDT----G-HAPGTY--QL---S-AYICTKVLT--
Ctel_63867                      ------------------------------------------------------------
Gdib_GASB01015634               CP----KLHQDLLP----N-GSLNV----LVWNIIDNSEY--LMVSFQ-ASVCLPVMP--
Dpul_EFX73784.1                 ----------------------------------------------QN-VSICSIQPD--
Dpul_EFX73786.1                 ------------------------------------------------------------
Skow_XP_006818657               CT--LVNL-----E----N-HSMYYSPT----EIFGPGEF--QI---T-LYVCNTFNQNG
Skow_XP_006821924.1             CP--RVRL-QEIFP----N-RSIKYN------D-YDTDEY--SI---I-IEICTPGYYDI
Spur_XP_011662057               CK-GYLRAFETHDF----E-DAIRVGTG----N-IGQGKF--QR---S-VQYCLEENS--
Lgig_75877                      ------------------------------------------------------------
Spur_XP_011660493               CA--LVSQ-LVDLG----N-GSYLYSPL----N-LSPNDY--LD---S-IFVCSYVLVNE
Spur_gi_XP_011675990            CA--LITL-DVIWS----N-GSVLYEPY----N-FDTDDY--IL---S-IQVCSFLDGSG
Spur_gi_XP_798567               CA--LITL-DVIWS----N-GSVLYEPY----N-FDTDDY--VL---S-IQVCSFLDGNG
Spur_XP_003723996               CA--VVLP-KSLKD----V-GNSTVVVY----NEYEDELF--EY---S-IRICVTHNNFS
Nvec_XP_001640473                   CS--GVFFKHVIFQ----N-RSIFVNKS----G-YNASEY--FV---T-THVCTEVRP--
Nvec_XP_001636770                   CS--GVFLEHIIFP----N-RSIYVNVS----G-LNQSEY--FV---T-TYVCTDLPP--
Nvec_XP_001628572                    CT--GTYVNETMIS----N-RSVYVNRT----K-FSHMEY--VT---S-LWICRNYSNAY
Dpul_EFX67288                   ------------------------------------------------------------
Dpul_EFX79407.1                 CN--VIHL-EVFFD----N-NTVLYNGS----D-LQPGEY--ES---G-LAVCLYISQKW
Dmel_Mthl15                     CP--KFRLDSTINP----N-GSLIIH------TTYTNTQY--CVQ-LY-TFLCFD-----
Dvir_XP_002060099.1             ------------------------------------------------------------
Cgig_10021040                   --------KETLAN----DIRSLPF-------D--------------I-YRICDILPLF-
Cgig_10015721                   CT--QIELDEEF-S----DMTRVY-IPS----VYFNINEF--HING----RICSESYKRI
Cgig_10001367                   ------------------------------------------------------------
Cgig_10006654                   CK--HIQFEEIMDS----GIATVL-SPA----VL--PGEY--VTSN----SFCIDVYLAS
Cgig_10025895                   ------------------------------------------------------------
Spur_XP_011679633.1             CS--FFTVIEDYQG----N-QSVIL-------QMYDTEDI--LI---S-ISICYSDQVTP
Bbel_293770F                    -------FDEQILP----N-GSVRL--S----SSCPAEQV--VI--TS-ASICGDC----
Bbel_192410R                    CSKPILNFEEQILP----N-GSVHL--S----STCPAEQV--AI--TS-ASICGDC----
Bbel_145670F                    CSRPILNFEEEILP----N-GSVRL--S----STCPAEQV--AI--TE-ASVCGDC----
Bbel_134080R                    ----------------------VRL--S----SSCPAEQV--AI--TE-ASVCGDC----
Bbel_132690R                    CSNDSVPYSQSPQP----S-GDVVLGVS----DTCNATEY--LI--NQ-AYVCKSC----
Bbel_069800F                    CVE-----GQQVLS----G-GGISVGIS----IEQPCQEF--SS--ST-VDNCTDCDKP-
Bflo_gi_XP_002608881            CGFVPF--EEILH-----N-GTLRL--------VVDEEHY---VGNF--VFICNDFLKFS
Tcas_Mthl16                     CP-QKYLISDEVDS----D-DHLNI-------ARISPNKY--CV--VV-YIVCFN-----
Bflo_XP_002610765.1             CP--IVPLSEQLLP----N-NSLLYSCA----GLVPPSDY--CI--PH-VVFCENSTADS
Mvib_comp11351_c1_seq1_fr6      CYMTLIVSQDLVDSVFGLDSASISFNTSCTFWDGWWSTVG--C-TNHT-TCKCSHATNFA
Mvib_comp15999_c0_seq1_fr4      CQFSTFVPTQTIRSVVVLESEAFVFSTGCVWWDTLWTTSG--CVSTST-TCRCNHLTSFA
Mvib_comp15986_c0_seq2_fr4      CQFSTFVPTQTIKSVVVLD-AAVVISYTCVWWDSLWKTWG--CVSTAT-TCRCNHLTSFA
```

Mvib_comp15877_c0_seq1_fr6	CYLTVF--ASLIDSVVSVDRSRLRFSGSCVFWDSLWSKEG--CLTDYT-ECQCNHLTNFA
Mvib_comp15861_c0_seq1_fr4	CHLVEYFVAQTADSVVSLDDASVVFNASCAFWNSMWSTDG--CVRNST-TCKCNHLTNFA
Tcas_Mthl15	CHLSAF--MDNVNSIVHL--YPL-----MEFYDYKVDENG-YCM--HA-----GSRNNYF
Cowc_CAOG_00386	CKFIVY--NA---SIVSLD--PVEFNSDCAYWNNLWETNG--CVVVNV-TCACTHLTNFA
Cowc_CAOG_00812	--FVVY--DS---SVFSLS--PIRFSSGCAFWNNNWDTNG--CAATSI-DCACTHATNFA
Cowc_CAOG_03584	--FALYFPNSAVS-VISLE--PVTFPLPCTFYNQQWDAEG--CSLSST-QCQCYHLTNFA
Cowc_CAOG_03451	--FVLYFPDSQVF-VISLE--PVTFAMPCSYFDQQWASDG--CTLTSA-KCECYHLTNFA
Cowc_CAOG_03413	--ASIMF-GEFIGSILSLE--PLRFAATCVWYDSSWSTTG--CRIDTT-ECSCNHATDFS
Cmil_SINCAMP00000022659	--FSFYF-ETILNSIIALN--SAKLKFSCVFWDGGWSADG--CAKTQT-TCECHHLTNFA
Hsap_ENSP00000369198	--FNFFF-ENSLISVISLR--NVTVNPSCVFWDGGWSDNG--CKRRET-ICTCSHLTSFG
Hsap_ENSP00000359686	--FNFFF-VTALTTVVSLD--PVVIGGNCAFWDGGWNSSG--C-TNYT-ICQCDHLTHFG
Cmil_SINCAMP00000023038	--------NNRLNSVVALD--PVKIHPICVFWDGAWNSTG--CITDYT-ICGCNHLTHFG
Drer_ENSDARG00000054137	--FMFFF-NGTLNSVVALD--PVRIEYQCVFWDGGWNSDG--CGSNRT-VCLCNHLTHFG
Hsap_ENSP00000356581	--FTFFF-QRTLVSVMALD--PVQIRTQCAFWDGGWNTSG--CHSDET-VCLCNHFTHFG
Drer_ENSDARG00000088137	--FTFYF-NQKLNSILGLE--NIEFQPVCVFWDGGWNRDG--CMSTET-ICSCSHLTSFA
Drer_ENSDARG00000087813	--FHFYF-SNTLNSVVSLI--PVMVQNKCVYWDGGWNDRG--CVSNQT-SCSCDHLTHFG
Drer_ENSDARG00000088937	--FNFFF-GDKLNSILGLN--EVLIGPICVFWDGGWNPSG--CVVSET-KCACNHLTSFG
Cmil_SINCAMP00000004297	----------ILNRVVGIE--PVTI---CVFWDGHWDPAG--C-TRQT-ICYCHHLSFFA
Smar_003150	--FISFFRND---QVIILR--PVTYSPNCAFWDKKWSQDG--I-QSFT-WCYSSHLTAFS
Cgig_10006507	--IITYFIEEVLKQVLSLN--PIHYESACVYWENRWSTEG--VVTGET-HCYTTHLTSFA
Lgig_74363	-----------------------------------WSSEG--MVISVT-ECQSDHLTSFA
Dpul_EFX80180	--FVSYF-GQ---AVIALE--PVTYNSSCVYWDKEWSTNG--VTTEMI-ECLSDHLTAFS
Skow_XP_002733572	--FISYFKNDVLNSVIGLE--PIKIRGNCVYWDGGWSTDG--CSSSVT-TCECDHLTNFA
Skow_XP_002735776	--FISYFKNDVLNSVIGLE--SVKIRANCVYWNGGWSRDG--CLSSFI-TCECDHLTNFA
Skow_XP_002734798	--FISYFKNDVLNGVIGLE--PIKIRGNCVYWNGGWSTDG--CSSSFT-TCECDHLTNFA
Skow_XP_002733569	--FISYFKNNILNSVIGLE--PVKVSENCVYWNGGWSTDG--CSSSVT-TCECDHLTNFA
Skow_XP_002733570	--FISYFTNDMLNSVIGLE--PVQINEV----NGGWSGDG--CLISLI-TCECDHLTNFA
Cint_ENSCINP00000004627	-------------S---LE--PVKISSLCVFW-GYWSSNG--CNTSQT-VCHCSHLTNFA
Bbel_144770R	--FAVYFKIDVLRGVISLD--PAKIEDSCVYWDGGWSSEG--CYAAHT-VCHCNHLTNFA
Adig_17884v114019	--FVIYFQESSFMSIVSLK--PVELQELCVFWNGNWSDRG--CIFERA-KCHCDHMTNFA
Bbel_063250R	--YIIYFQTKRINSVIALN--PVVTNSTCVFWDGAWSTEG--CVIDRV-VCECNHLTNFA
Bbel_062450F	--YIIYFQTKRINSVIALN--PVVTNSTCVFWDGAWSTEG--CVIDRV-VCECNHLTNFA
Bflo_gi_XP_002598501	--FIIYFQKRSINSVIALN--PVVTNSTCVFWDGAWSTEG--CVIDRV-VCECNHLTNFA
Bbel_230420R	--FIIYFQTKRVNSVIALN--PVVTNSTCVFWDGAWSTEG--CAIDRV-VCECNHLTNFA
Bbel_130640F	--FIIYFQTKRVNSVIALN--PVVTNITCVFWDGAWSTEG--CAIDRV-VCECNHLTNFA
Bbel_156760R	--FTIYFQTNRVNSVIALN--SVVTNSTCVFWDGAWSTEG--CAIDRV-VCECNHLTNFA
Bbel_238310R	--FIVYFQTTRVNSVIALS--PVVTNGTCVYWDGAWSAEG--CAIDRA-VCHCNHLTNFA
Bbel_204650R	--YILYFI--AVGSVIGLK--PVVIQDFCVFWDGAWSSEG--CKEELY-TCEVYHLTNFA
Bbel_060890R	--YTLYFV--AVGSIIGLQ--PVTIEDFCVFWDGAWSSDG--CQEETY-TCTFNHLTNFA
Bbel_079450F	--YTLYFVYQAMGTIIGLE--PVIIQSFCVFWDGAWSTEG--CMQTRY-TCACNHLTNFA
Bbel_121130R	-----YAVSQAVGTIIGLE--PVITGSFCVFWDDAWSTEG--CKKKRY-TCACNHLTNFA

```
Bbel_281940F                             --YTLYFVSQSVGTIIGLE--PVIIQSFCVFWDGAWSTEG--CMETRY-TCACNHLTNFA
Bbel_299040F                             --YTLYFVSQAVGTIIGLE--PVIIQSFCVFWDGAWSTEG--CMETRH-TCACNHLTNFA
Hmag_XP_002158889                        ---------------------------------GSWLRTG--CYNTYF-TCACDHLTNFA
Skow_XP_002740291                        --FIIFFQNDMVNSVISLD--PVKISEDCVYWDGDWSSEG--CDVKRV-VCHCYHLTNFA
Adig_16756v113082                        --VVYYFASRTTSSVLSLK--PVIIADQCVWWDRGWSTDG--CRIVTI-ICECNHMTNFA
Nvec_NVE6184                             --FVYYFKRDTESSVIALE--PVTIPDMCRWWDGDWSAEG--CEIYTV-VCQCDHMTNFA
Spur_WHL22.383522                        --FNVFFIFNSANSVISLE--YINFMSGCSFWDEDWSQDG--CVSSSV-RCACDHLTNFA
Spur_WHL22.147647                        --FNVFFIFNSANSVISLE--NINFMSGCSFWDEDWSQDG--CVSSRV-RCACDHLTNFA
Spur_WHL22.754506                        --FNVFFIFNSANSVISLE--SINFESGCSFWDADWSRDG--CVSSGV-RCACDHLTNFA
Spur_WHL22.184504                        --FNVFFIFNSANSVISLE--FINFKSGCSFWDENWSQDG--CVSSGV-RCACDHLTNFA
Spur_WHL22.230519                        --FSVFFITNSAKTVISLE--PINFETDCSYWDSDWSQEG--CLSSEI-VCGCNHLTNFA
Spur_WHL22.226322                        ------------------------------------------------------------
Spur_WHL22.138572                        --FSVFFITNSANTVISLE--PINFESGCSYWDSDWSQEG--CLSSKI-VCGCNHLTNFA
Spur_WHL22.264380                        --FTVFFITDSANTVISLE--PINFKTNCSYWDGEWSQEG--CISSEI-VCGCNHLTNFA
Spur_WHL22.73425                         --FVVFFPHRKVLGILSLD--PVVIEGLCVFWDGDWSNEG--CARTGI-ICFCNHLTNFA
Spur_WHL22.3020                          --FFVFFQRGRVGSIISLQ--EIETNVTCVFWNGEWSTRG--CELPRI-RCLCNHLTSFA
Spur_WHL22.3487                          --FFIFFLGGTVGTVISLQGGEVETNVTCVFWDGRWSSKG--CENPRI-RCLCDHLTSFA
Ocar_g3634_t1                            --FLVYFLTSKVNSIVSLD--PVKIEKLCVFWDGEWSTDG--CVSQYS-VCKCTHLTSFA
Nvec_NVE23935                            --FIYYFQAELVTSILSLE--PVALSGVCVYWDSIWSNEG--CTNG---GCACNHLTSFA
Bbel_163260R                             --ATLYYLSDIVNSVISIEGETITIKSQCNYWNSDWVNDT--CSSNHT-TCVCNHLTNFA
Hsap_ENSP00000420931                     --FVLYYLHSIVNSVITLD--PVVFQSECSFWSGYWSTQG--CLTNHT-TCSCNHLTNFA
Cmil_SINCAMP00000005886                  --FVLYYLHTIVNSVVSLD--PVVFDLKCSFWSGYWSTQG--CLSNHT-TCSCNHLTNFA
Drer_ENSDARG00000061121                  --LVLYYLYSIVNSVITLE--PVVFQSECSFWSGFWSTQD--CLTNHT-SCSCTHLTNFA
Hsap_ENSP00000340688                     --FILYFLASVVNSVIALD--PVIFEDKCSFWNGYWSTQG--CVSNHT-TCACSHLTNFA
Drer_ENSDARG00000089292                  --FVLYFLRGAVNSVIALE--PVVFQLECSFWNGQWSSQG--CITNHT-TCSCSHLTNFA
Cmil_SINCAMP00000012795                  --FILYFLYSAVNSIIALD--PVVFDPDCSFWNGYWSTKG--CVTNHT-TCECHHLTNFA
Hsap_ENSP00000359752                     --FIIYFLSTAVNSVISLD--PVLFDPDCSFWNGYWSTQG--CVTNRT-TCACSHLTNFA
Drer_ENSDARG00000069356                  --FALYFLLSAVNSVIAIE--PVIFDMQCSFWNGYWSTQG--CIANHT-TCSCSHLTNFA
Cint_ENSCINP00000004251                  --FALYYMQDNVDSVVSLK--PVKI---CVYWKGVWSSKG--CVSNHT-TCSCNHLTSFA
Smar_011200                              --FFTFILVSVLNSIISLQ--PVTIRDDCVFWDHTWSDDG--CKTNHT-VCSCTHLTNFA
Dpul_EFX79819                            ----------------------------CVFWDQEWSSDG--CVTNST-RCRCNHLTNFA
Ctel_226868                              --FLSYWLRSEVISVISLE--PITYSHNCAFWEGKWSTQD--CVTNHT-VCQCTHLTNFA
Hrob_P80944                              --MIVF---------------------------SEWSNEG--CMHNHV-TCSCEHLTSFA
Spur_WHL22.6202                          --LLAYFLLGAVNSVLSLK--PVNLTGDCSFWKGEWSDRG--CVNNHT-ICSCDHLTNFA
Tcas_latrophilin_Cirl_XP_008200380       --FVAFILVTMLNSVISLE--PVRLQTECVFWDSAWLEEG--CETNHT-VCLCDHLTNFA
Dpul_EFX83073                            --LFAYILATIVNSVISLQ--PVTIRQDCVFWDSGWSDEG--CLTNHT-QCRCDHLTNFA
Skow_gi_XP_006820674                     --FLAYLLSDIANSVIAMS--NVIFKTDCAFWNGAWSDYG--CVSNTT-VCACNHLTNFA
Bbel_191840R                             --------------VIALE--PVVFTQECSFWKGYWSGEG--CMTNHT-SCSCNHLTNFA
Cmil_SINCAMP00000011725                  --LISYIIDPDVVSIVTLE--PVTLQQNCVSWDGIWSTNG--CLANHI-ECSSRRVASFA
gi_XP_009668681                          -----------------L----------CSAGD-----------SG---DIKLQHLSSFA
Skow_XP_002733056                        --FFMYILADIVNSIISLE--PAVLQTYCSYWNGIWSDYG--CVNNHT-VCKCSHLTNFA
```

```
Adig_11188v107943              --FVSYLM--EKNS-MSNE--E------CVFWNGFWSGEG--CTRNHT-TCQCFHLTSFA
Nvec_NVE16100                  --FVSFLL--HVDSVVSFE--PITIEKPCVFLNLSWSGDG--CGTNHV-QCHCRHLTSFA
Nvec_NVE16098                  --YVSYLLRNTINSVLSFE--PVTIKANCVFWNGFWSNKG--CNTNHT-ICKCYHLTGFA
Bbel_166310F                   --ALLYYLANTVNSIISLQ--KVTIDAECSFWNGFWSSKG--CKSNHT-ICECDHLTNFA
Adig_11540v108276              --YAVYFPDDFVNSILGFK--HIQLEVECSFVDGTWLERG--CVDESV-TCACNHLTNFA
Nvec_NVE9578                   --MSTY---HVIRTVLSLK--PAEVKGKCSYWDGRWESDG--VNSSHV-ECLTHHLTSFA
Skow_XP_002735837              --LLVFFT--RPSSVLSML--PVNIQNHCVFWNGYWSEYG--CTTLYT-WCACNHTTSFA
Adig_19406v115301              --SVIYVIVTLPNTVVSFK--PVKIRVSCVFWRSLWETRG--CVSDKT-TCKCDHLTIFA
Bbel_260260F                   --AVLYRYSESLNSVVSLD--NVTVQEACVHLDGVWSDAG--CHSNHT-VCSCNHMTNYA
Nvec_NVE10940                  --SLNYLK-----GVVRVG--KVDIASACVFWDGDWSSGG--CVATDV-TCSCNHLTHFA
Adig_2760v120555               ------------------------------------------------------------
Nvec_NVE16489                  --IVVLLYDEAVRSVLSI--HHVTFDNICVYWDGAWSSEG--LINKIT-ECWTSHLTNFA
Nvec_NVE13836                  --SALYILAKFLNSIITMG--GVNIQFKCAFWNGSWSSHG--CDSDKT-ACLCDHLTHFA
Bbel_161400F                   --SAIYYFVNTVNSIISIG--NVTIEQTCVFWDGNWSGEG--CATNHT-ICECNHLTSFA
Scil_13054                     --ASSFLLSNRLLTVVSLD--PITFNSTCAFYNGGWDNDG--LNEGET-LCSTVHLTSFS
Nvec_NVE18487                  --SIIFFLDGALRSIISFD--PVKMQNYCVFWDGAWSRDG--CSRGDV-ICACDHLTNFA
Smar_006024                    --FTKFLLDQTLNSVIGFD-NFVIINETCTFWNKTWDTEG--CHSNHT-ICYCNHLTNFA
Cint_gi_XP_002121696           ------------------------------------------------------------
Tadh_XP_002113325              --VIIYFSSSDVNTIVSKQ--PTEFNYKCSYWNSNWSTSG--CVQYRT-HCRCNHLTHFA
Skow_XP_002734724              --VIVYIIIESVVSVVSEN--PMYLQEPCVYWEGIWSTDG--CYSTDT-TCRCSHLTNFA
Scil_9494                      --FAIYFMLSSVGSIVSLDGDPARFNTTCSFWNRIWSSEG--CVDSHI-VCSCNHLTNFA
Scil_49553                     --YILYFKANSLVSIISLD--PALMESLCVFWDGGWSNQG--CVNGRT-VCSCDHLTNFA
Scil_68080                     ----------------------------CVFWDGGWSNQG--CVNGRT-VCSCDHLTNFA
Scil_63570                     --YILYFQVNSVGSIISLE--PAFMPGTCAFWDGGWSSLD--CTNSRT-VCQCSHLTNFA
Scil_41456                     --YILYFQGNSVGSIISLT--PATMDGTCVFWDGGWSSEN--CTSDRT-VCQCSHLTNFA
Scil_70266                     --------------ILS--------KSACVFWDGGWSDQD--CSSGRI-VCRCNHLTNFA
Scil_27094                     --FILYFQARTVASIVSLS--AMSFDISCVFWDGGWSREG--CINGRA-ACECNHLTNFA
Scil_47986                     --FVVYFNNDRLATVLTLQ--PVRIKGYCVFWDDAWSEDG--CISSAV-TCLCSHLTNFA
Cint_gi_XP_004225576           --AITYVENDKVNTILSLN--KVTVNINCVTWDWVWSKPG--C----VPTCYCHRFADFG
Cint_gi_XP_009858460           --IVHHFP--NFNSITALD--PVKIHSKCRYWDTNWLPDG--C-HTPP-LCQCNHLTNFA
Cint_gi_XP_009860041           --LAMYFP-----SVISLE--PVEVHGECKYWDSKWADDG--C-ETPT-QCLCNHLTNFA
Cint_gi_XP_009858263           ------------------------------------------------------------
Cint_gi_XP_002124051           --FIVH----NTSGIVTLR--PVTYNSTCVYLDGDWSDVG-LC-GT-T-TCQSNHLTSFS
Scil_289                       --FIVFFTREIVSSVISLE--PIQISGDCAFWDGGWSQEG--CMSTHV-TCQCTHLTNFA
Scil_227                       --FIVFFTREIVSSVISLE--PIQISGDCAFWDGGWSQEG--CMSTHV-TCQCTHLTNFA
Cint_gi_XP_002129016           --FTGYFRKSVINSVLGLN--PVEVQTNCVFFDGGWSQEG--CSSSAV-TCQCNHLTSFA
Smar_001588                    --YTIYLLTGAVATIVSLK--SIRLNKHCVYWKGRWAIEG--CLLNAV-NCTCNHLSSFA
Drer_ENSDARG00000069185        --VIIYFLNRVINTIVSLR--PITLETECVFWNGAWSSKG--CIRNHI-SCQCNHMTSFA
Drer_ENSDARG00000058259        --VMIYLLNRVINTIVSLR--PILLETQCVYWNGAWSSKG--CVRNHI-SCQCSHMSSFA
Cmil_SINCAMP00000020331        --VIVYLLNRVINTIISLK--PITLVTECVFWNGGWSSRG--CVRNHI-SCQCNHMSSFA
Cmil_SINCAMP00000017405        --IIIYLLKRVINSIVSLN--PITVETECVFWNGGWSSKG--CLRNHI-SCQCNHMTSFA
```

Hsap_ENSP00000271332	--VIIYLLKRIINTVVSLK--PVTVETECVFWNGGWSARG--CVRNHV-SCQCNHMTSFA
Hsap_ENSP00000262738	--VIIYLLHRIINTMVSLR--PVLVEVECVFWNGGWSARG--CLRNHV-ACQCSHTASFA
Drer_ENSDARG00000019726	--IIIFLLKRVINTVVSLK--PIIVTTECVFWNGGWSSKG--CVRNHI-SCQCYHMTSFA
Bbel_010420R	--TLLFVMDKVINSVLTLD--PVYLQGECVYWDGAWTTRG--CTRNHI-KCGCDHLSSFA
Skow_XP_002741140	-----Y--------IME---------------NGGWSTKG--CQKSRV-ECACTHMTQFA
Cgig_10006474	--YIMFLLGRAVNAVFTFE--PLKFVKDCVYWKGQWSSEG--CDKKYV-VCSCSHMTSFS
Nvec_NVE25349	--YMIILLYNDVNSVVSLE--PIHIQVNCVFWNGGWSTNG--CIINHT-TCACNHMTSFA
Scil_7737	--VATFFSASPVRQVAALS--HVQLEAKCSFWDGGWSRQG--IVNTVH-QCSTNHLTHFA
Scil_14656	--VATFFSASPVRQVAALS--HVQLEAKCSFWDGGWSRQG--IVNTVH-QCSTNHLTHFA
Lgig_154761	--NVLYLFSEELGEILGLS--NIQYEINCVYLDPYWTDTG--CLVASV-TCSCSHLTNFA
Cint_gi_XP_009862007	--
Sros_PTSG_09542	--VTFFFANRFVNSVLSFG--EAVFGNRCSFW-GRWSNMG--CVDVYV-TCSCNHLTNFA
Sros_PTSG_09543	--VTLFFANQFVDSVISFG--EVEFDNNCSFWRAGWSDVG--CVIKFL-TCRCNHLTNFA
Sros_PTSG_06041	--FAVYF-NQFISTVVLFG--TIRYTTGCVFWQGQWDTFG--CTNTVV-ECECNHLTNFA
Aque_2_1_37513_001	--NSFIFL--EAGSVVT-G-IILNF---CVFWNGNWSSEG--IVITNV-VCSSSHTTPFV
Aque_2_1_39648_001	--NILFFLSDETGTILSID-PPVQLTPNCVFWDGSFSPAG--IVSSNV-TCLSSHLTSFA
Aque_2_1_39650_001	--SIQFFLNDKISSVWSFE--PILIKIDCSYWNGQWMTDG--VVDNYV-VCSSYHLTAFA
Hsap_ENSP00000273352	--FVVYF---DFSQIISQA--SVDMKYNCVYWNKDWDTYG--CDGTFL-RCRCNHTTNFA
Cowc_CAOG_05333	--GVGFFPADGLPTVVSAH--PIEVSTSCAFYNNQWQESG--LVSTTT-TCQTIHLSSFS
Bbel_292050F	--------RNTVGSVISFE--AIKIEIPCVFVNNQWSAEG--CESTCT-ICSCNHLTNFA
Tadh_XP_002117818	--AGIYFLDQTLSSIIS-D-NAIHVSKNCMYWDRSWNNTG--C-LNKV-VCVCNHLTHFA
Tadh_XP_002111670	--SFMYLLRNIPQTVISLE--PLEYSNECSFWNGAWSNQG--C-FNHI-VCQCDHLTNFA
Tadh_XP_002118234	--SLAYLFFETPNTIISLE--SVVYSINCVFWNGAWSDEG--CVLNHL-SCQCDHLTNFA
Skow_XP_002740941	--KTLYFVSEKFNSMYSFE--DV-----ITFYN----------V---I------------
Tadh_XP_002113682	--KVLYTEADSVNTILSL---SIQFRSTCKQCNKRWLSNG--CISRYT-SCRCNHLTHFA
Adig_8886v105801	--SLSL--ANVLKSIISLE--PLVIKKTCVFLDLSWSTSG--C-KEHT-VCHCYHMTSYA
Nvec_NVE22429	--VVVFVENKTLRSIINLE--PIQLSMPCGFIDDLWSVAG--CATSHT-VCECSHLTSYG
Scil_14083	CRFIVYFNPNTISSVVSFN-DKAVYSSNCVHWDLSWSSSG--CLTTSV-TCQCNHLTNFA
Mvib_comp15494_c4_seq1_fr4	--VAVTFPSEFVPSVISL---PCPFRADCNFYSKEWVSDG--CSRGWL-ECCCTHFTSFA
Mvib_comp15911_c1_seq2_fr4	--VNVFFHAQTIVSVVQLNNATVRLASTCAAWNLKWERDG--CVSSVI-RCECSHMTNFA
Mvib_comp12816_c1_seq1_fr6	CTRVVMFQNDALSSVVTLDNATVRLNASCVAWNRAWQSDG--CVRSNV-ECECTHLTNFA
Scil_24201	--LVLYFVDSSIVSVVALE--DVVFNVTCVHWNRMWSSEG--CISNST-KCSCEHLTNFA
Aque_2_1_20823_001	--FVAYFPRDAIGSVIGLE--PVNINPKCVFWDGNWSTSG--CISSTV-RCECNHLTNFA
Spur_WHL22.71613	--VTTYIYDDVVVSVLSV---PFRFKADCKFWDKRWDSYG--C-STAS-VCVCNHTTSFS
Aque_2_1_37130_001	--PLVH--EVMVNSVITFE--PVRINTTCSFLDDGWIDDG--CTSGFT-VCNCYHLTTFA
Spur_WHL22.392553	--RAVYILNRKIGSLLSFK--NISFEVDCSYWDSDWSTSG--C-TNKV-TCLCNHLTSFA
Dmel_NP_651842	--FKVYFVRTKPRSVISLL--PLPFDSKCGYWN-TWSTEG--VESD-I-ECHTNHLTQFA
Dmel_NP_651845	--FKVYFVIKRPTSVISLG--KLPF-ANCGYWN-TWLSDG--ISGSHI-LCHADHLTQFT
Tcas_EFA07473	--ITVFFNNNKVSNIFGL--GPVSISETCVFWNGSWQNDT----NDLT-RCDFWHTTHFG
Hsap_ENSP00000296619	--FTEYFINLTLKNVLSLN--EVLYEPRCLLWN--WLSDSQFCV-TYV-ECACSHMSVYA
Cmil_SINCAMP00000019338	--FTEYFQKGALNDIFSLN--EITYENRCLLWN--WLSDGQFCV-TYV-ECACSHMSIYS

```
Drer_ENSDARG00000021137                  --FTEYFLKPALNGVFSLQ--EVVYDRRCLLWN--WLSDGQFCV-TYV-ECACSHLSIYT
Drer_ENSDARG00000089584                  -------------MVFSLQ--EVVYDRRCLLWN--WLSDGQFCV-TYV-ECACSHLSIYT
Pmar_ENSPMAG00000003119                  --FVEYYQQPVMAGVLSLG--EVTYDRRCLLWN--WLADSKYCV-SFV-ECACSHMSTYA
Spur_WHL22.718158                        --FIEYFYDLTLNKVMSLT--PVRYDSRCILYNRQWLTSF--CV-NFV-ECSCNHLSEYA
Skow_NP_001161550                        ------------------------------------------------------------
Bbel_057670F                             ------------------------------------------------------------
Adig_11959v108662                        --------QAVISDVFSVN--PITYNRRCVFWD--WSKEG--CK-KYV-ECQCDHLSVFA
Nvec_NVE21116                            --FIEYFT--VVNSVLSLE--PFTYNKRCVFWDSVWSTSG--CK--FV-ECQCTHMSNYA
Bbel_108220F                             --FIEYF-DSVVGQIISLD--PVKYDQRCVTWN--WQATG--CL-KY-------------
Cgig_10005042                            --VIEYFMQKLLSNVIS-V--PARFDKRCVYFD--WISPRGVCNGQFV-DCSCKHLTHYA
Ctel_219079                              --IVVYFTTPFLNDVLSLN--PVEYNKKCRYFD--WTSGNDVCTSIHV-DCECKHMSLYA
Hmag_XP_002158964                        --FLVLFIDKVLNNIMSLQ--KVIFKKRCVIWEGFWSPTH--CV-NFV-ECECDHMSEYA
Scil_184                                 --FVEYFTNAVLSNVLSLY--DVTYESRCLFWD--WSDST--CQADYI-ECRCKHLPDLA
Aque_2_1_38404_001                       --FIEYYK--LLGMVMVLN--PVTYSGTCLVWSGSWDQST--CAASYV-ECQCQLVGDYA
Sros_PTSG_07638                          --------NGLLQNVLSLN--PLVYEQQCVWWDGAWTSEG--CVSDHV-ECRCTHATAFA
Mlei_ML02596a                            --FTSYLF--ELDGIVGFK--RVLMENKCQFWDGVWSSEG--MIKTSI-DCMSDHLTNFA
Drer_ENSDARG00000091757                  --VIVFILNSRIVGVVV-Q--TVNINNECVFWNSVWDSYG--C-SNII-TCECNHTTSFS
Aque_2_1_28798_001                       --FSVFFIDGTVGTVLDLE--PIQLTPPCRFWDNEWSSDG--CVGN---ICSCDHLTSFA
Turt_05g00150                            --YIVYLLNDIVNSVVGLE--PIEINITCVFWDSQWSEFG--CVSNHT-LCRCNHLTNFA
Dmel_latrophilin_Cirl_NP_001137620       --FAAFILSDILNSVISLQ--PITLKTECVFWNHAWSANG--C-TNHS-VCSCNHLTNFA
Bbel_214760R                             --AFDHLLHEELNSVVTLT--KVEIRGQCVFIDSIYNPSG--CATDST-VCKCNHFSSYA
Hsap_ENSP00000359630                     --AVLYILNYVINSIIVL-----EINGTCVLWDGTWSTQG--CVTDHT-KCLCDRLSTFA
Drer_ENSDARG00000059832                  --MVLYMLNNAVNSVIAL-----EINGTCALWDGSWSSKG--CVTDHT-KCLCDRVSTFA
Cmil_SINCAMP00000015602                  --AVLYILKAALNSVLA----PMIVNGTCVVWDGSWDPKG--CVTKYT-KCLCNQLATFA
Nvec_NVE12353                            --NVIYFLKRDVDGVVSLT--PIRIQKRCVFWDGSWSSEG--CLSSAT-VCECDHMTSFS
Scil_50471                               -----------------------------AFYNRRWRTEG--CVPTVV-TCDCDHLTNFA
Dvir_Mthl5_XP_002053335.1                CGWPVL--DKLLDDKLR-EQSGV--KADPRFYD--------YCLISHAHICLANNWNDSN
Dmel_Mthl5                               CGWPIY--DKLLDDRLR-ERHGI--QSDPLYHD--------YCITSEANICLAIKWSDSN
Tcas_Mthl5                               CGWPIY--DRLLSNVLRI----------SLYHD--------YCLVDKARVCAPTHWTETK
Bbel_270220R                             --QIAYLLKENINAMVGLE--PLLAKYYCLTLDYRWVADG--CVKRTV-LCECRQTGIVT
Dmel_Mthl9                               --ELTY--NRTVDQIRDLN-KFV--DKKDNFWDRLWSTDE-YCLHNET-PLNC-------
Dvir_Mthl9_XP_002046453.1                --ELSY--NRSVAQILELN-KFV--DTKDTFWDRLWSTDE-YCLHNET-PLNC-------
Dmel_Mthl8                               --------SHEVESLQP-HMKAV---TKYVHWTHIFSKHY--CLGNEQ-PLACPEKLYFV
Dvir_Mthl8_XP_002046452.1                --------SHTVESLRACHMRAL---TKYLNWTHLFTKHY--CLGNNQ-PLACPEKLHFA
Dvir_Mthl14_XP_002060308.1               CPLDFY--DSVLNEVISL---EIPTNSSDQFFDESYTVEENYCLKS-------GHMLAFI
Dmel_Mthl14                              CPLNIY--DSLLSEIISL---EVETNSSDVFFDESYTVKEHYCLKS-------GHLFAFT

----P------SIT-GQTVV-MISSLICMVLTIAVYLFVKKLQNLHGKCFICYMVCLFMG
----P------SRT-GQTVV-MITSLICLVLTIAVYLCVKKLMNLEGKCFICYMMCLFFG
----SE---H-SRT-WKTVA-IVISLICIILTISVYLYVEKLRNLHGKCFICYLASLFLG
----SE---H-SRT-WKTVA-IVISLICIILTISVYLYVEKLRNLHGKCFICYLASLFLG
```

```
----ND-PPE-SQL-LYNIL-RLLSIICLLSTIIVYLFIPKLRNLHGCCFTCYMASLLIA
--------PP-TEK-TNAYI-QTVSIFCLAIIIVVYLYLPNFKSIHGKCCTCYFTCLTAS
----PS-MKR-LSN-ASIPV-KFSSVFFMVITIAAYLWLPKFRSLHGKCCNLYFICLAIT
----SS-TKS-TIT-LPDAL-RVISIFCMIITIATYLYFPKFRSVYDKCCICYFFCLAVS
---------K-SMP-AVPQV-GTISMVGCILTIAVYLYIKKLRNLLGKCFICYVFCKFVQ
---------H-ISP-GSLEI-LIITMICFVLTIAVYLYIKKLRNVTGKCIVCCIVSRFIQ
---------N-VMP-GIAQL-SVISVVGFILTLAVYLSVEKLRNLLGKCLICSLFSMFME
----DD-HST-VKI-INSYA-MMFSIPFMMLTIAVYLLIPELRNQHGKSLVCYLIGLSVG
----NE-NTT-VKI-INGFA-MLFSIPFMLLTIAVYLLIPELRNQHGKSL----------
----YE-PPP-TIM-LNTII-MLISAPFLYITILVYWLIPELWNLHTKCLICYLISLAVG
----YS-EPP-TMM-LNTKI-MMISAPFLFATILIYWLIPELWNLHTKCLISYLLSLAVG
----NE-ASL-SLQ-INNIA-MAVSVPFIILTIIVYVFIPELRNLHGKCLISYLCPLAVG
---------I-ESP-GAFEL-ASGSIICYIIIFGIYLFVKELRNDFGKCVMSCVFCLFLD
----AD-NQL-VLK-VFPYF-LFLSSLFLIATFVVYAMIPEIRNIHGVTIMCHVASLAVM
----EE-NSV-IQS-IYPYF-LFVSSLFLIATFVVHALLPELRNTHGVTVMCHVASMTVM
----TV-AAE-SSN-LLSVG-MIISMPFLLLTFIAYALLPD-RNLHMKALMFYVINLMFS
----TN-EEF-KYN-LYPVG-MLLSVPFLLLTFFVYACIPDLRNMHGKSLMCYVLGLSVG
----SK-DEI-RQK-SYNIG-VIVSIPFFFITFLVYAIIPELRNLYGKTLMCYVASLVVA
----NT-D-L-IYT-MYPVG-MLVSIPFLMCTFLVYALIPELRNLHGKSLMCHVSSLLTA
----NT-DEL-IYT-MYPVG-MLVSIPFLLVTFLVYALIPELRNLHGKSLMCHVSSLLTA
------------------------------------------------------------
-----------------------------LATFFVYALIPDLRNTHGKSLMCNVSCLIMG
----DV-PPW-VYI-LYPIC-MIISAMLLAITFLVYLFVPELHNLHGRTLMCYVASLFLA
----NV-PSW-LYT-LYPIC-MIISSMLLVLTFLVYLFVPELRNLHGRTLMCYVASLLLA
----DV-DPV-VAT-LHTLG-MITSAIFLVLTLIVYLIVPDLRNLHGRTLMCFVGTMLLA
----EA-NQL-AHS-IYPYL-LFISSGFLFLTFVVYAFLSEIFNAHGLIVMCYVASMAVL
----AE-DPH-NYL-LSAAC-MLISCVFILATLAVYAWLPELRNLYGKVLMAYLSCLLLA
--------DD-TNP-IYSIF-LFISSFFLVATFAVYALIPEIRNIHGVVIMCYVVSRAAA
AGASNG-QNL-QKA-VLTGG-ILVSIVFLSATLVAGFLLPAVHHLHWRCQICYVTCLLFG
--GP-E-GSL-QKA-VLDVG-ILISIVFLAATLVAGYMLPAVHHLHWRCQIYYVFCLLLG
----ES-HDI-RFI-IYPIS-LALSAVFLAATLAAGAILPASHHLHWRCQTNHVACLLVG
----YK-TNA-IGT-MFLVA-YAISALSLVLTFILSIVISELNMRNNYILLCHLVVMTLA
----GE-DAK-GAY-LFGCL-QLFATVFLVATLLVYAILPKLRNLHGVVVMCYLASMTAT
----GE-DSK-GAY-LFGCL-QLFATVFLVATLLVYAILPKMRNLHGVIVMCYLASMTVT
----GE-DAK-GAY-LFGCL-NLFATVFLVATLLVYAILPKLRNLHGVVVMCYLASMTVT
---DKF-SSA-QTV-VSVIG-QVISIVCLSVHLLVYSMFRKLRNLAGLNIMSLSGCLLIA
---SKF-SST-QTI-VSVIG-QVISIVCLSLHLLVYSMFRKLRNLPGLNIMSLSACLLIA
----KF-SVI-QTA-VSVIG-QILSIIGLVIQLTVYSILPKLRNLAGLNLMSLSASLLVA
---SRF-DAA-QGY-ITVVG-NTVSVICLLVHLLVYCSYSTLRNRPGKCIMCLSASLMFS
---SKYASTG-QHY-MSDIC-LAISVVCLAFHIIVHMILPKLRNLPGKNLLSLSCALFMA
-------SYA-QRC-LSNVC-LTISVICLAFHIAIHIALPKLRNVPAKNLLALSCTLFLG
```

```
TMT-QY-SIS-QTI-LTAVG-SCLSILFLLLSLATYCVFHSLRNIPGKTIMNLIIALLVG
S--SDY-PQF-YTF-LTVFL-TLISLACLIGTFVTYAKFEELRTVPGLCIMSLTVSLFLA
---QNL-PKG-LDI-MTIVG-TVLSIISVLAVITTYTVFPKLRNMAGKSVLALSVALLMV
-------------------------------------------SGKLNASLCLWLLLA
---QRF-TSA-QSI-LSTIG-ISLSIIALIVTFVTYLQFNLLRKTSNTLIMSLCITLIFA
---TQF-SKG-QSI-TSFVG-CLLSIAGLVFTFVTYAKFGSLRKICSQVVMSICVSLTLA
---NQF-SKG-QSI-TSFVG-CLLSIAGLVFTFVTYAKFGSLRKICSQLVMSICASLILA
TVLGKT-VNV-QLI-YSHVL-LAISTLCLILLIVTYLMFPELRNFQGCAILSFALSLLIS
----PD-DTA-LAW-LTFFC-MLLSIATLVLFLVTYSLFSELRTIPGVNLMNLAVSILLA
----AD-DPA-LSW-LTLMC-MLLSIAGLVFFLVTYLLFSELRTIPGVNLMNLALSTLLA
T----G-SEV-LRM-VTFVC-LLISVIALGFVLVTYSLFSELRTTPGKNLMNLSTAILLS
----------------------------------------------------------
PVA----LIV-ESY-LTLCL-MSVSILAMMATIVTYLIFPELRNLAGLAVMNLCLMTSGF
----TG-DRI-RFK-MYPIG-LLISCCFYALTLIVYISIAKLRNLPGKILICLVSSLFAA
---------------XYPVG-LLISCCFYAITLLVYTSIEKLRNLPGKILICLVSSLLFA
---NGP-R-I-LLWSITLGV-NVMSIVSLLLTISIYIKVKTLQTLAGKNNMILFVSLTIT
A--KVM-RTI-LIV-VTYIS-TILSLLGLTLTFLTFCILPSLRTLPGKNMMCYTLSLFAA
------------MW-VTYLS-TILSMFALFVTFVIYCILPALRTIPGKNLMCFAFSLFFA
Y--NKK-DEI-LRW-VTLST-TLLSLICLFMTFIVYCTISSLQTLPGKNLMVLVLNLFLA
------------------------------------MVVPGLRTLPGLNTMSTTFSLLCM
ID-----SRT-LMI-IGHVL-FAISSVCLFATLITYVAFSTLRNRQGVSIMNFIVALFLG
---YTW-DAN-KSW-LTLGL-VTVSVVAVTGFVFHTYRSGQWKKVPEKLKVQMMTCMAAS
---YAW-DAT-KSW-LTLGL-VSVSDIAVAGFVFYTYKSGQWKKVPEKLKVQVMTCMAVA
---YS--DPV-QHW-LTLGL-VIVSDVAVSGFVVHTYRSGQWKKIPEKLKVQMMTCMAVA
---YS--DPV-QHW-LTLGL-VIVSDFAVSGFVAHTYRSGQWKKLPEKLKVQMMTCMAVA
---YKD-LYS-QTL-LTTTM-LSLSIASGIGFIAHGVYYRKFSSTPDRLKLQLVVMLTLA
---TTE-DNV-SGI-LNIAL-TALSAVGGIVYLALCVVTREYHSIPGRLKMQLVITLTAA
SNFTEG--TP-QEI-LTLAL-SSLSFVSMLVFIAAAVV-KKMRKIPDILKLNLTCSLLVA
----ST-GNT-IYI-VHAVL-ELISVFFIVITIFVYLQLDDLLDLQGKSILHSIAGLALS
G--VTA--TA-DRV-VTFVA-MVVSLTTLAATIVTYLLFSSLRTLPGLTILNLSTSLFLS
LLVDSQ-ATA-ISV-LTYIG-CIGSIIGAAIYLVTFGVLSTLRSGPTILTMHLCAALIGA
VLLDSQ--AG-LEV-LTYVG-CALSMIGAVIYILTFSILRSLATGPTYLVMNLCVAIFFT
VLLDGD-AAA-LEI-ISYVG-SAFSMVGCTIFIVTFSLLRQLATGPTKLVMNLCVAIFFA
VLFDSS-DTV-LVV-FTYVG-CILSMIGCFILVVTFLLLPKLRTGPIVLIMHLSVAIFLV
LIFDAD-GGA-LEV-LTYIA-CVSSMLCCLLFVIVFAYLEKLRSGPILLTMQLCVVLFLT
IFRKDQ-KQT-KYV-YTSIA-MIISCVFLVLTILYYCFSNE-KQLFGKTLISYCFTYLLT
VLFGAN-SDA-VTY-AAYAG-CALSILGSLATFVTFALFPHLRVGPTKLVMQLCVALIAT
ILFDES-KLA-LRY-LSAIG-CALSVAGCVFTLIVFMLLPKLRTGPVVLVMGLCVAILAV
LFFSIS-ADI-LSN-ITYIG-CALSIFGTVVTFMTFVLLKTLRTFAMKLVMHLCVAVFGI
LFFSVS-AEI-LSN-ITYVG-CALSIFGTVVTFVTFVLLRTLRSFAMKLVMHLCVAIFGI
VLFDGS-SEV-LST-ITYVG-CALSLAGVLLTFGTFLAFSQLRT-AIRMVMHLCVAIFAV
```

```
VLLDSD-DQI-LTF-ITYIG-SGISALFLSVTLVTYIFFEKLRRYPSKILINLCTALLFL
VLLDAQ-MMA-LTF-ITYIG-CGLSSIFLSVTLVTYIAFEKIRRYPSKILIQLCAALLLL
VLMDVN-EQI-LAL-ITYTG-CGISSIFLGVAVVTYIAFHKLRKYPAKILINLCTALLML
ILLGLN-TKL-LSF-ITYIG-CGASSIFLGIVLLTYLIFEELRRYPSKILLNLCTALLML
ILMDKN-NRV-LTF-ITYIG-CGISAIFSAATLLTYIAFEKLRRYPSKILMNLSTSLLFL
VLMDRN-TKV-LTF-ISYIG-CGISAIFSAATLLTYVAFEKLRRYPSKILMNLSTALLFL
VLMDMQ-ATI-LTF-ISYIG-CGVSAIFLSVTLLTYLSFDKIRRIPSKILIHLCFALLFL
VLLDKD-EWV-LTI-ISYVG-CGISSIFLGVTLLTYLAFEKLRRYPSKILINLCMALLGL
ILLDLD-LLI-LTY-ITYIG-CGISAIFLSVTLLTYLAFGKLRKIPSKILIHLCFALLLL
VLLDEV-LVS-LTY-ITQIG-CGISAIFSALNVILYIPLRNFKSHATVIHINLSAALFLL
VLFDKN-KIA-LSI-VSYVG-CALSIIGLILTIITYSIFRCLNRRSGKILLCLCVAMLLM
VMLDSH-EKA-LTY-ISYIG-CAISIFGLAFTIVTYSMFRSLNRRSGKILLNMCVSMLLM
ILLDIH-EEI-LTY-ITYIG-CAISMVGLILTIITYSLFKCLHGKSGKILLNLCIAMLLM
ILLDYH-EYI-LRL-ISYVG-SGLSILGLSITVLLYSLFRNLRRRGGKILLNLCISLLML
LLMDGH-QKA-LSI-VSYIG-CGISLLCMLLTLITFLAFRRLRKNPTKILMNLCFAIFMS
LLMDSH-QKA-LSI-VSYIG-CGISLFCMLLTLITFLAFRRLRKNPTKILMNLCFAIFMS
LLMDGH-KKA-LSI-VSYIG-CGISLFCMLLTLITFLAFRRLRKNPTKILMNLCFAIFMS
LLMDGH-QKA-LSI-VSYIG-CGISLLCMLLTLITFLAFRRLRKNPTKILMNLCFAIFMS
VLMDGH-QNA-LSI-VSYIG-CGISMLCMLLTLVTFLAFRRLRKNPAKILMNLCFAILMS
VLLDTH-ELI-LRI-LSYIG-CGLSVTGLLITLLSYIMFGKIKRAPAKILVCLCVSLIAL
LLMDEN-QKA-LSI-ISMIG-CAVSSAGLLFALITFLLFRTLRRNPTKILINLCVALLLV
VLFSEH-AMA-LSI-ISYVG-CAISLAGLSLTLITYSMFRNLRKNQQPILMSLCISLMLL
VLMDLH-SFA-LDL-ISKIG-IALSITGLVLTLITYLVFKQLRQRPQHILINLCVALLAT
VLMDLH-SFA-LDL-ISKIG-IALSITGLVLTLITYLVFKQLRQRPQHILINLCVALLAT
VLMDLH-SFA-LDL-ISKIG-IALSITGLVLTLITYLVFKQLRQRPQHILINLCVALLAT
VLMDLH-NFL-LDL-ISKIG-IVVSITGLTLTLISYLIFKQLRKRPQHILTNLCIALLAT
VLMDLH-SFL-LDL-ISKIG-IVVSITGLTLTLISYLIFKQLRKRPQHILTNLCIALLAT
VLVDLH-SFT-LDL-ISKIG-IALSIPSLALTLITYLAFKKLRQRPHRILINLCLALLAT
VLMDLQ-SFA-LDL-ISTIG-IALSITGLSLTLLSYLIFKQLRQRPQHILANLCIALLAT
AFFDVH-ERP-LQV-ITIVG-CAVSIVGLVLTLLSFIITRKYRRTGVHVLINLCLTLLAI
VFFDAH-ERL-LEI-ITTVG-CVVSITGLVLTLLSFIVTMKYRRVGAHVLINLCVALLAI
MLFDDH-GKP-LEA-ISIVG-CVVSIICLVLILLAFIVTRK-RRRTGHILINLCVALLAT
IIFDDH-EKP-LEI-ITIVG-CVVSFVCLVLTLFSFIVTRK-RRRTGHAFINLCVALLAT
```

Text file S3: Uncollapsed PhyloBayes tree.

(Adig_11188v107943:0.38203,Nvec_NVE16100:0.352589,(Nvec_NVE16098:0.318055,((Spur_WHL22.6202:0.464519,Skow_XP_00
2733056:0.317153)0.99:0.131777,(((Smar_011200:0.266059,Dpul_EFX83073:0.430216)0.98:0.13624,(Tcas_latrophilin_Cirl_XP_00
8200380:0.243802,(Turt_05g00150:1.37653,Dmel_latrophilin_Cirl_NP_001137620:1.20766)0.64:0.240435)0.56:0.100927)0.75:0.16
6746,Ctel_226868:0.411482)0.79:0.05546,(gi_XP_009668681:0.998191,Cmil_SINCAMP00000011725:0.595653)1:0.432334,(Cint_
ENSCINP00000004251:0.403937,((((Hsap_ENSP00000340688:0.119438,Drer_ENSDARG00000089292:0.06378)1:0.076534,(Drer
_ENSDARG00000069356:0.214374,(Hsap_ENSP00000359752:0.102077,Cmil_SINCAMP00000012795:0.069084)0.93:0.037525)0.
92:0.036572)1:0.082661,((Hsap_ENSP00000420931:0.014089,Drer_ENSDARG00000061121:0.067681)0.57:0.016955,Cmil_SINCA
MP00000005886:0.07139)1:0.170616)0.98:0.089616,Bbel_191840R:0.371369)1:0.139432)1:0.152475,((Smar_006024:0.890547,Sk
ow_gi_XP_006820674:0.357457)0.68:0.073038,Cint_gi_XP_002121696:0.814754,(Bbel_166310F:0.250848,(Bbel_163260R:0.4362
56,Bbel_161400F:0.2952)1:0.15479)1:0.384741)0.81:0.1176,(Hrob_P80944:0.82719,Dpul_EFX79819:1.08394,((Bbel_270220R:1.65
046,Bbel_214760R:2.08474)1:0.485387,(Skow_XP_002741140:0.34684,(((Hsap_ENSP00000262738:0.323718,((Drer_ENSDARG0
0000069185:0.041927,Drer_ENSDARG00000058259:0.164111)0.88:0.050033,Cmil_SINCAMP00000020331:0.131121)0.66:0.0494
5)0.6:0.073698,((Hsap_ENSP00000271332:0.242237,Drer_ENSDARG00000019726:0.327601)0.99:0.073052,Cmil_SINCAMP0000
0017405:0.142538)1:0.197079)1:0.481021,(Nvec_NVE25349:0.636826,(Smar_001588:0.707908,Cgig_10006474:0.807782)1:0.204
195,Bbel_010420R:0.549548)0.52:0.068808)0.5:0.088575)1:0.519074,(((Nvec_NVE22429:1.06938,Adig_8886v105801:1.13226)0.9
1:0.233636,(((Sros_PTSG_09543:0.039837,Sros_PTSG_09542:0.073235)1:0.697601,Sros_PTSG_06041:0.682277)1:0.837371,(Sci
l_24201:1.41038,(Scil_50471:1.1269,(Scil_7737:0.003169,Scil_14656:0.003011)1:1.32234)0.74:0.290266,((Scil_289:0.003339,Scil_
227:0.003078)1:1.04806,((Scil_9494:0.699057,Scil_47986:0.933037,((Scil_68080:0.003772,Scil_49553:0.003765)1:0.264215,(Scil_
70266:0.464902,(Scil_63570:0.253729,Scil_41456:0.315164)0.94:0.098619)1:0.14851)1:0.582947,Scil_27094:0.356129)0.78:0.225
461,Scil_14083:1.26842)1:0.407913)0.91:0.254553,Ocar_g3634_t1:0.883585,Mlei_ML02596a:1.66158,(Tcas_EFA07473:0.887595,(
Dmel_NP_651845:0.716168,Dmel_NP_651842:0.270138)1:0.643169)1:1.08569,(((Smar_003150:0.33108,Dpul_EFX80180:0.71594
5)1:0.276945,(Lgig_74363:0.371982,Cgig_10006507:0.353544)0.87:0.100353)1:0.582237,((Skow_XP_002735776:0.05491,Skow_X
P_002734798:0.039775,Skow_XP_002733570:0.129774,
(Skow_XP_002733572:0.027088,Skow_XP_002733569:0.048362)0.63:0.015294)1:0.477859,((((Hsap_ENSP00000369198:0.23467
9,(Drer_ENSDARG00000088937:0.224411,Drer_ENSDARG00000088137:0.195734)1:0.161306)0.89:0.087803,Cmil_SINCAMP000
00022659:0.260378,(((Hsap_ENSP00000356581:0.233951,Drer_ENSDARG00000054137:0.114306)1:0.176925,(Drer_ENSDARG0
0000087813:0.469287,(Hsap_ENSP00000359686:0.347636,Cmil_SINCAMP00000023038:0.377574)0.99:0.107157)0.97:0.07065)0.
51:0.063524,Cmil_SINCAMP00000004297:1.13119)0.5:0.060573)1:0.472545,Cint_ENSCINP00000004627:0.749813)1:0.188555,Bb
el_144770R:0.683833)0.59:0.073068)1:0.175339)0.63:0.106079,Aque_2_1_20823_001:0.979025,((Cowc_CAOG_05333:1.06343,((

Cowc_CAOG_03584:0.0784,Cowc_CAOG_03451:0.193357)1:0.439246,(Mvib_comp15494_c4_seq1_fr4:1.25869,Cowc_CAOG_03
413:0.448406)0.88:0.134895)1:0.301511)0.91:0.129018,((Mvib_comp15911_c1_seq2_fr4:0.627525,Mvib_comp12816_c1_seq1_fr6:
0.557495)1:1.11186,((((Mvib_comp15999_c0_seq1_fr4:0.191758,Mvib_comp15986_c0_seq2_fr4:0.179919)1:0.431245,Mvib_comp1
5877_c0_seq1_fr6:0.766444)0.65:0.093144,(Mvib_comp15861_c0_seq1_fr4:0.596341,Mvib_comp11351_c1_seq1_fr6:0.563633)0.6
6:0.124008)1:0.390622,(Cowc_CAOG_00812:0.774362,Cowc_CAOG_00386:0.80006)1:0.35149,((Bflo_gi_XP_002608881:1.12267,
(((Bbel_293770F:0.056297,(Bbel_192410R:0.084055,(Bbel_145670F:0.03752,Bbel_134080R:0.041735)1:0.128777)1:0.107823)1:0.
974427,Bbel_132690R:0.98575)0.54:0.273116,Bbel_069800F:1.15333)1:0.58083)1:0.627169,((Skow_XP_006818657:0.759219,Lgi
g_75877:1.20908,(((Spur_gi_XP_798567:0.033859,Spur_gi_XP_011675990:0.04059)1:0.382536,Spur_XP_011660493:0.435709)1:
0.65461,(Spur_XP_011662057:1.37834,(Spur_XP_011679633.1:1.15258,Spur_XP_003723996:0.985681)1:0.564064)0.7:0.205013,
Dpul_EFX79407.1:1.46011)0.56:0.148373)0.5:0.138752,(Skow_XP_006821924.1:0.913522,(Cgig_10025895:1.05428,Cgig_100210
40:1.2998,Cgig_10006654:1.03149,(Cgig_10015721:0.429286,Cgig_10001367:0.292654)1:0.607257)1:0.9764)0.67:0.154264,((Nve
c_XP_001628572:0.52113,(Nvec_XP_001640473:0.193725,Nvec_XP_001636770:0.192942)1:0.428384)1:0.807534,Bflo_XP_0026
10765.1:0.842426)0.71:0.183389,((Gdib_GASB01015634:0.680047,Dpul_EFX67288:1.19293)0.82:0.325219,(Ctel_63867:0.438719,
(Ctel_216805:0.067327,Ctel_208644:0.079077)1:0.424852)1:0.310596,((Dpul_EFX73786.1:0.453695,Dpul_EFX73784.1:0.382963)1
:0.513565,((Tcas_Mthl5:0.418288,(Dvir_Mthl5_XP_002053335.1:0.096514,Dmel_Mthl5:0.090068)1:0.310102)1:2.05788,((Tcas_Mthl
15:1.43509,(Dvir_Mthl14_XP_002060308.1:0.22382,Dmel_Mthl14:0.129603)1:2.57613)0.79:0.361462,((Tcas_Mthl16:1.7507,(Pxyl_
XP_011552150:0.891452,(Dvir_XP_002060099.1:0.148071,Dmel_Mthl15:0.17475)1:1.12185)0.68:0.15895)0.76:0.147343,((((Dpul_
EFX89687:0.713868,
(Methuselah_Dpul_EFX89685:0.199071,Dpul_EFX89686:0.404917)1:0.129166)0.73:0.087919,((Dpul_EFX83097:0.099473,Dpul_EF
X83094:0.056869,Dpul_EFX83013:0.065074)1:0.967781,Dpul_EFX74305:0.833033)1:0.227749)1:0.524427,(Tcas_Mthl1:0.44025,(
Dvir_Mthl1_XP_002059559.1:0.157153,Dmel_Mthl1:0.151455)1:0.366282)1:1.09811,(Smar_012256:1.59296,(Asia_JW965784:0.59
0461,(Asia_JW969407:0.063247,Asia_JW948170_JW948387:0.151798)1:0.229775)1:0.517439)0.6:0.191429)0.82:0.12189,((Tcas_
Mthlsc_XP_008190998:0.897218,(Mrot_XP_003703117:0.601038,(Cbol_GAYO01128358:0.45716,(Cbol_GAYO01127990:0.306647
,(Esup_GAVW01003911:0.09544,Cbol_GAYO01008982:0.070336)0.63:0.0696)1:0.184543)1:0.19282)0.65:0.068431)0.57:0.103049
,((((Dvir_Mthl9_XP_002046453.1:0.176105,Dmel_Mthl9:0.06557)1:1.65018,(Dvir_Mthl8_XP_002046452.1:0.20647,Dmel_Mthl8:0.11
2982)1:1.53199)1:1.43648,((Dvir_Mthl10_XP_002046455.1:0.076475,Dmel_Mthl10:0.233744)1:0.41513,(Mdom_XP_005175058.1_
Mth2like:0.487745,((((Dmel_Mthl4:0.191781,Dmel_Mthl3:0.044388)1:0.335834,(Dmel_methuselah:0.135431,Dmel_Mthl2:0.412066)
1:0.130677)1:0.217731,(Dmel_Mthl6:0.627824,(Dmel_Mthl7:0.589327,(Dmel_Mthl13:1.12468,Dmel_Mthl12:0.557152)0.94:0.143684
)1:0.355683)1:0.539008)0.98:0.124134,(Dvir_XP_002046457.1:0.533524,((Dvir_Mthl11_XP_002060419.1:1.07459,Dvir_Mthl11_XP
_002053031.1:0.456802)0.97:0.225641,Dmel_Mthl11:0.813099)1:0.456966)0.5:0.089277)1:0.542267,(Ceratitis:0.216204,Bactrocera
_dorsalis__XP_011201382.1_Mth2like:0.297836)1:0.429608)0.94:0.189105)0.99:0.376171)0.99:0.414427,Agam_XP_316287.2:0.55

267)0.92:0.340696)0.84:0.149012)0.94:0.221892)0.99:0.317757)0.98:0.550129)0.87:0.358803)0.68:0.2216)1:0.281029)0.67:0.1990
52)1:1.19161)0.6:0.103386)0.68:0.122086)0.86:0.31229,(Skow_XP_002740291:0.560809,(Cint_gi_XP_002129016:1.26907,(Hsap_
ENSP00000273352:1.40943,(Cint_gi_XP_009858460:0.657532,(Cint_gi_XP_009860041:0.341052,Cint_gi_XP_009858263:0.55615
2)0.69:0.210339,Cint_gi_XP_004225576:1.49661,Cint_gi_XP_002124051:1.20295)1:0.490414)0.75:0.28468)0.76:0.160285,(((Bbel_
238310R:0.111798,(Bbel_230420R:0.014339,Bbel_130640F:0.014863)1:0.058506)1:0.064752,(Bflo_gi_XP_002598501:0.033469,(B
bel_156760R:0.115943,(Bbel_063250R:0.003394,Bbel_062450F:0.003392)0.99:0.019989)0.97:0.021944)0.93:0.039333)1:0.351675
,((Bbel_299040F:0.120457,(Bbel_121130R:0.098709,(Bbel_281940F:0.044305,Bbel_079450F:0.079092)1:0.046225)0.87:0.024678)
1:0.186971,(Bbel_204650R:0.240571,Bbel_060890R:0.173255)0.96:0.094266)1:0.670229)0.74:0.089041,(Spur_WHL22.73425:0.77
4199,(Spur_WHL22.3487:0.181871,Spur_WHL22.3020:0.454754)1:0.594225,((Spur_WHL22.184504:0.086497,((Spur_WHL22.7545
06:0.036915,Spur_WHL22.383522:0.06833)0.99:0.021816,Spur_WHL22.147647:0.049705)0.64:0.01771)1:0.191968,(Spur_WHL22.
264380:0.034841,
(Spur_WHL22.230519:0.023571,(Spur_WHL22.226322:0.040464,Spur_WHL22.138572:0.062559)0.84:0.021285)1:0.048536)0.99:0.
124255)1:0.917258,Aque_2_1_28798_001:1.38136)0.55:0.17479,(Hmag_XP_002158889:1.13677,(Nvec_NVE23935:0.553577,Adig
_17884v114019:0.443406)1:0.451818)0.69:0.110455,(Nvec_NVE6184:0.289524,Adig_16756v113082:0.301043)1:0.744723)0.55:0.
147108,(Sros_PTSG_07638:2.13076,(Hmag_XP_002158964:0.536562,((Bbel_108220F:0.394272,(((Pmar_ENSPMAG00000003119
:0.396796,(Hsap_ENSP00000296619:0.227292,((Drer_ENSDARG00000089584:0.03187,Drer_ENSDARG00000021137:0.023757)1
:0.259387,Cmil_SINCAMP00000019338:0.157187)0.61:0.050895)1:0.200219)1:0.306065,Bbel_057670F:0.520317)0.5:0.098507,((S
kow_NP_001161550:0.224569,(Ctel_219079:0.552041,Cgig_10005042:0.406679)1:0.228074)0.97:0.123161,(Spur_WHL22.718158:
0.367945,(Scil_184:0.921543,Aque_2_1_38404_001:1.69483)1:0.533775)0.54:0.075137)0.85:0.101204)0.74:0.121566)0.81:0.1487
45,(Nvec_NVE21116:0.153138,Adig_11959v108662:0.539293)1:0.189602)0.73:0.132803)1:0.83472)1:0.853774)0.95:0.138216)0.99
:0.237238)0.81:0.135427,((Nvec_NVE12353:1.61139,((Hsap_ENSP00000359630:0.06604,Drer_ENSDARG00000059832:0.037602)
0.54:0.0314,Cmil_SINCAMP00000015602:0.269226)1:1.03245)0.88:0.276044,((Scil_13054:0.902532,((Aque_2_1_39650_001:0.917
97,Aque_2_1_39648_001:0.495461)0.61:0.113244,Aque_2_1_37513_001:1.01306)1:0.412541)0.75:0.201168,Aque_2_1_37130_00
1:1.36028)0.71:0.148462,(Nvec_NVE13836:0.690338,((Lgig_154761:0.462063,Cint_gi_XP_009862007:0.426538)1:0.608948,Bbel_
260260F:0.591848)0.84:0.16413,(Spur_WHL22.392553:1.42094,(Nvec_NVE10940:0.816218,Adig_2760v120555:0.681778)0.93:0.2
66982)0.53:0.138518)0.97:0.13346,(Nvec_NVE18487:0.795835,((Skow_XP_002740941:0.533952,Skow_XP_002734724:0.569733)
1:0.325542,Bbel_292050F:0.839336)0.99:0.168044,((Tadh_XP_002118234:0.40373,Tadh_XP_002111670:0.269137)1:1.04787,Adi
g_19406v115301:0.732194)0.88:0.155714,(Nvec_NVE9578:0.639312,Nvec_NVE16489:0.692529,((Tadh_XP_002113682:0.806088,
(Tadh_XP_002117818:0.876415,Tadh_XP_002113325:0.739448)0.66:0.163276)0.88:0.172057,(Spur_WHL22.71613:0.484663,Drer
_ENSDARG00000091757:1.85473)1:0.520943)0.84:0.196836,(Skow_XP_002735837:0.55567,Adig_11540v108276:0.607591)0.86:
0.106375)0.55:0.137179)0.51:0.069307)0.85:0.044251)0.74:0.063483)1:0.152946)0.73:0.122383)1:0.484184)1:0.218708);

Text file S4: Uncollapsed maximum likelihood tree.

(Bactrocera_dorsalis_|XP_011201382.1_Mth2like:0.22366025285055277094,((Mdom_XP_005175058.1_Mth2like:0.3907034976591
2693041,((Dvir_Mthl10_XP_002046455.1:0.07530312075506233327,Dmel_Mthl10:0.17982805289613479349)100:0.292871261865
67402630,((Agam_XP_316287.2:0.44064649424258717225,((Tcas_Mthlsc_XP_008190998:0.69643436668952751756,(Mrot_XP_0
03703117:0.49596917567875997701,((Cbol_GAYO01127990:0.23166991178903578730,(Esup_GAVW01003911:0.083630080756
98077914,Cbol_GAYO01008982:0.06295780944086232234)72:0.04882495841817881960)95:0.12909596173991719215,Cbol_GA
YO01128358:0.36684576289452841547)90:0.12772885297897412782)57:0.07087363498387838634)34:0.087401566783615353
14,(((Tcas_Mthl15:1.16688697420293108209,(((Tcas_Mthl5:0.27659215506111073379,(Dmel_Mthl5:0.07389784834801475799,Dvir
_Mthl5_XP_002053335.1:0.08160416616988659666)99:0.28811635488356696078)100:0.98615340175377674825,(Dmel_Mthl14:0
.09288752976670808237,Dvir_Mthl14_XP_002060308.1:0.19644548687862076197)100:1.57480495372817852484)24:0.29088311
705738090218,((((Ctel_208644:0.06658447054911249829,Ctel_216805:0.05398685318883839762)100:0.30044802506317008772,
Ctel_63867:0.34730625558085964633)95:0.20122592302642958173,((Dpul_EFX73784.1:0.34728255878398978096,Dpul_EFX737
86.1:0.30430967373047967950)100:0.39565304557643404815,(Dpul_EFX67288:0.80434941089052425234,Gdib_GASB01015634
:0.47615163660351467190)62:0.20017125440521482682)16:0.06681340043995985523)40:0.08232275178656112136,((Skow_XP
_006818657:0.63809337852250602729,(Dpul_EFX79407.1:1.00918440561587874349,(((Spur_XP_011679633.1:0.8408615521476
7166477,Spur_XP_003723996:0.71018598446745118125)85:0.28816325970593070771,Spur_XP_011662057:0.951080440024729
80072)37:0.12155597329319674238,(Lgig_75877:0.82491406331637473492,(Spur_XP_011660493:0.33875751971500739357,(Sp
ur_gi_XP_798567:0.03243872025597642483,Spur_gi_XP_011675990:0.02554937613642669150)100:0.28859013117975518270)1
00:0.39962804567538351419)28:0.09027210840869999420)17:0.08395402209480962163)8:0.07874739753106575757)8:0.06671
560048294183920,(((Skow_XP_006821924.1:0.68041179238887250502,Bflo_XP_002610765.1:0.57467358281820379862)24:0.10
577169940180172758,(((Bbel_069800F:0.74138361955282794380,(Bbel_132690R:0.77046355212869099827,(Bbel_293770F:0.05
921182944546574817,(Bbel_192410R:0.06836811717820284739,(Bbel_145670F:0.02996352762333706621,Bbel_134080R:0.033
55120500885092061)95:0.10674650027967691468)81:0.07455220108159338332)100:0.65873100586727628691)52:0.190626699
34185627607)94:0.27992895617649293794,Bflo_gi_XP_002608881:0.80027181334994290651)100:0.36615419765680323749,(Nv
ec_XP_001628572:0.41443130507400777196,(Nvec_XP_001636770:0.15041775302787885327,Nvec_XP_001640473:0.16158943
542078740840)100:0.29789588539067440864)100:0.46253498254108088572)35:0.11557830544869418521)
3:0.05202936734007942987,(((Cgig_10025895:0.78203155350518882560,((Cgig_10001367:0.20224807798654159674,Cgig_1001
5721:0.34757067725008955472)100:0.43809738255626390346,Cgig_10006654:0.73452724525374635256)29:0.03019868664140
478928)51:0.13035368539663794163,Cgig_10021040:0.84912739877808196542)99:0.52918813576040957258,((((Cowc_CAOG_

00812:0.55679157645381593955,Cowc_CAOG_00386:0.54360247159106678261)100:0.21602024595815688679,((Mvib_comp159
11_c1_seq2_fr4:0.44487412254801450562,Mvib_comp12816_c1_seq1_fr6:0.45088879938775250356)100:0.65938179303052002
478,(Cowc_CAOG_05333:0.70745409567182848676,((Cowc_CAOG_03451:0.14556450267769055484,Cowc_CAOG_03584:0.079
41842387968482686)100:0.30282924408432831953,(Mvib_comp15494_c4_seq1_fr4:0.86010391701721222724,Cowc_CAOG_03
413:0.38619466820163234066)40:0.07245547724808996948)95:0.19613415282712984289)48:0.05184227042282175957)16:0.07
563635353709738385)17:0.09115543469156960066,(((Mvib_comp15999_c0_seq1_fr4:0.15697635102042969968,Mvib_comp1598
6_c0_seq2_fr4:0.13912757497261693529)100:0.30677806315263411729,Mvib_comp15877_c0_seq1_fr6:0.537482411709316987
29)43:0.04953558163387781349,(Mvib_comp11351_c1_seq1_fr6:0.43311197859592592829,Mvib_comp15861_c0_seq1_fr4:0.427
39393914292539423)44:0.09662563775745038053)99:0.28092041174462861752)37:0.11056920219207203027,(Scil_24201:0.931
73765004571706960,((Mlei_ML02596a:1.13925408211330192465,((((((((Smar_006024:0.64991327308379009953,((Hrob_P80944:
0.60787845877634172354,Dpul_EFX79819:0.66187208614286452413)12:0.11742504150029003940,((((Ctel_226868:0.339934783
09676306015,((Dpul_EFX83073:0.33032479624974026011,Smar_011200:0.21718658803797116774)80:0.1004435805232526263
1,(Tcas_latrophilin_Cirl_XP_008200380:0.19900514447649991778,(Turt_05g00150:0.88962560633118470399,Dmel_latrophilin_Cir
l_NP_001137620:0.75087877326887852991)25:0.20807006759139268226)8:0.07207404005853855489)16:0.13591011810000952
487)8:0.06815612224763693827,((Spur_WHL22.6202:0.36425830698725780721,Skow_XP_002733056:0.24066614798364799332
)86:0.10083125878568410805,(((Cmil_SINCAMP00000011725:0.40299579481501462963,gi_XP_009668681:0.7777790691020014
5241)97:0.26615782975730489790,((Adig_11188v107943:0.28992592526834626288,Nvec_NVE16100:0.29278323247626497627)
99:0.15939630835149407040,Nvec_NVE16098:0.24762901092825523319)99:0.31568185426062178589)34:0.0988058970729747
8264,(Cint_ENSCINP00000004251:0.30616813787738195174,(((Cmil_SINCAMP00000005886:0.05634314179586837751,(Hsap_E
NSP00000420931:0.01113009198551328030,Drer_ENSDARG00000061121:0.06006606832450395173)52:0.01178728093861957
683)100:0.13789272240680883197,((Hsap_ENSP00000340688:0.09782513819313326531,Drer_ENSDARG00000089292:0.05568
658984394054406)100:0.06228025248103209732,
((Cmil_SINCAMP00000012795:0.06188248970875980665,Hsap_ENSP00000359752:0.08205575776889613426)61:0.0287951467
7972824441,Drer_ENSDARG00000069356:0.18500485493561394379)62:0.02478232154818493793)97:0.0715093917338004009
3)78:0.07114725586336204111,Bbel_191840R:0.29712779938978794991)86:0.11561646624441165865)78:0.1016368804556193
8833)16:0.07176645103555578942)12:0.02523204103825313349)5:0.06235129266439567997,(Cint_gi_XP_002121696:0.6400707
7838993564622,Skow_gi_XP_006820674:0.29962795293021043941)20:0.06004220459704064788)3:0.06928790891656083639,(
Bbel_166310F:0.23605665937170303148,(Bbel_163260R:0.33527035867080340337,Bbel_161400F:0.23872495708025626149)67:
0.08960169994952318917)100:0.26722882238529488408)8:0.06242654455456167484)17:0.04728977946904428659)50:0.077110
38162721466083,((((Cmil_SINCAMP00000020331:0.12087331661628503143,(Drer_ENSDARG00000069185:0.030391366965337
72941,Drer_ENSDARG00000058259:0.14379103368331333512)40:0.04065646587674161955)34:0.04355270041123236041,Hsap

_ENSP00000262738:0.26223328460647554738)33:0.06374698652760749096,(Cmil_SINCAMP00000017405:0.11694455455614104211,(Hsap_ENSP00000271332:0.18935328918155630573,Drer_ENSDARG00000019726:0.25985351678601958936)84:0.06202004331265913856)100:0.13661388459824733155)99:0.33771947397386242740,((((Cgig_10006474:0.60477696645695400157,Smar_001588:0.53773948578661201481)83:0.16477506467215974340,Bbel_010420R:0.43433499683585480300)14:0.04657423732482594836,Nvec_NVE25349:0.48023994002681441584)19:0.05046716394228628610,Skow_XP_002741140:0.30579916300795112472)15:0.05721416549952946379)93:0.37432933872418516774)13:0.03401146588929346293,(Scil_13054:0.67616667404703645339,(Aque_2_1_37513_001:0.68949978802022959812,(Aque_2_1_39650_001:0.65276211840725029134,Aque_2_1_39648_001:0.39410639706143218985)53:0.07464571591733915046)98:0.20630364802899009424)41:0.17596484577398330273)3:0.03690557266116543944,((((Cmil_SINCAMP00000015602:0.21392927701433531174,(Hsap_ENSP00000359630:0.05511856021484700524,Drer_ENSDARG00000059832:0.03414885464456116071)93:0.02507081681460098313)100:0.68687833020439847953,Nvec_NVE12353:1.02116902689738564547)49:0.24065925909100782665,((Bbel_260260F:0.48956860934691637244,((((Cint_gi_XP_009862007:0.34815383986370224134,Lgig_154761:0.32893446966705530699)100:0.37493879301599697396,Spur_WHL22.392553:0.91686569897491054792)20:0.10664050304879697795,(Nvec_NVE10940:0.55060126099708994740,Adig_2760v120555:0.53482131117867881542)68:0.20487550462630518822)11:0.05875487565255963635,Nvec_NVE13836:0.52093985297506806287)7:0.05990764670791638019)8:0.09859029679193032081,(((Tadh_XP_002113682:0.60050346737261217633,(Tadh_XP_002113325:0.52460437915314550583,Tadh_XP_002117818:0.65145622453978757616)43:0.07587967266825194901)44:0.1047650473177887858,
(Drer_ENSDARG00000091757:1.17896725624596521165,Spur_WHL22.71613:0.38156655447935855507)36:0.30237373901526537479)15:0.13217450328887561661,((Nvec_NVE9578:0.47102497413047722530,Nvec_NVE16489:0.52996337524285574982)31:0.09098213548897930714,(Skow_XP_002735837:0.43438477811670317408,Adig_11540v108276:0.44120529424267845853)26:0.09264910904286217941)9:0.05854005157771656476)3:0.08962838127794467424)1:0.01203352977537435441)0:0.04073801435008328475,((Nvec_NVE18487:0.53297564842695333009,Adig_19406v115301:0.60452344862688545124)26:0.09296812937046926295,(Bbel_292050F:0.65442887093944124288,(Skow_XP_002740941:0.37784137947517015821,Skow_XP_002734724:0.44702806138121331525)94:0.18494305565178020823)49:0.10446186381944129462)6:0.07419612180500807785)0:0.03525811901423607508)0:0.07400213783273368318,(((Bbel_214760R:1.28939293696587586879,Bbel_270220R:1.17643466700118182189)58:0.27422975037217622862,(Adig_8886v105801:0.79978182489093951890,Nvec_NVE22429:0.74488734667944977996)43:0.12120000402932988581)12:0.05576801018384930364,Aque_2_1_37130_001:0.97721929671898888348)3:0.03120243680747190190)3:0.04507701769346997289,(Tadh_XP_002118234:0.28814695045275223739,Tadh_XP_002111670:0.25143701540747037670)100:0.70248906000377231429)33:0.11752390354172161102,((Sros_PTSG_09542:0.06065377775411177896,Sros_PTSG_09543:0.03146890947481194994)100:0.46717833839912925598,Sros_PTSG_06041:0.49594051916091791421)100:0.49157400935025769018)40:0.09274100851612636365,((Ocar_g3634_t1:0.57972696714679128327,Aque_2_1_20823_001:0.67998794785372285165)42:

0.09407634089392304111,((((Scil_7737:0.00000093904650884574,Scil_14656:0.00000093904650884574)100:0.91437694626379
628637,Scil_50471:0.69851879652502280038)49:0.19485562523367155885,((Scil_289:0.00000093904650884574,Scil_227:0.0000
0093904650884574)100:0.73685086884952910591,(Scil_14083:0.83233937176467898134,((Scil_27094:0.2882050415580524327
4,(((Scil_68080:0.00000093904650884574,Scil_49553:0.00000093904650884574)100:0.22730743831384547793,(Scil_70266:0.35
991385249934881863,(Scil_41456:0.23752123845300490701,Scil_63570:0.21431553088787608230)81:0.07765027909313260146
)81:0.09377594926895456429)100:0.40743035628865581499,Scil_9494:0.52830390683270000984)28:0.03134687710023535689)
42:0.05115305768981971007,Scil_47986:0.66108030892001123924)71:0.16427513729371978624)90:0.20302797955047047207)
52:0.08385009649070575277)16:0.12936201340526690129,((((Nvec_NVE6184:0.20465724597212242575,Adig_16756v113082:0.
25939728537586753676)100:0.52868239326815724688,((Nvec_NVE23935:0.40828485959597382582,Adig_17884v114019:0.331
95647088033192196)100:0.29433450429106816637,Hmag_XP_002158889:0.75800618219524495967)25:0.05714870462139035739)4:0.05076331632955653067,(((Cint_gi_XP_002129016:0.91986702207832371503,(Hsap_ENSP00000273352:0.920166428247
99843581,
((Cint_gi_XP_004225576:0.96641116064927723439,(Cint_gi_XP_009858460:0.49332773748826558302,(Cint_gi_XP_009858263:0
.41942556988295798259,Cint_gi_XP_009860041:0.28178582163391258097)62:0.16977101514787507064)48:0.14488676267837
624634)32:0.07686463558859799039,Cint_gi_XP_002124051:0.78086526237606823653)96:0.20818571044324338248)57:0.1499
4604731643779161)9:0.08288248228152543529,(((Bbel_299040F:0.10181235382058119454,((Bbel_281940F:0.034997739194028
31418,Bbel_079450F:0.06558120562375324702)98:0.03575163880047449450,Bbel_121130R:0.08506063411706986377)62:0.015
98470350286807298)100:0.14872426840485516997,(Bbel_060890R:0.14026730539116635810,Bbel_204650R:0.19487521923456
818662)84:0.07087718813991801536)100:0.46110394644426710986,((Bbel_238310R:0.09146117161513664384,(Bbel_130640F:
0.01013389539588382092,Bbel_230420R:0.00867542845275841475)99:0.04640562603644749623)95:0.04767283795908030392,
(Bflo_gi_XP_002598501:0.02681525476242640343,(Bbel_156760R:0.09621245257517389848,(Bbel_063250R:0.00000093904650
884574,Bbel_062450F:0.00000093904650884574)100:0.01370851901835210170)77:0.01670687491154971421)67:0.02852800086
008724229)100:0.27019340877728381667)28:0.06104924984340030780)3:0.04286771772518304519,(Skow_XP_002740291:0.43
181917116192430894,((((Spur_WHL22.264380:0.03092326046684661447,((Spur_WHL22.226322:0.03006045384879451124,Spur
_WHL22.138572:0.05203314575455216667)55:0.01405657916782714414,Spur_WHL22.230519:0.02038865713382750910)80:0.0
3724168756500093047)96:0.11301996001607865494,((Spur_WHL22.147647:0.04324474149290333247,Spur_WHL22.184504:0.0
7770914261713493532)41:0.01092514153366807433,(Spur_WHL22.383522:0.05546708220459532768,Spur_WHL22.754506:0.03
104940057667930284)70:0.01066891624527460322)98:0.15277113308946085879)100:0.66262280973696974229,Spur_WHL22.7
3425:0.54534406259750256574)38:0.10450841352413869911,(Aque_2_1_28798_001:1.03292691331755381867,(Spur_WHL22.3
020:0.36677961175238049663,Spur_WHL22.3487:0.13379089555517253984)100:0.29173291811999318979)15:0.1703580901254
4736452)12:0.11886526004000080858)8:0.05024158721407705197)7:0.04321632268173600244)6:0.13447668761675957150,(((C

int_ENSCINP00000004627:0.58879982567518052150,(Drer_ENSDARG00000087813:0.32734229877998638480,((Hsap_ENSP00
000359686:0.28734813314311136168,Cmil_SINCAMP00000023038:0.30410884467517063090)82:0.08434091855420175476,((C
mil_SINCAMP00000004297:0.83845070315121716931,(Hsap_ENSP00000356581:0.19762749723008390945,Drer_ENSDARG000
00054137:0.09599701847198034710)100:0.10135953977913375601)32:0.05814738922255023590,((Cmil_SINCAMP00000022659
:0.20867578769224601198,Hsap_ENSP00000369198:0.19731089272970706028)42:0.05399557323771495693,
(Drer_ENSDARG00000088937:0.17204009557393087904,Drer_ENSDARG00000088137:0.17310167314851052089)100:0.139191
57192913023113)51:0.07110716563343714991)21:0.04299033988947070783)28:0.07191469612234500930)99:0.3061812036893
6413422)78:0.10344255200523430616,(Bbel_144770R:0.51997965723866956811,(Skow_XP_002733570:0.08343522170812557
92,((Skow_XP_002734798:0.03960691140292443846,(Skow_XP_002733572:0.02072166822797665009,Skow_XP_002735776:0.0
4928114392837549401)46:0.00960417044236843744)53:0.01309258545355020641,Skow_XP_002733569:0.03746440654257787
023)78:0.03167451613232664248)100:0.33967449517335296383)46:0.04881658481774957259)74:0.09112277488149884974,((L
gig_74363:0.28938049174073010583,Cgig_10006507:0.28251455664456925909)73:0.06916700550370545597,(Smar_003150:0.2
6670758187418114327,Dpul_EFX80180:0.52954090763390471164)95:0.17813074805629761088)100:0.34977529868060097673)
59:0.08228111736357288097)6:0.08975430633992298457)2:0.03605932525214040202)6:0.09437593616666750673)1:0.0240863
3917761420981)7:0.06560782318879640473,((Sros_PTSG_07638:1.34177266638395131793,(((Nvec_NVE21116:0.132432252904
51685061,Adig_11959v108662:0.43377763006135555690)99:0.14707792092099555847,Hmag_XP_002158964:0.5089364344153
9027084)61:0.08565599861689045247,(Bbel_108220F:0.36779869152364780271,((Bbel_057670F:0.33550323342265453430,(Aq
ue_2_1_38404_001:1.20128020534659407659,Scil_184:0.64201333394050297265)90:0.27087866670498955690)32:0.120503861
09947926194,((Pmar_ENSPMAG00000003119:0.30383459822027836239,(Hsap_ENSP00000296619:0.17790289097724035217,(
Cmil_SINCAMP00000019338:0.12616088644911188643,(Drer_ENSDARG00000089584:0.01590561650304854469,Drer_ENSDAR
G00000021137:0.00000093904650884574)100:0.23778236021771842479)34:0.04931911407130495223)100:0.168646236958663
57012)100:0.27531293151462549185,(Spur_WHL22.718158:0.34104946622023812886,((Cgig_10005042:0.318639222714061750
05,Ctel_219079:0.43935905552305043464)92:0.17143620663905392854,Skow_NP_001161550:0.18370175258162260712)64:0.0
8728975812845840565)32:0.05830754285413523863)17:0.04453626304014372123)27:0.06526484108698811515)25:0.03079152
082232473539)96:0.61730094880264707591)91:0.42090998505157267706,(Tcas_EFA07473:0.68002766103978673495,(Dmel_N
P_651842:0.22187926490415374459,Dmel_NP_651845:0.54927841922812237208)100:0.38488583779753338110)100:0.6615233
9299722595634)5:0.08404858751171330467)19:0.03223211957288282109)37:0.12035402476408865735)81:0.542162439049696
18761)5:0.11207673904792585240)4:0.04666379047455240792)32:0.13290756835851003093)51:0.28864787968977201338)60:0.
24570277867329834431)35:0.20435463642541529250,(Tcas_Mthl16:1.23975051970146754599,(Pxyl_XP_011552150:0.64877282
719358264629,(Dvir_XP_002060099.1:0.10308259443650844012,Dmel_Mthl15:0.15196671925092383737)100:0.73998971908618
971227)43:0.12356974410815312060)26:0.11200831957967144481)

21:0.12772911754769963877,(((Dpul_EFX89687:0.54037628369087620239,(Methuselah_Dpul_EFX89685:0.16484226539336521
711,Dpul_EFX89686:0.31691399392374708377)94:0.10043163919528007300)79:0.07774874734263625964,((Dpul_EFX83013:0.0
4056719719431503557,(Dpul_EFX83097:0.09073635142132191467,Dpul_EFX83094:0.04770257494621904515)57:0.0292655747
9801391443)100:0.70932862636512283849,Dpul_EFX74305:0.59676508059576738319)84:0.13210627340767458482)99:0.35947
281805833430912,((Smar_012256:1.04737619122750036382,(Asia_JW965784:0.42546658951240329261,(Asia_JW969407:0.047
21867758770408735,Asia_JW948170_JW948387:0.12676016595145195298)100:0.18401292325729359800)100:0.334332499856
70110416)49:0.11922263970098637142,((Dvir_Mthl1_XP_002059559.1:0.12953125971111290049,Dmel_Mthl1:0.11969346387670
946885)100:0.24433820894481850239,Tcas_Mthl1:0.38516302785375056139)100:0.74404313473541638935)8:0.0379914192302
9503848)18:0.08869406175313872265)13:0.06669239986649302954)38:0.21120093269852932560)56:0.27240802236057004571,
((Dmel_Mthl9:0.05828449494350674415,Dvir_Mthl9_XP_002046453.1:0.14435899139331681562)100:1.10404125086119142551,(
Dmel_Mthl8:0.08368306845957561302,Dvir_Mthl8_XP_002046452.1:0.18385615485391973434)100:1.01412626622015555888)96
:0.86630619398912056539)93:0.20813935319586737704)55:0.13454762615668516834)46:0.05910262266167833761,((Dvir_XP_
002046457.1:0.38933453664442541076,((Dvir_Mthl11_XP_002053031.1:0.35046757110056064866,Dvir_Mthl11_XP_002060419.1
:0.77090221448770301915)92:0.14996454440598772084,Dmel_Mthl11:0.62627195011558633997)100:0.30809315856544955414)
47:0.06493236489677052525,(((Dmel_Mthl7:0.45515787577339289482,(Dmel_Mthl12:0.44087732190528550191,Dmel_Mthl13:0.7
6733337430212344987)63:0.09640830737493220892)100:0.23005222737851041548,Dmel_Mthl6:0.44144862639664766979)100:
0.36707831104856447624,((Dmel_Mthl3:0.03359198892503823669,Dmel_Mthl4:0.15489154005570346384)100:0.2517786931190
8518402,(Dmel_methuselah:0.11998893869678939339,Dmel_Mthl2:0.32081242689566846771)93:0.11008515332802015774)98:0.
17456712153363582174)89:0.07918231095845032486)100:0.37369074696753906695)100:0.30082600240060414221,Ceratitis:0.1
8594086957833602658);

Table S1: Genomes included in the database and species codes.

Species	Abbreviation	Phylum (Class)	Kingdom	Presence of Mth GPCRs
Acropora digitifera	Adig	Cnidaria	Animalia	no
Adineta vaga	Avag	Rotifera	Animalia	no
Aedes aegypti	Aaeg	Arthropoda	Animalia	yes
Amphimedon queenslandica	Aque	Porifera	Animalia	no
Apis mellifera	Amel	Arthropoda	Animalia	yes
Bombyx mori	Bmor	Arthropoda	Animalia	yes
Branchiostoma belcheri	Bbel	Chordata	Animalia	yes
Caenorhabditis elegans	Cele	Nematoda	Animalia	no
Callorhinchus milii	Cmil	Chordata	Animalia	no
Capitella teleta	Ctel	Annelida	Animalia	yes
Capsaspora owczarzaki	Cowc	-	Filozoa	no
Ciona intestinalis	Cint	Chordata	Animalia	no
Crassostrea gigas	Cgig	Mollusca	Animalia	yes
Creolimax fragrantissima	Cfra			no
Danio rerio	Drer	Chordata	Animalia	no
Daphnia pulex	Dpul	Arthropoda	Animalia	yes
Drosophila melanogaster	Dmel	Arthropoda	Animalia	yes

Echinococcus multilocularis		**Platyhelminthes**	**Animalia**	**no**
Helobdella robusta	**Hrob**	**Annelida**	**Animalia**	**no**
Homo sapiens	**Hsap**	**Chordata**	**Animalia**	**no**
Hydra magnipapillata	**Hmag**	**Cnidaria**	**Animalia**	**no**
Lottia gigantea	**Lgig**	**Mollusca**	**Animalia**	**yes**
Ministeria vibrans	**Mvib**		**Filozoa**	**no**
Mnemiopsis leidyi	**Mlei**	**Ctenophora**	**Animalia**	**no**
Monosiga brevicollis	**Mbre**	***Choanoflagellatea***	**Filozoa**	**no**
Nematostella vectensis	**Nvec**	**Cnidaria**	**Animalia**	**yes**
Oscarella carmela	**Ocar**	**Porifera**	**Animalia**	**no**
Petromyzon marinus	**Lamp**	**Chordata**	**Animalia**	**no**
Saccoglossus kowalevskii	**Skow**	**Hemichordata**	**Animalia**	**yes**
Salpingoeca rosetta	**Sros**	***Choanoflagellatea***	**Filozoa**	**no**
Schistosoma mansoni	**Sman**	**Platyhelminthes**	**Animalia**	**no**
Sphaeroforma arctica	**Sarc**			**no**
Strigamia maritima	**Smar**	**Arthropoda**	**Animalia**	**yes**
Strongylocentrotus purpuratus	**Spur**	**Echinodermata**	**Animalia**	**yes**
Sycon ciliatum	**Scil**	**Porifera**	**Animalia**	**no**
Tetranychus urticae	**Turt**	**Arthropoda**	**Animalia**	**no**
Tribolium castaneum	**Tcas**	**Arthropoda**	**Animalia**	**yes**
Trichinella spiralis	**Tspi**	**Nematoda**	**Animalia**	**no**
Trichoplax adhaerens	**Tadh**	**Placozoa**	**Animalia**	**no**

Table S2: Reciprocal BLAST results. Bolted: High confidence Mth/Mthl homologs (see text for definition). Color code: Yellow = arthropod homologs; grey = non-arthropod homologs.

Gene	Species	Taxon	Drosophila melanogaster: Best reBLAST match	E-value	Branchiostoma floridae: Best reBLAST match	E-value
Cgig_10015721	Crassostrea gigas	Mollusca	mth	7.00E-14	XP_002610765	7.00E-21
Bbel_132690R	Branchiostoma belcheri	Chordata: Amphioxiformes	Mthl1	1.00E-09	XP_002601306	0
Dpul_EFX67288	Daphnia pulex	Arthropods: Crustacea	Mthl1	2.00E-16	XP_002608881	1.00E-20
Bbel_069800F	Branchiostoma belcheri	Chordata: Amphioxiformes	starry night	0.047	XP_002609978	0
Smar_012256	Strigamia maritima	Arthropods: Myriapoda	Mthl1	2.00E-29	XP_002598501	2.00E-20
Bbel_134080R	Branchiostoma belcheri	Chordata: Amphioxiformes	furrowed	1.00E-17	XP_002598404	0
Bbel_145670F	Branchiostoma belcheri	Chordata: Amphioxiformes	furrowed	1.00E-15	XP_002588254	0
Bbel_192410R	Branchiostoma belcheri	Chordata: Amphioxiformes	megalin	2.00E-17	XP_002588254	0
Bbel_293770F	Branchiostoma belcheri	Chordata: Amphioxiformes	phospholipase C at 21C	1.00E-06	XP_002598404	3.00E-153
Asia_JW948170_JW948387	Argulus_siamensis	Arthropods: Crustacea	Mthl10	2.00E-56	XP_002610765	4.00E-21

Asia_JW965784	Argulus_siamensis	Arthropods: Crustacea	Mthl10	9.00E-43	XP_002598501	1.00E-19
Cbol_GAYO01008982	Cordulegaster_boltonii	Arthropods: Insecta: Odonata	Mthl10	8.00E-50	XP_002610765	4.00E-19
Cbol_GAYO01127990	Cordulegaster_boltonii	Arthropods: Insecta: Odonata	Mthl1	2.00E-32	XP_002610765	1.00E-19
Cbol_GAYO01128358	Cordulegaster_boltonii	Arthropods: Insecta: Odonata	Mthl10	2.00E-45	XP_002598501	2.00E-17
Asia_JW969407	Argulus_siamensis	Arthropods: Crustacea	Mthl10	5.00E-51	XP_002610765	1.00E-18
Bflo_XP_002610765	Branchiostoma floridae	Chordata: Amphioxiformes	Mthl10	2.00E-13	not applicable	not applicable
Cgig_10001367	Crassostrea gigas	Mollusca	Mthl10	1.00E-11	XP_002610765	5.00E-18
Cgig_10021040	Crassostrea gigas	Mollusca	Mthl10	4.00E-18	XP_002609978	9.00E-20
Dpul_EFX74305	Daphnia pulex	Arthropods: Crustacea	Mthl10	1.00E-35	XP_002609978	5.00E-12
Dpul_EFX89686	Daphnia pulex	Arthropods: Crustacea	Mthl10	9.00E-44	XP_002608881	5.00E-14
Dpul_EFX89687	Daphnia pulex	Arthropods: Crustacea	Mthl10	1.00E-35	XP_002610765	2.00E-16
Lgig_75877	Lottia gigantea	Mollusca	Mthl10	5.00E-12	XP_002608881	5.00E-21
Dmel_methuselah	Drosophila melanogaster	Arthropods: Insecta: Diptera			XP_002598608	4.00E-07
Dmel_Mthl1	Drosophila melanogaster	Arthropods: Insecta: Diptera			XP_002609206	1.00E-14

**Dmel_Mthl10**	**Drosophila melanogaster**	**Arthropods: Insecta: Diptera**			**XP_002610765**	**2.00E-01**
**Dmel_Mthl11**	**Drosophila melanogaster**	**Arthropods: Insecta: Diptera**			**XP_002610765**	**2.00E-09**
**Dmel_Mthl12**	**Drosophila melanogaster**	**Arthropods: Insecta: Diptera**			**AHB53231**	**0.003**
**Dmel_Mthl13**	**Drosophila melanogaster**	**Arthropods: Insecta: Diptera**			**XP_002610765**	**8**
**Dmel_Mthl14**	**Drosophila melanogaster**	**Arthropods: Insecta: Diptera**			**XP_002588913**	**0.03**
**Dmel_Mthl15**	**Drosophila melanogaster**	**Arthropods: Insecta: Diptera**			**XP_002587041**	**0.85**
**Dmel_Mthl2**	**Drosophila melanogaster**	**Arthropods: Insecta: Diptera**			**XP_002610765**	**2.00E-11**
**Dmel_Mthl3**	**Drosophila melanogaster**	**Arthropods: Insecta: Diptera**			**no significant hit**	**no significant hit**
**Dmel_Mthl4**	**Drosophila melanogaster**	**Arthropods: Insecta: Diptera**			**XP_002610765**	**8.00E-13**
Dmel_Mthl5	***Drosophila melanogaster***	***Arthropods: Insecta: Diptera***			***XP_002608881***	***0.05***
**Dmel_Mthl6**	**Drosophila melanogaster**	**Arthropods: Insecta: Diptera**			**no significant hit**	**no significant hit**
**Dmel_Mthl7**	**Drosophila melanogaster**	**Arthropods: Insecta: Diptera**			**XP_002610765**	**9.00E-05**
**Dmel_Mthl8**	**Drosophila melanogaster**	**Arthropods: Insecta: Diptera**			**no significant hit**	**no significant hit**
**Dmel_Mthl9**	**Drosophila melanogaster**	**Arthropods: Insecta: Diptera**			**no significant hit**	**no significant hit**

Nvec_XP_001636770	Nematostella vectensis	Cnidaria	Mthl10	6.00E-18	XP_002610765	1.00E-34
Nvec_XP_001640473	Nematostella vectensis	Cnidaria	Mthl10	3.00E-20	XP_002610765	6.00E-35
Nvec_XP_001628572	Nematostella vectensis	Cnidaria	Mthl10	3.00E-19	XP_002610765	1.00E-37
Spur_XP_011679633	Strongylocentrotus purpuratus	Hemichordata	Mthl10	3.00E-17	XP_002610765	4.00E-31
Spur_gi_XP_011675990	Strongylocentrotus purpuratus	Hemichordata	Mthl11	5.00E-13	XP_002610765	2.00E-34
Spur_gi_XP_798567	Strongylocentrotus purpuratus	Hemichordata	Mthl11	4.00E-12	XP_002610765	4.00E-34
Spur_XP_011660493	Strongylocentrotus purpuratus	Hemichordata	Mthl11	7.00E-13	XP_002609978	6.00E-30
Ctel_208644	Capitella teleta	Annelida	Mthl15	1.00E-02	XP_002609978	7.00E-49
Ctel_216805	Capitella teleta	Annelida	Mthl15	8.00E-25	XP_002609978	5.00E-45
Ctel_63867	Capitella teleta	Annelida	Mthl15	9.00E-28	XP_002609978	2.00E-32
Dpul_EFX73784	Daphnia pulex	Arthropods: Crustacea	Mthl15	1.00E-32	XP_002608881	2.00E-31
Esup_GAVW01003911	Epiophlebia_superstes	Arthropods: Insecta: Odonata	Mthl10	3.00E-66	XP_002610765	1.00E-24
Dpul_EFX73786	Daphnia pulex	Arthropods: Crustacea	Mthl15	4.00E-24	XP_002608881	2.00E-29
Dpul_EFX79407	Daphnia pulex	Arthropods: Crustacea	Mthl15	5.00E-17	XP_002610765	8.00E-43

Mrot_XP_0037031 17	Megachile rotundata	Arthropods: Insecta: Hymenoptera	Mthl15	3.00E-5 8	XP_002598501	4.00E-12
Dpul_EFX89685	Daphnia pulex	Arthropods: Crustacea	Mthl15	1.00E-4 3	XP_002610765	1.00E-18
Gdib_GASB01015 634	Glycera dibranchiata	Annelida	Mthl15	1.00E-2 4	XP_002609978	1.00E-33
Skow_XP_006818 657	Saccoglossus kowalevskii	Hemichordata	Mthl15	4.00E-1 6	XP_002608881	6.00E-45
Pxyl_XP_0115521 50_Mth2like	Plutella_xylostella	Arthropods: Insecta: Lepidoptera	Mthl15	2.00E-4 5	XP_002586351	0.002
Skow_XP_006821 924	Saccoglossus kowalevskii	Hemichordata	Mthl15	4.00E-1 8	XP_002610765	2.00E-50
Spur_XP_0116620 57	Strongylocentrotus purpuratus	Hemichordata	Mthl15	5.00E-2 2	XP_002610765	5.00E-34
Cgig_10025895	Crassostrea gigas	Mollusca	Mthl3	2.00E-1 0	XP_002608881	6.00E-20
Dpul_EFX83013	Daphnia pulex	Arthropods: Crustacea	Mthl3	3.00E-3 6	XP_002610765	2.00E-14
Dpul_EFX83094	Daphnia pulex	Arthropods: Crustacea	Mthl3	1.00E-3 2	XP_002613279	1.00E-06
Dpul_EFX83097	Daphnia pulex	Arthropods: Crustacea	Mthl3	4.00E-3 5	XP_002589269	8.00E-11
Cgig_10006654	Crassostrea gigas	Mollusca	Mthl4	4.00E-1 5	XP_002609978	1.00E-19
Spur_XP_0037239 96	Strongylocentrotus purpuratus	Hemichordata	Mthl4	4.00E-2 8	XP_002610765	7.00E-28

Bflo_gi_XP_002608881	Branchiostoma floridae	Chordata: Amphioxiformes	Mthl5	3.60E-02	not applicable	not applicable
Tcas_Mthl1	*Tribolium castaneum*	*Arthropods: Insecta: Coleoptera*	*Mthl1*	*5.00E-123*	*XP_002608881*	*7.00E-14*
Tcas_Mthl15	Tribolium castaneum	Arthropods: Insecta: Coleoptera	Mthl15	2.00E-55	XP_002598501	3.00E-13
Tcas_Mthl16	*Tribolium castaneum*	*Arthropods: Insecta: Coleoptera*	*Mthl15*	*2.00E-26*	*XP_002608881*	*1.00E-13*
Tcas_Mthl5	Tribolium castaneum	Arthropods: Insecta: Coleoptera	Mthl5	1.00E-154	-	-
Tcas_Mthlsc	Tribolium castaneum	Arthropods: Insecta: Coleoptera	Mthl10	1.00E-55	XP_002610765	5.00E-14

Table S3: Spreadsheet of Mth/Mthl gene family members with information on taxonomic affiliation and detectable Pfam domains. Color code: Light green = Detection of Methuselah_N domain supported with Pfam e-values <10E-50; Darkest grey shade = Detection of Methuselah_N domain supported with Pfam e-values between E-40 and E-15; Medium grey shade = Detection of Methuselah_N domain supported with Pfam e-values between E-10 and E-5; Lightest grey shade = Detection of Methuselah_N domain supported with Pfam e-values between E-5 and 0.05; No shade = lack of significant Methuselah_N domain support;

Gene	Species	Taxon	Pfam e-value Methuselah_N domain	Pfam e-value Somatomedin B domain	Pfam e-value Kringle domain	Pfam e-value Lectin C-type domain	Pfam e-value PLC-beta C terminal	Pfam e-value CUB domain
Dmel_methuselah	Drosophila melanogaster	Arthropods: Insecta: Diptera	3.10E-69	-	-	-	-	-
Dmel_Mthl2	Drosophila melanogaster	Arthropods: Insecta: Diptera	1.40E-68	-	-	-	-	-
Dvir_XP_002046457.1	Drosophila virilis	Arthropods: Insecta: Diptera	7.30E-66	-	-	-	-	-
Dmel_Mthl3	Drosophila melanogaster	Arthropods: Insecta: Diptera	6.90E-64	-	-	-	-	-
Dmel_Mthl4	Drosophila melanogaster	Arthropods: Insecta: Diptera	7.10E-64	-	-	-	-	-
Dmel_Mthl11	Drosophila melanogaster	Arthropods: Insecta: Diptera	1.20E-63	-	-	-	-	-
Dvir_Mthl10_XP_002046455.1	Drosophila virilis	Arthropods: Insecta: Diptera	4.50E-63	-	-	-	-	-
Dvir_Mthl11_XP_002053031.1	Drosophila virilis	Arthropods: Insecta: Diptera	1.20E-60	-	-	-	-	-

Dmel_Mthl6	Drosophila melanogaster	Arthropods: Insecta: Diptera	2.50E-59	-	-	-	-	-
Dmel_Mthl10	Drosophila melanogaster	Arthropods: Insecta: Diptera	1.60E-58	-	-	-	-	-
Dvir_Mthl8_XP_002046452.1	Drosophila virilis	Arthropods: Insecta: Diptera	4.30E-57	-	-	-	-	-
Dmel_Mthl9	Drosophila melanogaster	Arthropods: Insecta: Diptera	3.90E-56	-	-	-	-	-
Dmel_Mthl7	Drosophila melanogaster	Arthropods: Insecta: Diptera	5.10E-56	-	-	-	-	-
Dvir_Mthl9_XP_002046453.1	Drosophila virilis	Arthropods: Insecta: Diptera	2.50E-54	-	-	-	-	-
Ccap_XP_012157161.1_Mth2like	Ceratitis_capitata	Arthropods: Insecta: Diptera	2.50E-52	-	-	-	-	-
Dmel_Mthl8	Drosophila melanogaster	Arthropods: Insecta: Diptera	6.80E-52	-	-	-	-	-
Mdom_XP_005175058.1_Mth2like	Musca domestica	Arthropods: Insecta: Diptera	1.20E-50	-	-	-	-	-
Bdor_XP_011201382.1_Mth2like	Bactrocera_dorsalis	Arthropods: Insecta: Diptera	1.30E-50	-	-	-	-	-
Dmel_Mthl12	Drosophila melanogaster	Arthropods: Insecta: Diptera	9.80E-50	-	-	-	-	-
Dvir_Mthl11_XP_002060419.1	Drosophila virilis	Arthropods: Insecta: Diptera	5.50E-37	-	-	-	-	-
Agam_XP_316287.2	Anopheles gambiae	Arthropods: Insecta: Diptera	3.90E-24	-	-	-	-	-
Dmel_Mthl13	Drosophila melanogaster	Arthropods: Insecta: Diptera	8.90E-18	-	-	-	-	-

Tcas_Mthlsc	Tribolium castaneum	Arthropods: Insecta: Coleoptera	1.60E-09	-	-	-	-	-
Mrot_XP_003703117	Megachile rotundata	Arthropods: Insecta: Hymenoptera	8.90E-09	-	-	-	-	-
Esup_GAVW01003911	Epiophlebia_superstes	Arthropods: Insecta: Odonata	1.10E-07	-	-	-	-	-
Pxyl_XP_011552150.1_Mth2like	Plutella_xylostella	Arthropods: Insecta: Lepidoptera	1.70E-06	-	-	-	-	-
Cbol_GAYO01008982	Cordulegaster_boltonii	Arthropods: Insecta: Odonata	8.10E-05	-	-	-	-	-
Dpul_EFX89685	Daphnia pulex	Arthropods: Crustacea	8.80E-05	-	-	-	-	-
Dmel_Mthl15	Drosophila melanogaster	Arthropods: Insecta: Diptera	0.00012	-	-	-	-	-
Asia_JW948170_JW948387	Argulus_siamensis	Arthropods: Crustacea	-	-	-	-	-	-
Asia_JW965784	Argulus_siamensis	Arthropods: Crustacea	-	-	-	-	-	-
Asia_JW969407	Argulus_siamensis	Arthropods: Crustacea	-	-	-	-	-	-
Bbel_132690R	Branchiostoma belcheri	Chordata: Amphioxiformes	-	-	-	-	-	-
Bflo_gi_XP_002608881	Branchiostoma floridae	Chordata: Amphioxiformes	-	2.80E-05	-	-	-	-
Bflo_XP_002610765.1	Branchiostoma floridae	Chordata: Amphioxiformes	-	-	-	-	-	-
Cbol_GAYO01127990	Cordulegaster_boltonii	Arthropods: Insecta: Odonata	-	-	-	-	-	-

Cbol_GAYO01128358	Cordulegaster_boltonii	Arthropods: Insecta: Odonata	-	-	-	-	-	-
Cgig_10001367	Crassostrea gigas	Mollusca	-	-	-	-	-	-
Cgig_10006654	Crassostrea gigas	Mollusca	-	-	-	-	-	-
Cgig_10015721	Crassostrea gigas	Mollusca	-	-	-	-	-	-
Cgig_10021040	Crassostrea gigas	Mollusca	-	-	-	-	-	-
Cgig_10025895	Crassostrea gigas	Mollusca	-	-	-	-	-	-
Ctel_208644	Capitella teleta	Annelida	-	-	-	-	-	-
Ctel_216805	Capitella teleta	Annelida	-	-	-	-	-	-
Ctel_63867	Capitella teleta	Annelida	-	-	-	-	-	-
Dmel_Mthl1	Drosophila melanogaster	Arthropods: Insecta: Diptera	-	-	-	-	-	-
Dmel_Mthl14	Drosophila melanogaster	Arthropods: Insecta: Diptera	-	-	-	-	-	-
Dmel_Mthl5	Drosophila melanogaster	Arthropods: Insecta: Diptera	-	-	-	-	-	-
Dpul_EFX67288	Daphnia pulex	Arthropods: Crustacea	-	-	-	-	-	-
Dpul_EFX73784.1	Daphnia pulex	Arthropods: Crustacea	-	-	-	-	-	-
Dpul_EFX73786.1	Daphnia pulex	Arthropods: Crustacea	-	-	-	-	-	-
Dpul_EFX74305	Daphnia pulex	Arthropods: Crustacea	-	-	-	-	-	-
Dpul_EFX79407.1	Daphnia pulex	Arthropods: Crustacea	-	-	-	-	-	-

Dpul_EFX83013	Daphnia pulex	Arthropods: Crustacea	-	-	-	-	-	-
Dpul_EFX83094	Daphnia pulex	Arthropods: Crustacea	-	-	-	-	-	-
Dpul_EFX83097	Daphnia pulex	Arthropods: Crustacea	-	-	-	-	-	-
Dpul_EFX89686	Daphnia pulex	Arthropods: Crustacea	-	-	-	-	-	-
Dpul_EFX89687	Daphnia pulex	Arthropods: Crustacea	-	-	-	-	-	-
Dvir_Mthl1_XP_002059559.1	Drosophila virilis	Arthropods: Insecta: Diptera	-	-	-	-	-	-
Dvir_Mthl14_XP_002060308.1	Drosophila virilis	Arthropods: Insecta: Diptera	-	-	-	-	-	-
Dvir_Mthl5_XP_002053335.1	Drosophila virilis	Arthropods: Insecta: Diptera	-	-	-	-	-	-
Dvir_XP_002060099.1	Drosophila virilis	Arthropods: Insecta: Diptera	-	-	-	-	-	-
Gdib_GASB01015634	Glycera dibranchiata	Annelida	-	-	-	-	-	-
Lgig_75877	Lottia gigantea	Mollusca	-	-	-	-	-	-
Nvec_XP_001636770	Nematostella vectensis	Cnidaria	-	-	-	-	-	-
Nvec_XP_001640473	Nematostella vectensis	Cnidaria	-	1.30E-07	-	-	-	-
Nvec_XP_001628572	Nematostella vectensis	Cnidaria	-	1.80E-08	-	-	-	-
Skow_XP_006818657	Saccoglossus kowalevskii	Hemichordata	-	-	-	-	-	-
Skow_XP_006821924.1	Saccoglossus kowalevskii	Hemichordata	-	9.60E-07	-	-	-	-
Smar_012256	Strigamia maritima	Arthropods: Myriapoda	-	-	-	-	-	-

Spur_gi_XP_011675990	Strongylocentrotus purpuratus	Hemichordata	-	-	-	-	-	-
Spur_gi_XP_798567	Strongylocentrotus purpuratus	Hemichordata	-	-	-	-	-	-
Spur_XP_003723996	Strongylocentrotus purpuratus	Hemichordata	-	-	-	-	-	-
Spur_XP_011660493	Strongylocentrotus purpuratus	Hemichordata	-	-	-	-	-	-
Spur_XP_011662057	Strongylocentrotus purpuratus	Hemichordata	-	-	-	-	-	-
Spur_XP_011679633.1	Strongylocentrotus purpuratus	Hemichordata	-	-	-	-	-	1.60E-16
Tcas_Mthl1	Tribolium castaneum	Arthropods: Insecta: Coleoptera	-	-	-	-	-	-
Tcas_Mthl15	Tribolium castaneum	Arthropods: Insecta: Coleoptera	-	-	-	-	-	-
Tcas_Mthl16	Tribolium castaneum	Arthropods: Insecta: Coleoptera	-	-	-	-	-	-
Tcas_Mthl5	Tribolium castaneum	Arthropods: Insecta: Coleoptera	-	-	-	-	-	-
